# Evaluation of Four Commonly Used DNA Barcoding Loci for Chinese Medicinal Plants of the Family Schisandraceae

**DOI:** 10.1371/journal.pone.0125574

**Published:** 2015-05-04

**Authors:** Jian Zhang, Min Chen, Xiaoyu Dong, Ruozhu Lin, Jianhua Fan, Zhiduan Chen

**Affiliations:** 1 State Key Laboratory of Systematic and Evolutionary Botany, Institute of Botany, Chinese Academy of Sciences, Beijing, P.R. China; 2 Graduate University of the Chinese Academy of Sciences, Beijing, P.R. China; 3 Institute of Botany, Jiangsu Province and Chinese Academy of Sciences/Nanjing Botanical Garden Memorial Sun Yat-Sen, Nanjing, Jiangsu, P.R. China; 4 Research Institute of Forest Ecology, Environment and Protection, Chinese Academy of Forestry, Beijing, P.R. China; Kunming Institute of Botany, CHINA

## Abstract

Many species of Schisandraceae are used in traditional Chinese medicine and are faced with contamination and substitution risks due to inaccurate identification. Here, we investigated the discriminatory power of four commonly used DNA barcoding loci (ITS, *trnH-psbA*, *matK*, and *rbcL*) and corresponding multi-locus combinations for 135 individuals from 33 species of Schisandraceae, using distance-, tree-, similarity-, and character-based methods, at both the family level and the genus level. Our results showed that the two spacer regions (ITS and *trnH-psbA*) possess higher species-resolving power than the two coding regions (*matK* and *rbcL*). The degree of species resolution increased with most of the multi-locus combinations. Furthermore, our results implied that the best DNA barcode for the species discrimination at the family level might not always be the most suitable one at the genus level. Here we propose the combination of ITS+*trnH-psbA*+*matK*+*rbcL* as the most ideal DNA barcode for discriminating the medicinal plants of *Schisandra* and *Kadsura*, and the combination of ITS+*trnH-psbA* as the most suitable barcode for *Illicium* species. In addition, the closely related species *Schisandra rubriflora* Rehder & E. H. Wilson and *Schisandra grandiflora *Hook.f. & Thomson, were paraphyletic with each other on phylogenetic trees, suggesting that they should not be distinct species. Furthermore, the samples of these two species from the southern Hengduan Mountains region formed a distinct cluster that was separated from the samples of other regions, implying the presence of cryptic diversity. The feasibility of DNA barcodes for identification of geographical authenticity was also verified here. The database and paradigm that we provide in this study could be used as reference for the authentication of traditional Chinese medicinal plants utilizing DNA barcoding.

## Introduction

The need for specimen identification on the basis of DNA sequences has been increasingly recognized. Accordingly, DNA barcoding, a rapid technique for the identification of biological specimens using short DNA sequences from either the nuclear genome or organellar genomes has been proposed [1]. DNA barcoding could not only help with the identification of specimens, but also define species boundaries and discover new or cryptic species that are difficult, or sometimes impossible, to distinguish morphologically [2–4]. The technique is also beneficial to the authentication of various medicinal plants [5,6] and the revelation of cryptic diversity [2,7–9]. In recent years, different single loci and combined loci have been proposed as plant DNA barcodes [6]. In 2009, the Consortium for the Barcode of Life Plant Working Group (CBOL) proposed a combination of *matK* and *rbcL* as a ‘core barcode’ for plant identification across land plants [10]. Furthermore, the nuclear ribosomal internal transcribed spacer (ITS) region [11–13] and the plastid intergenic spacer (*trnH-psbA*) region have also been proposed as supplementary barcodes for land plants [14,15]. In particular, ITS2 was proposed as a core DNA barcode for medicinal plants [16] and the combination of ITS2 and *trnH-psbA* was suggested as a preliminary system for DNA barcoding of herbal materials [5]. ITS, *trnH-psbA*, *matK*, and *rbcL* are the top four barcoding regions mentioned in the literatures for the authentication and identification of medicinal plant materials reviewed by Techen et al. [6].

Schisandraceae are a family of the order Austrobaileyales, with the center of diversity in China [17–20]. This family is composed of three genera, *Schisandra* Michx., *Kadsura* Kaempf. ex Juss., and *Illicium* L. [21]. There are 25 species in *Schisandra*, 22 in *Kadsura*, and 42 in *Illicium* [17]. Except for one species of *Schisandra* and five species of *Illicium* distributed in North America, all the other species of Schisandraceae are distributed in China and/or its neighbouring countries of southeastern Asia [17–20]. Many species of Schisandraceae, including 16 species of *Schisandra*, eight species of *Kadsura*, and 16 species of *Illicium*, have been used in traditional Chinese medicine for many years for the purposes of increasing physical working capacity, relieving pain, and treating skin inflammation [22–29]. In particular, the fruits of *Schisandra chinensis* (Turcz.) Baill. (Wu Wei Zi), *S*. *sphenanthera* Rehder & E. H. Wilson (Nan Wu Wei Zi), and *Illicium verum* Hook. f. (Ba Jiao Hui Xiang), are well-known ingredients accepted by Chinese Pharmacopoeia 2010 [30]. Most species used as folk medicine are found to contain types of chemical components that exhibit various beneficial bioactivities, such as anti-HIV, anti-cancer, and anti-hepatitis [26,29,31,32]. The contents of these components differ in various species, resulting in different clinical pharmacological effects [25].

For traditional medicine, the bark, roots and fruits are commonly used [33]. These parts do not provide enough morphological variation to accurately identify species in Schisandraceae. Floral characters that are important for taxonomic classification, especially in *Schisandra* and *Kadsura*, might be lost or ignored during the collection process. Therefore, undesired species could be inadvertently collected, if target species are easily confused with their close relatives. Inferior substitutes and adulterants could affect patient safety and the drug’s efficacy [5,34,35]. For example, the comestible Chinese star anise (*Illicium verum*), as a medicinal tea, is sometimes contaminated with the highly toxic Japanese star anise (*I*. *anisatum* L.), since these two species possess similar fruit morphology. The contaminated star anise teas result in serious neurological and gastrointestinal symptoms for users [27,34,36]. For this reason, the U.S. Food and Drug Administration (FDA) issued a warning against star anise teas on September 10, 2003 (http://www.fda.gov/ICECI/EnforcementActions/EnforcementStory/EnforcementStoryArchive/ucm095929.htm). The fruits of different *Schisandra* species in different geographic regions are all traditionally treated as the medicinal ‘Wu Wei Zi’, because of similar fruit morphology and taste [28]. However, the medicinal value of different species in *Schisandra* has been found to differ significantly [25]. The classification systems before APG III segregated the genus *Illicium* as a distinct family, Illiciaceae, and left *Schisandra* and *Kadsura* in the family Schisandraceae sensu stricto [37–39]. Furthermore, the molecular phylogenetic analyses to date concluded that neither *Schisandra* nor *Kadsura* is monophyletic [40–45]. In addition, the infra-generic classifications in Schisandraceae are still unstable, and species boundaries have not been resolved thoroughly [17–20, 46–53]. Therefore, the specimen identifications of Schisandraceae by feasible and reliable methods are crucial for the precise utility of medicinal plants.

Until now, only a few DNA barcoding studies referred to medicinal plants in Schisandraceae. A study of the authentication of *Illicium verum* and its seven adulterants showed that *trnH-psbA* could distinguish *I*. *verum* from other adulterating species, compared to the other three commonly used loci (ITS2, *matK*, and *rbcL*) [54]. Furthermore, ITS2 and ITS distinguish *Schisandra chinensis* from *S*. *sphenanthera* [55], and *S*. *sphenanthera* from its adulterant *S*. *viridis* A.C.Sm. [56], respectively. Given that these studies only referred to a minority of species from *Illicium* or *Schisandra*, a deeper and more comprehensive molecular authentication of medicinal plants in Schisandraceae covering all three genera is needed.

In this study, we focused on plants with medicinal properties from all three genera in Schisandraceae and investigated the applicability and effectiveness of four commonly used DNA barcoding loci (ITS, *trnH-psbA*, *matK*, and *rbcL*), either alone or in combination for species discrimination using distance-, tree-, similarity-, and character-based methods, at both the family level and the genus level. The two regions of ITS (ITS1-5.8S-ITS2), ITS1 and ITS2, were also included in the analyses, in order to compare the discriminatory power of Schisandraceae species among them. Our objectives were: (1) to identify which commonly used barcoding locus or multi-locus combination would be the most ideal barcode for authenticating the medicinal plants of Schisandraceae; (2) to develop a DNA barcode database for these medicinal plants based on the comparison of the discriminatory ability of four loci and/or their combinations; (3) to initially reveal the cryptic diversity within Schisandraceae species and scrutinize the feasibility of DNA barcodes for identification of the geographical authenticity of medicinal plants.

## Materials and Methods

### Plant materials

A total of 33 species (14 of *Schisandra*, six of *Kadsura*, and 13 of *Illicium*) were included in this study, of which 27 are used in traditional Chinese medicine (S1 Table). With the exception of *Kadsura ananosma* Kerr, at least two individuals were sampled for each species. We sampled 135 individuals, including 58 from *Schisandra*, 27 from *Kadsura*, and 50 from *Illicium* (S1 Table). Among them, 110 specimens were newly collected and taxonomically identified using published floras, monographs, and references [17–20, 46–53]. All these specimens were collected from the wild and no specific permissions were required for the corresponding locations/activities, and the locations did not include any national park or other protected area of land. The field studies did not involve endangered or protected species. Sequences from other species were retrieved from GenBank (http://www.ncbi.nlm.nih.gov/genbank/) and/or previous studies after careful quality assessment [40,41,43,54,56–65]. The singleton species (species represented by one individual) (Table 1) were only used as potential causes of failed discrimination, but not included in the calculation of identification success rate [66,67]. *Austrobaileya scandens* C. T. White, a member of Austrobaileyaceae (a sister group of Schisandraceae) [21] was selected as an outgroup for tree-based analyses.

**Table 1:** Sequence characteristics of six DNA regions of Schisandraceae (Outgroup taxon excluded).

	ITS1	ITS2	ITS	*trnH-psbA*	*matK*	*rbcL*
Universality of primers	-	-	Yes	Yes	Yes	Yes
Percentage PCR success (%)	-	-	98.19	100	100	99.09
Percentage sequencing success (%)	-	-	100	100	100	100
No. of species (no. of individuals)	33 (123)	33 (123)	33 (123)	33 (114)	33 (114)	32 (118)
No. of singleton species	1	1	1	8	5	5
Aligned sequence length (bp)	299	229	695	579	826	672
Parsimony-informative sites (bp)	98	67	170	85	60	27
Variable sites (bp)	104	77	188	94	65	29
No. of indels (length range)	26 (1–23)	9 (1–2)	36 (1–23)	41 (1–46)	2 (6)	0
Average interspecific distance (range) (%)^1^	15.09 (0–29.47)	10.75 (0–21.65)	9.88 (0–19.08)	6.96 (0–14.12)	2.84 (0–5.91)	1.41 (0–2.80)
Average intraspecific distance (range) (%)^1^	0.15 (0–0.51)	0.28 (0–2.70)	0.17 (0–1.10)	0. 09 (0–0.84)	0.04 (0–0.34)	0.02 (0–0.11)
Average interspecific distance (range) (%)^2^	3.28 (0–6.98)	3.16 (0–5.96)	2.47 (0–4.87)	2.44 (0–7.05)	0.86 (0–1.85)	0.66 (0–1.19)
Average intraspecific distance (range) (%)^2^	0.15 (0–0.51)	0.10 (0–0.71)	0.11 (0–0.30)	0.15 (0–0.84)	0.06 (0–0.34)	0.03 (0–0.11)
Average interspecific distance (range) (%)^3^	2.10 (0–4.73)	1.74 (0–4.05)	1.47 (0–2.86)	1.04 (0–2.26)	0.13 (0–0.49)	0.13 (0–0.30)
Average intraspecific distance (range) (%)^3^	0.13 (0–0.41)	0.54 (0–2.70)	0.26 (0–1.10)	0.02 (0–0.13)	0	0.01 (0–0.10)

^1^The distance based on the data from Schisandraceae.

^2^The distance based on the data from *Schisandra* and *Kadsura*.

^3^The distance based on the data from *Illicium*.

### DNA extraction, amplification and sequencing

Total genomic DNA was extracted from specimens by grinding silica-gel dried-leaf tissue in liquid nitrogen, and then using the CTAB procedure [68]. Total genomic DNA was dissolved in TE buffer (10 mM Tris–HCl, pH 8.0, 1 mM EDTA) to a final concentration of 30–60 ng/μL. Polymerase chain reaction (PCR) amplification of targeted DNA regions was performed using 2×Taq PCR MasterMix (Biomed, Beijing, China), which containing 0.05 u/μL of Taq DNA Polymerase, 4 mM MgCl_2_, 0.4 mM of dNTP and reaction buffer. The PCR mix included 12.5 μL 2×Taq PCR MasterMix, 2 μL each primer (5 μM), 1–2 μL template DNA and enough distilled deionized water to give a final volume of 25 μL. The primer information and optimal PCR conditions are displayed in S2 Table [69–74]. PCR products were examined electrophorectically using 0.8% agarose gels. The PCR products were purified using BioMed multifunctional DNA fragment purification recovery kits (Beijing, China), and then were sequenced using the amplification primers. The bidirectional sequencing was completed using the ABI 3730 DNA Sequencer (Applied Biosystems, Carlsbad, California, USA).

### Sequence alignment

The quality estimation and assembly for the newly generated sequences were performed with ContigExpress 6.0 (Invitrogen, Carlsbad, California, USA). All the newly acquired sequences were confirmed via BLASTn (http://blast.ncbi.nlm.nih.gov/Blast.cgi) against the online nucleotide database and further deposited in GenBank. The accession numbers of new sequences and published sequences included in this study are provided in S1 Table. The sequence alignment for each locus was initially performed by using MUSCLE [75], and then manually edited in GeneDoc 2.7.0 [76]. The number of indel events for each dataset was inferred by deletion/insertion polymorphisms (DIP) analysis in DnaSP v5 [77]. In the DIP analysis, indels of different lengths, even in the same position of the alignment, are treated as different events. Because of the high divergence of ITS sequences among different plant families, only the 5.8S rDNA from the outgroup species could be aligned with ingroup sequences. Since the *trnH-psbA* sequences of other family are too divergent to be aligned with the sequences of Schisandraceae, the *trnH-psbA* sequences of the outgroup species were not used in the analysis. In further analyses, both family-level and genus-level assessments of the discriminatory power for single regions and their combinations were included. For the genus-level assessment, *Illicium* and *Schisandra/Kadsura* were analyzed independently, because *Illicium* is quite different from *Schisandra* and *Kadsura* according to species morphology [37–39] and sequence data. Since neither *Schisandra* nor *Kadsura* is monophyletic based on previous phylogenetic studies, these two genera were not separated into independent analyses [40–45].

### Data analysis

#### Distance-based analysis

**Fig 1:**
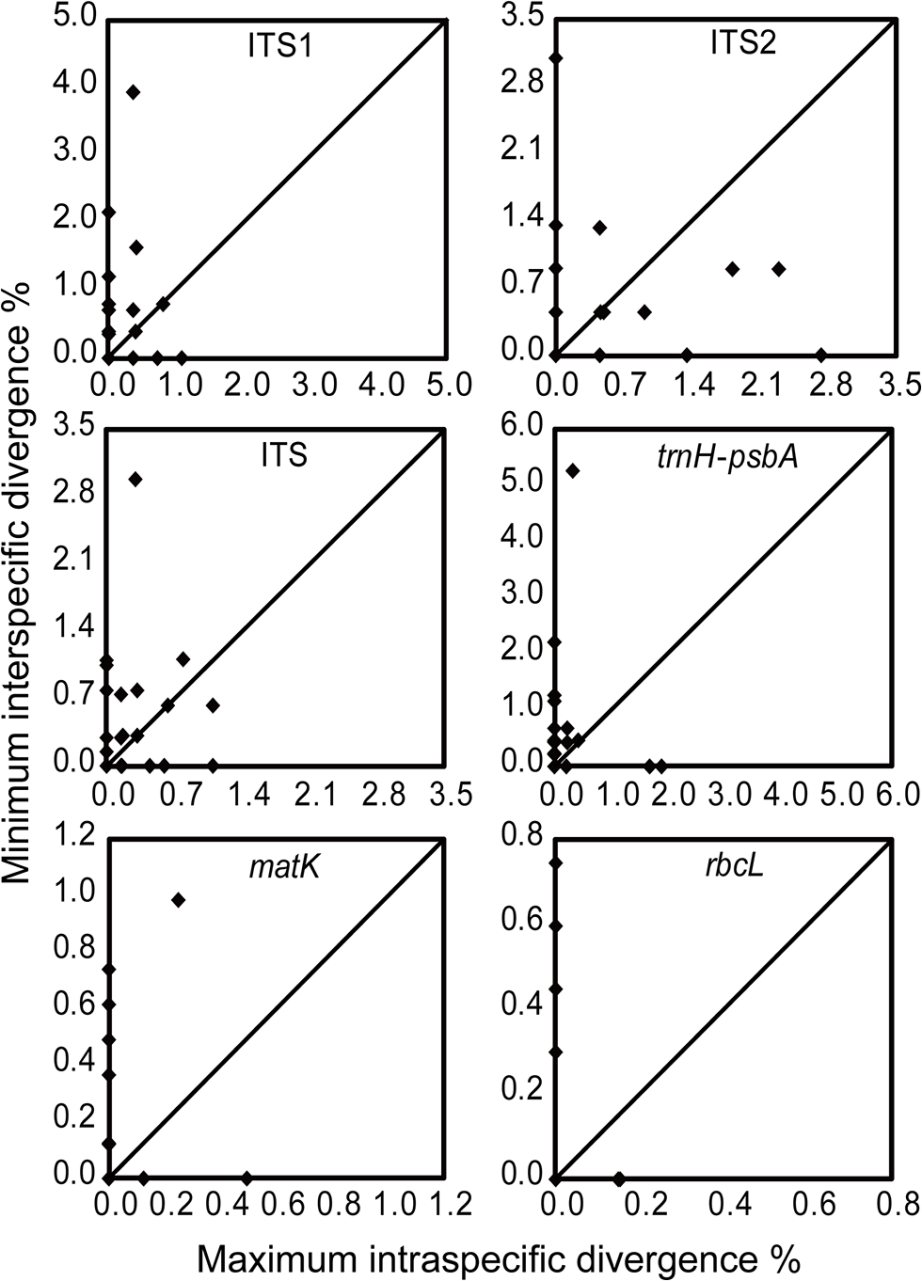
Plots of maximum intraspecific vs. minimum interspecific p-distances for single regions of Schisandraceae. Each dot represents a species for which two or more individuals were sampled. Dots above the diagonal line indicate the presence of a barcoding gap.

**Fig 2:**
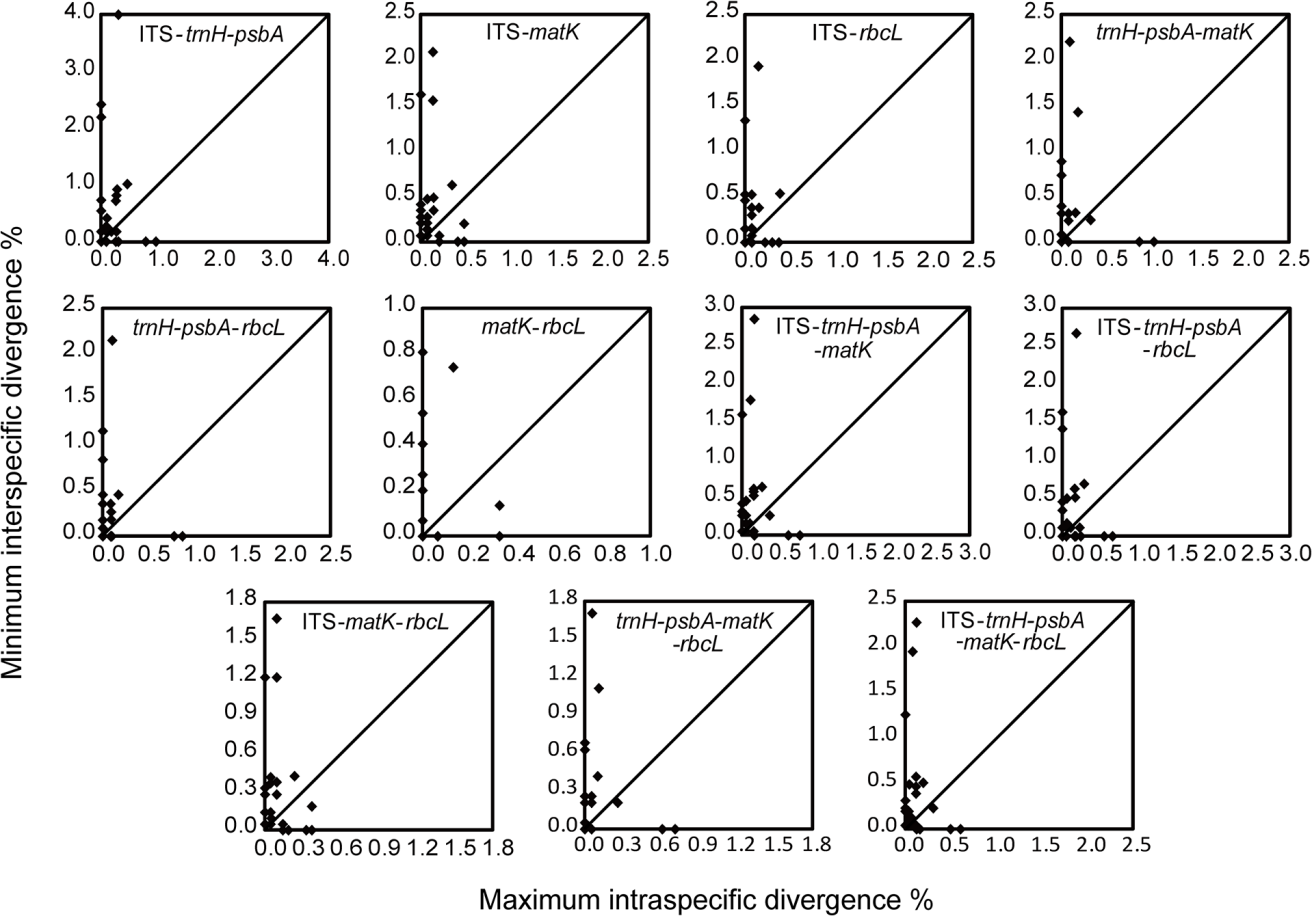
Plots of maximum intraspecific vs. minimum interspecific p-distances for multi-locus combinations of Schisandraceae. Each dot represents a species for which two or more individuals were sampled. Dots above the diagonal line indicate the presence of a barcoding gap.

We calculated genetic distances for each DNA region using MEGA v5.05 [78] based on the uncorrected p-distance model, which has been shown to perform as well as or better than the broadly used Kimura-2-parameter model [79–81]. The pairwise distances, intra- and interspecific distances were calculated for each species that were represented by more than one individual. Additionally, the differences of intra- and interspecific divergences between each pair of four commonly used barcoding loci were tested by Wilcoxon signed-ranks tests [7,15] in PASW Statistics 18.0 (IBM, Armonk, New York, USA). To assess the differences between intra- and interspecific divergences within each commonly used barcoding locus, Wilcoxon two-sample tests were performed. For each species, the minimum interspecific distances were compared with maximum intraspecific distances in order to detect the presence of a barcoding gap [82,83]. In Figs 1 and 2, the dot above the 1:1 slope indicates the presence of a barcoding gap for the species, whereas the dot below the 1:1 slope implies no barcoding gap [81,84].

#### Tree-based analysis

Phylogenetic trees were constructed for each single region and various multi-locus combinations using maximum-likelihood (ML) and Bayesian-inference (BI) methods in order to assess whether species are recovered as monophyletic. The percentage of the monophyletic clusters for individuals belonging to the same species was calculated. For model-based phylogenetic methods (ML and BI), the best-fitting model for each dataset was determined by the Akaike Information Criterion (AIC) in jModelTest 2.1.4 [85]. ML and BI analyses were carried out by running RAxML-HPC2 7.6.3 on XSEDE [86] and MrBayes 3.2.2 on XSEDE [87] respectively at the CIPRES Science Gateway (http://www.phylo.org/). The bootstrap values of ML trees were assessed by 1000 replicates of heuristic searches [88]. For BI analyses, four Markov chain Monte Carlo (MCMC) chains were run for 10,000,000 generations until the average deviation of split frequencies was below 0.01. The 50% majority-rule consensus trees were constructed after the first 25% of sampled trees were removed during the burn-in period. The posterior probability (PP) of each topological bipartition was calculated across remaining trees. There were no strongly supported topological conflicts (i.e., incongruences with bootstrap values ≥70% for ML, and posterior probabilities ≥0.95 for BI) among the phylogenies of individual loci, so they could be combined in the further analyses.

#### Similarity-based analysis

Furthermore, we measured the proportion of correct identification using ‘best match’ and ‘best close match’ methods in TAXONDNA based on the uncorrected p-distances, which could determine the closest match of a sequence by comparing it to all other sequences in the aligned data set [89]. The analyses require species to be represented by two or more individuals. For the ‘best close match’ method, the threshold similarity values were computed from the pairwise summary, in order to define how similar a barcode match needs to be before it can be identified [89]. The criteria for successful identification, ambiguous identification, incorrect identification, and no match were set according to previous studies [89,90].

#### Character-based analysis

The search for diagnostic characters during the single-locus assessment was performed using the web-based CAOS (Characteristic Attribute Organization System) workbench (http://boli.uvm.edu/caos-workbench/caos.php) [91]. Aligned DNA sequences and ML trees were imported into Mesquite v2.76 [92] and exported as NEXUS file formats for the CAOS-Analyzer. The outputs of the CAOS-Analyzer were used for the CAOS-Barcoder in order to find ‘characteristic attributes’ (CAs) (character-based diagnostics), including pure characters (existing across all members of a clade but never in any other clade) and private characters (existing across some members of a clade but never in any other clade). Nucleotide positions at which pure CAs and private CAs shared in at least 80% of all members within a group were included in the calculation. Both simple CAs (confined to a single nucleotide position) and compound CAs (combined states at multiple nucleotide positions) were considered [93].

## Results

### Amplification and sequence analysis

The four commonly used barcoding loci performed equally well in terms of the universality of amplification and sequencing (Table 1). There were 437 new sequences generated in this study: 108 ITS, 110 *trnH-psbA*, 110 *matK*, and 109 *rbcL* (S1 Table). Including the sequences from GenBank and previous studies, in grand total, 123 ITS, 114 *trnH-psbA*, 114 *matK*, and 118 *rbcL* sequences of Schisandraceae species were included in this study and summarized in Table 1. In addition, the multi-copy problem for ITS in plants reviewed by Nietto & Rosello [94] was not present in this study. Among four commonly used barcoding loci, ITS showed the highest percentage of parsimony informative sites (24.46%), followed by *trnH-psbA* (14.68%), *matK* (7.26%), and *rbcL* (4.02%) (Table 1). Most of the parsimony informative sites for ITS came from the ITS1 and ITS2 regions (97.06%), and ITS1 provided more informative sites than IT S2 (Table 1). Indels were more prevalent in *trnH-psbA* and ITS alignments, compared with *matK* and *rbcL* alignments (Table 1).

### Distance-based identification

Among four commonly used barcoding loci, ITS had the highest average interspecific divergence (0.0988 for the whole family, 0.0247 for *Schisandra* and *Kadsura*, 0.0147 for *Illicium*), while *trnH-psbA* was at an intermediate level of variation, but higher than both *matK* and *rbcL* at both the family level and the genus level (Table 1). The average intraspecific divergence was the highest for ITS (0.0017 for the whole family, 0.0026 for *Illicium*), followed by *trnH-psbA*, *matK*, and *rbcL* for the family as a whole and for *Illicium* alone (Table 1). In contrast, *trnH-psbA* had the highest average intraspecific divergence (0.0015), followed by ITS, *matK*, and *rbcL* for *Schisandra* and *Kadsura* (Table 1). Among ITS, ITS1, and ITS2, ITS1 exhibited the highest level of interspecific divergence (0.1509 for the whole family, 0.0328 for *Schisandra* and *Kadsura*, 0.0210 for *Illicium*) at both the family level and the genus level (Table 1). ITS1 had the lowest level of intraspecific variation (0.0015 for the whole family, 0.0013 for *Illicium*) for the family as a whole and for *Illicium* alone, while ITS2 had the lowest level of intraspecific variation (0.0010) for *Schisandra* and *Kadsura* (Table 1). And the interspecific divergence of genus-level analyses was visibly lower than that of family-level analyses (Table 1). The Wilcoxon signed-rank tests further confirmed that ITS had the highest divergence at both interspecific and intraspecific levels, while *rbcL* had the lowest interspecific divergence (Table 2). The intraspecific variations for *trnH-psbA*, *matK*, and *rbcL* were similar (Table 2). The Wilcoxon two-sample tests showed that the interspecific divergence significantly exceeded the corresponding intraspecific divergence for each single locus (Table 2).

**Table 2:** Wilcoxon tests for four commonly used barcoding loci based on the data from Schisandraceae.

Wilcoxon signed-rank tests based on the interspecific and intraspecific p-distances among four barcoding loci						
W+	W-	Relative ranks		n	*p*-value ≤	Result
		W+	W-			
interspecific distance						
ITS	*trnH-psbA*	34588	3362	275	0	ITS > *trnH-psbA*
ITS	*matK*	37853	97	275	0	ITS > *matK*
ITS	*rbcL*	61358	67	350	0	ITS > *rbcL*
*trnH-psbA*	*matK*	37348	53	275	0	*trnH-psbA* > *matK*
*trnH-psbA*	*rbcL*	43951	5	299	0	*trnH-psbA* > *rbcL*
*matK*	*rbcL*	34686	1629	275	0	*matK* > *rbcL*
intraspecific distance						
ITS	*trnH-psbA*	101	35	23	0.044	ITS > *trnH-psbA*
ITS	*matK*	141	12	23	0.001	ITS > *matK*
ITS	*rbcL*	145.5	7.5	26	0.0005	ITS > *rbcL*
*trnH-psbA*	*matK*	35	10	23	0.0695	*trnH-psbA* = *matK*
*trnH-psbA*	*rbcL*	48	18	24	0.091	*trnH-psbA* = *rbcL*
*matK*	*rbcL*	18	3	23	0.058	*matK* = *rbcL*
**Wilcoxon two-sample tests based on the interspecific versus intraspecific p-distances of four barcoding loci**						
	No. of interspecific distance		No. of intraspecific distance		W	*p*-value ≤
ITS	496		32		827.5	7.03E-20
*trnH-psbA*	300		25		478	1.63E-15
*matK*	378		28		1028.5	6.62E-15
*rbcL*	351		27		913.5	1.59E-14

The symbols “W+” and “W-” represent the sum of all of the positive values and negative values in the signed-rank column, respectively. Symbol “>” is used if the interspecific or intraspecific divergence for a locus significantly exceeds that of another locus.

No consistent presence of a barcoding gap was found for any of the included regions (Figs 1 and 2). In the species barcoding gap assessment, *trnH-psbA* showed relatively better performance than the other three loci, while *rbcL* was the worst performer in this analysis at both the family level and the genus level (Table 3 and S3 Table). In addition, ITS performed better than both ITS1 and ITS2 at the family level, as it did for *Illicium* (Table 3 and S3 Table). According to the data from the genera *Schisandra* and *Kadsura*, ITS1 performed as well as ITS (S3 Table). The multi-locus combinations, ITS+*matK*, ITS+*trnH-psbA*+*matK*, ITS+*matK*+*rbcL*, and ITS+*trnH-psbA*+*matK*+*rbcL*, performed better than others at the family level, as they did for *Schisandra* and *Kadsura* (Table 3 and S3 Table). The combinations ITS+*trnH-psbA* and ITS+*trnH-psbA* +*rbcL* also performed as well as the former ones for *Illicium* (S3 Table).

**Table 3:** Discriminatory power of single regions and their combinations based on the data from Schisandraceae.

DNA barcodes	N^*^	Ability to discriminate (distance)^#^ (%)	Ability to discriminate (ML)^$^ (%)		Ability to discriminate (BI) ^$^ (%)		Ability to discriminate (character)^&^ (%)
			I	II	I	II	
ITS1	32	37.50	43.75	25.00	40.63	21.88	34.38
ITS2	32	25.00	31.25	15.63	12.50	6.25	18.75
ITS	32	40.63	50.00	34.38	43.75	21.88	40.63
*trnH-psbA*	25	60.00	48.00	32.00	36.00	20.00	40.00
*matK*	28	39.29	35.71	32.14	28.57	14.29	25.00
*rbcL*	27	14.81	14.81	14.81	14.81	14.81	14.81
ITS+*trnH-psbA*	24	66.67	70.83	58.33	66.67	54.17	-
ITS*+matK*	24	75.00	62.50	58.33	62.50	54.17	-
ITS*+rbcL*	27	62.96	62.96	59.26	59.26	48.15	-
*trnH-psbA+matK*	24	58.33	54.17	41.67	54.17	45.83	-
*trnH-psbA+rbcL*	25	60.00	48.00	32.00	44.00	40.00	-
*matK+rbcL*	24	45.83	45.83	33.33	37.50	29.17	-
ITS*+trnH-psbA+matK*	24	75.00	70.83	62.50	66.67	66.67	-
ITS*+trnH-psbA+rbcL*	24	66.67	66.67	62.50	62.50	62.50	-
ITS*+matK+rbcL*	24	75.00	66.67	62.50	66.67	58.33	-
*trnH-psbA+matK+rbcL*	24	58.33	58.33	50.00	54.17	54.17	-
ITS*+trnH-psbA+matK+rbcL*	24	75.00	75.00	70.83	70.83	70.83	-

^*^ Species represented by multiple individuals.

^#^ The percentage of the species with higher minimum interspecific distance than maximum intraspecific distance (species barcoding gap) among studied species.

^$^ Column I: the percentage of the monophyletic clusters of individuals belonging to the same morphological species among studied species. Column II: the corresponding number for the monophyletic clusters with ≥70% bootstrap values in ML and ≥0.95 posterior probabilities in BI. ML, maximum-likelihood method; BI, Bayesian-inference method.

^&^ The percentage of the species that could be identified by diagnostic characters among studied species. The analyses did not include multi-locus combinations.

### Tree-based identification

**Fig 3:**
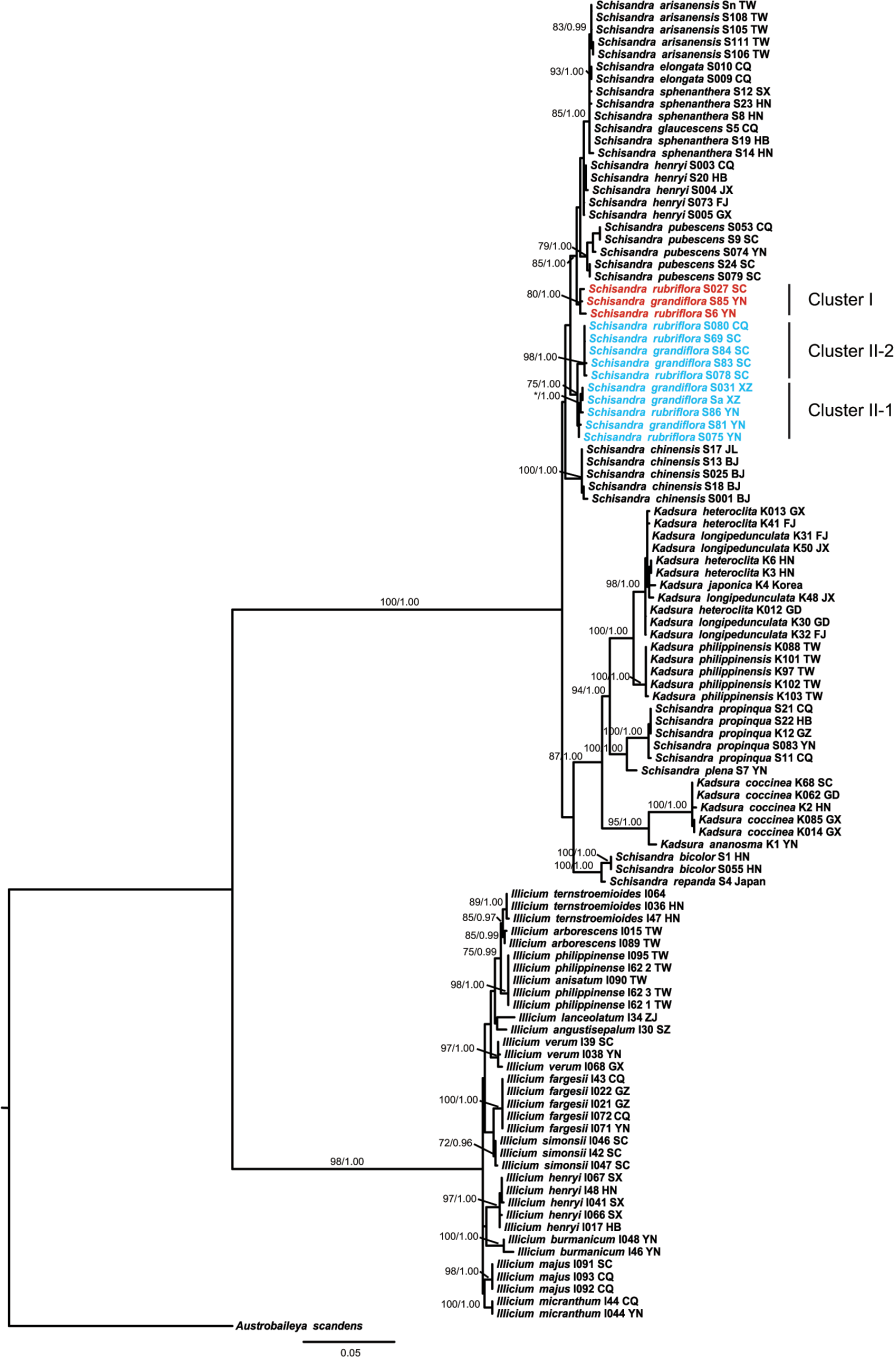
Schisandraceae ML phylogenetic tree based on the combination of ITS, *trnH-psbA*, matK, and *rbcL*. The tree included 24 species from three genera of Schisandraceae, *Schisandra*, *Kadsura*, and *Illicium*. The species *Austrobaileya scandens* was the outgroup for the analysis. All loci were available for all individuals of Schisandraceae species in the tree. Numbers above the branches represent bootstrap values for monophyletic species with ≥70% bootstrap values in ML and ≥0.95 posterior probabilities in BI. The asterisk indicates the bootstrap value or posterior probability lower than the threshold. ML, maximum-likelihood method; BI, Bayesian-inference method. The two clusters for the individuals of *Schisandra rubriflora* and *S*. *grandiflora* are labeled by different colors, red and blue, corresponding to the different sampling points (Cluster I: red, the southern Hengduan Mountains region; Cluster II: blue, the other sampling regions).

The ML phylogenetic tree of the combination of ITS+*trnH-psbA*+*matK*+*rbcL* is presented in Fig 3, and all the other phylogenetic trees are shown in S1 and S2 Figs. Among four commonly used barcoding loci, *rbcL* had the lowest discriminatory power at both the family level and the genus level (Table 3 and S3 Table). ITS showed the highest level of discrimination for the family as a whole and for *Illicium* alone (Table 3 and S3 Table). In comparison, *trnH-psbA* showed relatively better performance than the other three loci for *Schisandra* and *Kadsura* (Table 3 and S3 Table). In addition, ITS displayed higher species-resolving power than both ITS1 and ITS2 for the family as a whole and for *Illicium* alone (Table 3 and S3 Table). According to the data from the genera *Schisandra* and *Kadsura*, ITS1 and ITS performed equally well (S3 Table). In contrast, the performance of ITS2 was the worst at both the family level and the genus level (Table 3 and S3 Table). Under the ML method, the best multi-locus combination for species discrimination was ITS+*trnH-psbA*+*matK*+*rbcL*, which showed visibly higher discriminatory power than four commonly used barcoding loci, for the family as a whole and for *Schisandra* and *Kadsura* (Table 3 and S3 Table). In comparison, for *Illicium*, there were multiple best multi-locus combinations for species discrimination: ITS+*trnH-psbA*, ITS+*trnH-psbA*+*matK*, ITS+*trnH-psbA*+*rbcL*, and ITS+*trnH-psbA*+*matK*+*rbcL* (S3 Table). The results of BI analyses were similar to those of ML analyses, except for one more best combination for species discrimination, ITS+*matK*+*rbcL*, which performed as well as ITS+*trnH-psbA*+*matK*+*rbcL* for *Schisandra* and *Kadsura* (S3 Table).

Most of the four commonly used barcoding loci could only identify half or less than half of the sampled species at both the family level and the genus level, and the bootstrap re-sampling further reduced the already low identification success rates (Table 3 and S3 Table). Thus, bootstrap values were only used as a reference and not as a criterion in this study. According to the calculation of highly supported monophyletic clusters, the best combination for species discrimination was ITS+*trnH-psbA*+*matK*+*rbcL* for the family as a whole and for *Schisandra* and *Kadsura* (Table 3 and S3 Table). In comparison, ITS+*trnH-psbA*, ITS+*trnH-psbA*+*matK*, ITS+*trnH-psbA*+*rbcL*, and ITS+*trnH-psbA*+*matK*+*rbcL* worked equally well for *Illicium* (S3 Table).

### Similarity-based identification

The results of the similarity-based method at the family level and the genus level performed in TAXONDNA are shown in Table 4 and S4 Table, respectively. Among the single loci, *trnH-psbA* had the highest successful identification rate (62.26% for the whole family, 50.00% for *Schisandra* and *Kadsura*, 84.21% for *Illicium*), and *rbcL* had the lowest successful identification rate (21.23% for the whole family, 29.72% for *Schisandra* and *Kadsura*, 5.12% for *Illicium*), under the ‘best match’ method at both the family level and the genus level (Table 4 and S4 Table). The results of the ‘best close match’ method was similar with that of the ‘best match’ method, except that ITS performed better than other single loci for *Illicium* (S4 Table). Among ITS, ITS1, and ITS2, the rank order for the correct identification was ITS, ITS1, and ITS2 under the similarity-based method for the family as a whole and for *Schisandra* and *Kadsura*, while the rank was ITS, ITS2, and ITS1 for *Illicium* (Table 4 and S4 Table). Most of the multi-locus combinations displayed higher successful identification rates than single loci under the similarity-based method at both the family level and the genus level (Table 4 and S4 Table). The combination of ITS+*trnH-psbA*+*matK*+*rbcL* had the highest percentage of correct identifications (86.86% for the whole family, 80.59% for *Schisandra* and *Kadsura*, 100% for *Illicium*) under the ‘best match’ method for the family as a whole and for *Schisandra* and *Kadsura* (Table 4 and S4 Table). In comparison, ITS+*trnH-psbA*, ITS+*trnH-psbA*+*matK*, ITS+*trnH-psbA*+*rbcL*, and ITS+*trnH-psbA*+*matK*+*rbcL* had higher identification efficiency than others under the ‘best match’ method for *Illicium* (S4 Table). Under the ‘best close match’ method, there was only one different result from that of the ‘best match’ method, in which the best one for species discrimination was ITS+*rbcL* (S4 Table).

**Table 4:** Identification success rates of single regions and their combinations using TAXONDNA program under ‘best match’ and ‘best close match’ methods based on the data from Schisandraceae.

DNA barcodes	N^*^ species	N sequences	Best match (%)			Best close match (%)				Threshold (%)
			Correct	Ambiguous	Incorrect	Correct	Ambiguous	Incorrect	No match	
ITS1	32	122	50.00	49.10	0.81	49.18	49.18	0.81	0.81	0.66
ITS2	32	122	46.72	50.00	3.27	46.72	50.00	0.81	2.45	1.30
ITS	32	122	58.19	39.34	2.45	55.73	38.52	1.63	4.09	0.56
*trnH-psbA*	25	106	62.26	32.07	5.66	62.26	32.07	5.66	0.00	4.65
*matK*	28	109	40.36	59.63	0.00	40.36	59.63	0.00	0.00	0.47
*rbcL*	27	113	21.23	78.76	0.00	21.23	78.76	0.00	0.00	0.13
ITS+*trnH-psbA*	24	99	75.75	17.17	7.07	75.75	17.17	7.07	0.00	2.51
ITS+*matK*	24	99	77.77	17.17	5.05	77.77	17.17	5.05	0.00	0.46
ITS+*rbcL*	27	105	70.47	23.80	5.71	68.57	22.85	5.71	2.85	0.28
*trnH-psbA+matK*	24	101	62.37	31.68	5.94	62.37	31.68	5.94	0.00	2.27
*trnH-psbA+rbcL*	25	106	62.26	31.13	6.60	62.26	31.13	6.60	0.00	2.23
*matK+rbcL*	24	101	47.52	51.48	0.99	47.52	51.48	0.99	0.00	0.33
ITS+*trnH-psbA*+*matK*	24	99	85.85	8.08	6.06	85.85	8.08	6.06	0.00	1.70
ITS+*trnH-psbA*+*rbcL*	25	106	77.77	13.13	9.09	77.77	13.13	9.09	0.00	1.70
ITS+*matK*+*rbcL*	24	99	80.80	12.12	7.07	80.80	12.12	7.07	0.00	0.36
*trnH-psbA*+*matK*+*rbcL*	24	101	62.37	30.69	6.93	62.37	30.69	6.93	0.00	1.58
ITS*+trnH-psbA*+*matK*+*rbcL*	24	99	86.86	5.05	8.08	86.86	5.05	8.08	0.00	1.32

^*^ Species represented by multiple individuals.

### Character-based identification

Here, a set of simple pure CAs at the species level was found to be capable of distinguishing one species from the others among four single loci (ITS: three species of *Schisandra*, nine species of *Illicium*; *trnH-psbA*: three species of *Schisandra*, three species of *Kadsura*, six species of *Illicium*; *matK*: two species of *Schisandra*, two species of *Kadsura*, three species of *Illicium*; *rbcL*: two species of *Schisandra*, three species of *Kadsura*), such as ‘C’ at position 126 of ITS for *Schisandra chinensis* (S5–S8 Tables). Moreover, there were several characters that were specific to one species in a certain genus, which could be used as compound CAs after combining with nearby genus-specific diagnostic characters (ITS: two species of *Schisandra*, three species of *Illicium*; *trnH-psbA*: one species of *Schisandra*, two species of *Kadsura*; *matK*: two species of *Illicium*), such as ‘A’ at position 169 of ITS combining with the genus *Illicium* specific diagnostic character ‘G’ at position 167 for *Illicium verum* (S5–S7 Tables). In addition, indels could also be treated as diagnostic characters, especially for *trnH-psbA*, such as the species-specific insertions from position 461 to 473 of *trnH-psbA* for *S*. *propinqua* Hook.f. & Thomson (S6 Table). In the calculation of the discriminatory ability based on character-based identification, ITS and *trnH-psbA* performed nearly equally well, and *rbcL* continued to be the poorest performer at the family level (Table 3). In contrast, for *Schisandra* and *Kadsura*, *trnH-psbA* was better for species discrimination than other single loci, while for *Illicium*, ITS was better (S3 and S4 Tables)

### Species discrimination summary

Ultimately, 24 Chinese medicinal plants of Schisandraceae, nine species of *Schisandra*, three of *Kadsura*, and 12 of *Illicium* could be successfully discriminated via one or more diagnostic methods by single locus or multi-locus combinations (S9 Table). However, some species failed to be identified by all DNA regions used in this study, such as *Schisandra sphenanthera*, *S*. *rubriflora* Rehder & E.H.Wilson, *S*. *grandiflora* Hook.f. & Thomson, *Kadsura heteroclita* Craib, and *K*. *longipedunculata* Finet & Gagnep. (S9 Table). Unexpectedly, the individuals of closely related species *S*. *rubriflora* and *S*. *grandiflora* were paraphyletic with each other on phylogenetic trees (S1 and S2 Figs). Among all four single loci, the mean distances within *S*. *rubriflora* and *S*. *grandiflora* respectively were equal to or higher than the mean distances between *S*. *rubriflora* and *S*. *grandiflora* (S10 Table). Furthermore, the samples of *S*. *rubriflora* and *S*. *grandiflora* from the southern Hengduan Mountains region were distinct from the others, partitioning members of these two species into two clusters (I and II) (Fig 3 and S11 Table). Meanwhile, single nucleotide polymorphisms (SNPs) in *trnH-psbA* (12 SNPs) and *matK* (three SNPs) of *S*. *rubriflora* and *S*. *grandiflora* clearly separated these individuals into two clusters (S6 and S7 Tables). For *trnH-psbA*, *matK*, and *rbcL*, the mean distances between the two clusters were all higher than the mean distances within each cluster (S10 Table). In addition, the nucleotide variations in ITS (one SNP) and *rbcL* (one SNP) further divided cluster II into two sub-clusters, II-1 with individuals from the eastern Himalaya to the Yunnan Plateau region, and II-2 with individuals from the northeastern margin of Hengduan Mountains to the Sichuan basin region (S5 and S8 Tables). The monophyly of sub-cluster II-2 was well supported on phylogenetic trees (S1 and S2 Figs).

## Discussion

### Assessment of potential barcodes for Schisandraceae species

For the family as a whole, ITS exhibited the highest species resolution ability of the four tested loci under tree-based and character-based identifications (Table 3), and *trnH-psbA* was the best performer for species discrimination under distance-based and similarity-based identifications (Tables 3 and 4). In the genus-level evaluations, *trnH-psbA* had the highest species-resolving power for *Schisandra* and *Kadsura* under all the identification methods; ITS performed better than other single loci for *Illicium* under tree-based, character-based and similarity-based (best match method) identifications, and *trnH-psbA* was the best performer for *Illicium* under distance-based and similarity-based (best close match method) identifications (S3 and S4 Tables). These results of the genus-level evaluation explained why there were two best performers for species discrimination of the family-level evaluation. In addition, the comparison of the species-resolving power among ITS, ITS1, and ITS2, indicated that ITS performed better than both ITS1 and ITS2 at both the family level and the genus level, except that ITS1 performed as well as ITS for *Schisandra* and *Kadsura* species under distance-based, tree-based, character-based identifications (Tables 3 and 4, S3 and S4 Tables). ITS2, the core DNA barcode for medicinal plants [16] did not perform well for species discrimination in Schisandraceae (Tables 3 and 4, S3 and S4 Tables). A previous study of several *Illicium* species suggested that the species-resolving power of *trnH-psbA* was higher than ITS2, *matK*, and *rbcL* [54]. However, according to our results, the species-resolving power of ITS is better than *trnH-psbA* for *Illicium* species. The ITS region has been treated as one of the most appropriate DNA barcodes because of its higher variability, which might enhance identification rates even in closely related species [12,13]. Previous studies have suggested that ITS/ITS2 is able to discriminate several *Schisandra* species [55,56]. In addition, *trnH-psbA* has been suggested as a promising locus in many studies [14,95–98], including some on medicinal plants [99–101]. The indel polymorphisms of *trnH-psbA* seem to contribute to the species discrimination under the character-based identification (S6 Table), a result seen in other studies [102–104]. However, the species resolution ability of *trnH-psbA* has never been estimated in *Schisandra* or *Kadsura* species in previous studies.

The performance of *matK* and *rbcL* was relatively poor in respect to the species resolution ability, compared with ITS and *trnH-psbA*, on both the family-level and the genus-level evaluations (Tables 3 and 4, S3 and S4 Tables). Particularly, *rbcL* exhibited the lowest rate of species discrimination under all diagnostic methods, as well as in other studies [105,106].

In comparison with single loci, most multi-locus combinations improved the discrimination efficiency (Tables 3 and 4). Similar cases have also been reported in many other studies [7,10,15,107]. Taking all the identifications by different methods as a whole, the combination of ITS+*trnH-psbA*+*rbcL*+*matK* exhibited the best discriminatory power at both the family level and the genus level (Tables 3 and 4, S3 and S4 Tables). In medicinal plants, it has also been suggested to exhibit good discriminatory power, such as in *Angelica* L. (Apiaceae) [106]. However, taking cost and time effectiveness into account [10,108], the combination of ITS+*trnH-psbA* was the most suitable DNA barcode for identifying *Illicium* species, since it performed as well as ITS+*trnH-psbA*+*rbcL*+*matK* (S3 and S4 Tables). In previous studies, the combination of ITS and *trnH-psbA* was also proposed as the best choice for DNA identification of *Alnus* Miller species (Betulaceae) and *Parnassia* L. species (Celastraceae), respectively [109,110]. More importantly, our analyses implied that the best DNA barcode for the species discrimination at the family level might not always be the most suitable one at the genus level. In addition, the identification success rate varied among different methods, but the high low trend was similar (Tables 3 and 4, S3 and S4 Tables). The distance-based identification based on the calculation of individuals provided higher identification success rate than other identifications based on the calculation of species (Tables 3 and 4, S3 and S4 Tables).

### Species discrimination and cryptic diversity

In respect to the authentication of medicinal plants, there were 24 Chinese medicinal plants of Schisandraceae, nine species of *Schisandra*, three of *Kadsura*, and 12 of *Illicium* successfully discriminated via one or more diagnostic methods by single locus or multi-locus combinations in this study (S9 Table). Taking important medicinal plants for example, for *Schisandra chinensis*, the ITS region that could provide more diagnostic characters for this species than other regions was more suitable for its authentication (S5–S8 Tables), which has also been supported by the study of Li et al. [55]. For *Illicium verum*, ITS and *matK* were more suitable for its authentication, and they could easily distinguish this species from others using the diagnostic characters through visual examination of the alignments (S5–S8 Tables). This result was different from that of Liu M et al. [54], because we used longer sequences of ITS and *matK*, which included informative positions for distinguishing this species.

Our phylogenetic analyses indicated that both *Schisandra* and *Kadsura* were not monophyletic, and some species of *Schisandra*, such as *S*. *plena* A. C. Sm. and *S*. *propinqua*, consistently nested in the clade of *Kadsura*, although the topologies varied slightly among different DNA regions (S1 and S2 Figs). This result has also been found in many other molecular studies of Schisandraceae [40–45], which implied that the genus boundary between *Schisandra* and *Kadsura* needs to be re- examined based on both comprehensive morphological and molecular data. Furthermore, the single regions and their combinations tested in this study exhibited poor resolution for the discrimination of some species for *Schisandra* and *Kadsura* (S9 Table). These species always formed paraphyletic groups under the tree-based identification, such as *Schisandra rubriflora* and *S*. *grandiflora*, and *Kadsura heteroclita* and *K*. *longipedunculata* (S1 and S2 Figs). There are several possible reasons for gene-tree paraphyly in plants, such as imperfect taxonomy due to cryptic species complexes, incomplete lineage sorting among newly diverged species, and hybridization [111]. The unresolved species are mainly from the section *Pleiostema* of *Schisandra* and section *Eukadsura* of *Kadsura* based on the classification of Smith [17], and the species from these two groups were suggested to have diverged recently during the late Miocene to Pliocene [45]. These newly diverged species had been initially expected to exhibit paraphyletic gene trees because of incomplete lineage sorting.

*Schisandra rubriflora* and *S*. *grandiflora* are morphologically very similar, with overlap in geographical distribution ranges, and they have been incorporated into one species by Lin and Yang [51]. In this study, the individuals of these two species always grouped together on phylogenetic trees, such that the two species could not be distinguished (Fig 3 and S10 Table). Therefore, the species boundary between them was indistinct, indicating the need of comprehensive morphological observations and evaluation of additional molecular markers. Our distance-based, tree-based, and character-based analyses all supported a distinct cluster of *S*. *rubriflora* and *S*. *grandiflora* from the southern Hengduan Mountains region (Fig 3 and S6, S7, S10 Tables). Therefore, a putative cryptic species within *S*. *rubriflora* and *S*. *grandiflora* was found here. The Hengduan Mountains region, a key biodiversity hotspot in China, could provide different habitats or ecological niches that might drive the cryptic speciation [112,113]. Cryptic diversity of the species from the Hengduan Mountains region was also documented in other studies [105,113]. Further investigations into these species will be needed in order to confirm the cryptic diversity encountered by molecular analyses, especially the re-examination of morphology after more comprehensive sampling from more localities. In addition, the phylogenetic clusters and sub-clusters found in *S*. *rubriflora* and *S*. *grandiflora* were related to different geographical regions (Fig 3 and S5, S8, S10 Tables). Thus, the corresponding genetic differentiation of DNA barcodes might be feasible for the identification of geographical authenticity of these medicinal plants, as has been suggested for the species discrimination of the medicinal plants in *Angelica* L. (Apiaceae) [106].

## Conclusion

Our results indicate that the two spacer regions (ITS and *trnH-psbA*) possess higher species-resolving power than the two coding regions (*matK* and *rbcL*) in Schisandraceae. Furthermore, ITS and ITS1 performed better than ITS2 in respect to the species-resolving power. Our analyses also implied that the best DNA barcode for the species discrimination at the family level might not always be the most suitable one at the genus level. Here we proposed the combination of ITS+*trnH-psbA*+*matK*+*rbcL* as the most ideal DNA barcode for discriminating the medicinal plants of the genera *Schisandra* and *Kadsura*. In comparison, the combination of ITS+*trnH-psbA* was suggested as the most suitable DNA barcode for identifying the medicinal plants of the genus *Illicium*. Meanwhile, we recommend that people consider the discriminatory ability of DNA barcodes from both the family level and the genus level, in which studies refer to the families including several genera with quite distinct morphological and sequence characters. In addition, our analyses implied that the closely related species *Schisandra rubriflora* and *S*. *grandiflora* may not be distinct species. Moreover, a putative cryptic species was found within *S*. *rubriflora* and *S*. *grandiflora*, with a distribution in the southern Hengduan Mountains region. The feasibility of DNA barcodes for identification of geographical authenticity was also verified here. In summary, the database and paradigm that we provided in this study could be used as reference for the authentication of traditional Chinese medicinal plants utilizing DNA barcoding.

## Supporting Information

S1 Fig Schisandraceae ML phylogenetic trees based on single regions and their combinations. Numbers above the branches represent bootstrap values (≥70%) for monophyletic species. The asterisk indicates the bootstrap value or posterior probability lower than the threshold. ML, maximum-likelihood method.
(PDF)

S2 Fig Schisandraceae BI phylogenetic trees based on single regions and their combinations. Numbers above the branches represent posterior probabilities (≥0.95) for monophyletic species. The asterisk indicates the bootstrap value or posterior probability lower than the threshold. BI, Bayesian-inference method.
(PDF)

S1 Table List of samples of Schisandraceae used in this study, including species name, individual number, ID, GenBank accession number, voucher and locality information. (XLS)

S2 Table The primer information and optimal PCR conditions used in this study. (DOC)

S3 Table Discriminatory power of single regions and their combinations based on the genera data (*Schisandra*/*Kadsura* and *Illicium*). (DOC)

S4 Table Identification success rates of single regions and their combinations using TAXONDNA program under ‘best match’ and ‘best close match’ methods based on the genera data (*Schisandra*/*Kadsura* and *Illicium*). (DOC)

S5 Table Character-based DNA barcoding analysis for Schisandraceae species based on the ITS region. (XLS)

S6 Table Character-based DNA barcoding analysis for Schisandraceae species based on the *trnH-psbA* region. (XLS)

S7 Table Character-based DNA barcoding analysis for Schisandraceae species based on the *matK* region. (XLS)

S8 Table Character-based DNA barcoding analysis for Schisandraceae species based on the *rbcL* region. (XLS)

S9 Table Diagnostic barcode variation for all samples of Schisandraceae in this study. (XLS)

S10 Table The comparison of within and between group mean distances for *Schisandra rubriflora* and *S*. *grandiflora*. (DOC)

S11 Table The partition of distinct clusters for *Schisandra rubriflora* and *S*. *grandiflora* indicated in this study. (DOC)
