# Supplementary material for: Evaluation of Four Commonly Used DNA Barcoding Loci for Chinese Medicinal Plants of the Family Schisandraceae
Source: PLoS One. 2015 May 4;10(5):e0125574. doi: 10.1371/journal.pone.0125574 (PMC4418597; doi:10.1371/journal.pone.0125574)

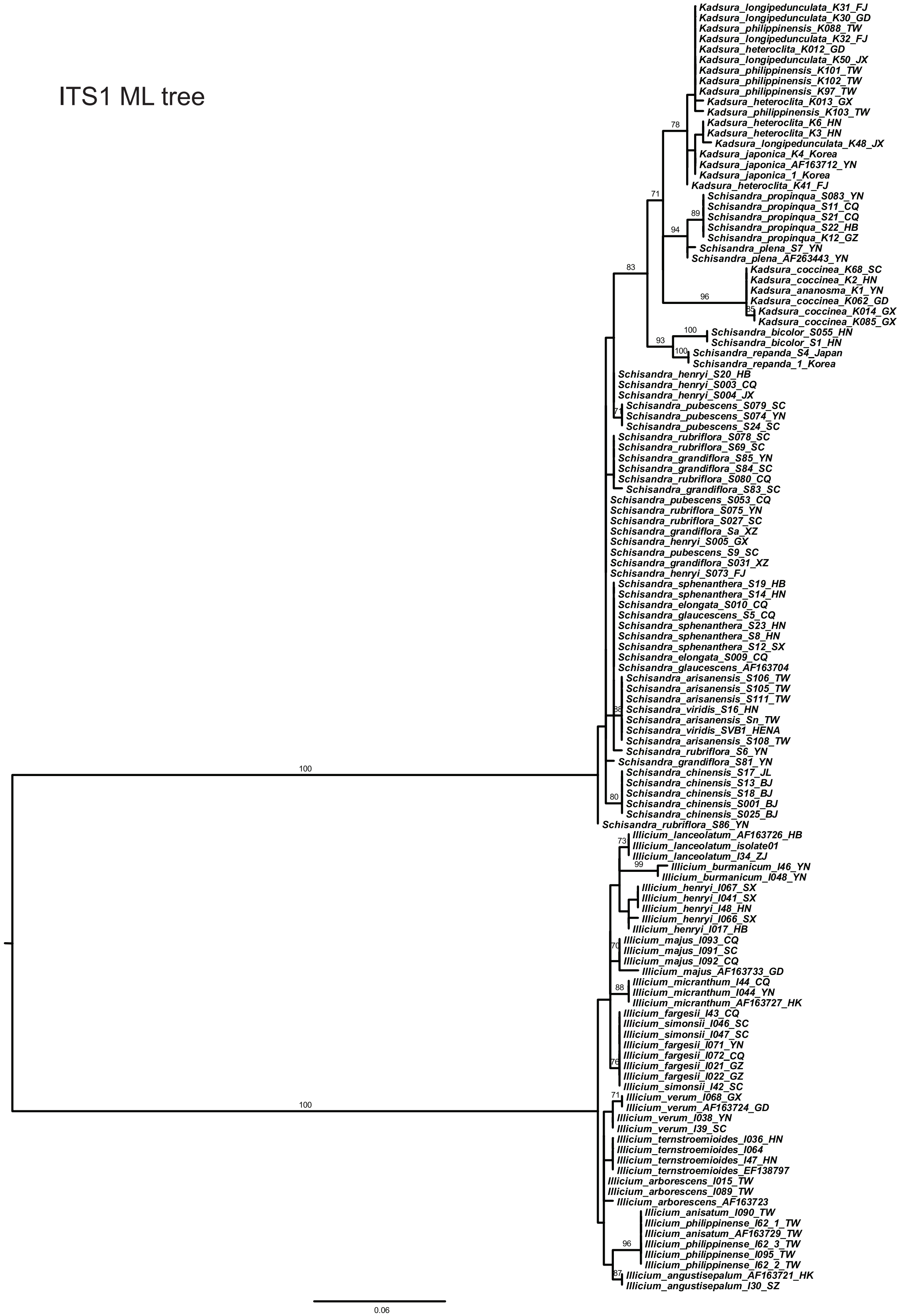

ITS1 ML tree
Kadsura_longipedunculata_K31_FJ
Kadsura_longipedunculata_K30_GD
Kadsura_philippinensis_K088_TW
Kadsura_longipedunculata_K32_FJ
Kadsura_heteroclita_K012_GD
Kadsura_longipedunculata_K50_JX
Kadsura_philippinensis_K101_TW
Kadsura_philippinensis_K102_TW
Kadsura_philippinensis_K97_TW
Kadsura_heteroclita_K013_GX
Kadsura_philippinensis_K103_TW
Kadsura_heteroclita_K6_HN
Kadsura_heteroclita_K3_HN
Kadsura_longipedunculata_K48_JX
Kadsura_japonica_K4_Korea
Kadsura_japonica_AF163712_YN
Kadsura_japonica_1_Korea
Kadsura_heteroclita_K41_FJ
Schisandra_propinqua_S083_YN
Schisandra_propinqua_S11_CQ
Schisandra_propinqua_S21_CQ
Schisandra_propinqua_S22_HB
Schisandra_propinqua_K12_GZ
Schisandra_plena_S7_YN
Schisandra_plena_AF263443_YN
Kadsura_coccinea_K68_SC
Kadsura_coccinea_K2_HN
Kadsura_ananosma_K1_YN
Kadsura_coccinea_K062_GD
Kadsura_coccinea_K014_GX
Kadsura_coccinea_K085_GX
Schisandra_bicolor_S055_HN
Schisandra_bicolor_S1_HN
Schisandra_repanda_S4_Japan
Schisandra_repanda_1_Korea
Schisandra_henryi_S20_HB
Schisandra_henryi_S003_CQ
Schisandra_henryi_S004_JX
Schisandra_pubescens_S079_SC
Schisandra_pubescens_S074_YN
Schisandra_pubescens_S24_SC
Schisandra_rubriflora_S078_SC
Schisandra_rubriflora_S69_SC
Schisandra_grandiflora_S85_YN
Schisandra_grandiflora_S84_SC
Schisandra_rubriflora_S080_CQ
Schisandra_grandiflora_S83_SC
Schisandra_pubescens_S053_CQ
Schisandra_rubriflora_S075_YN
Schisandra_rubriflora_S027_SC
Schisandra_grandiflora_Sa_XZ
Schisandra_henryi_S005_GX
Schisandra_pubescens_S9_SC
Schisandra_grandiflora_S031_XZ
Schisandra_henryi_S073_FJ
Schisandra_sphenanthera_S19_HB
Schisandra_sphenanthera_S14_HN
Schisandra_elongata_S010_CQ
Schisandra_glaucescens_S5_CQ
Schisandra_sphenanthera_S23_HN
Schisandra_sphenanthera_S8_HN
Schisandra_sphenanthera_S12_SX
Schisandra_elongata_S009_CQ
Schisandra_glaucescens_AF163704
Schisandra_arisanensis_S106_TW
Schisandra_arisanensis_S105_TW
Schisandra_arisanensis_S111_TW
Schisandra_viridis_S16_HN
Schisandra_arisanensis_Sn_TW
Schisandra_viridis_SVB1_HENA
Schisandra_arisanensis_S108_TW
Schisandra_rubriflora_S6_YN
Schisandra_grandiflora_S81_YN
Schisandra_chinensis_S17_JL
Schisandra_chinensis_S13_BJ
Schisandra_chinensis_S18_BJ
Schisandra_chinensis_S001_BJ
Schisandra_chinensis_S025_BJ
Schisandra_rubriflora_S86_YN
Illicium_lanceolatum_AF163726_HB
Illicium_lanceolatum_isolate01
Illicium_lanceolatum_I34_ZJ
Illicium_burmanicum_I46_YN
Illicium_burmanicum_I048_YN
Illicium_henryi_I067_SX
Illicium_henryi_I041_SX
Illicium_henryi_I48_HN
Illicium_henryi_I066_SX
Illicium_henryi_I017_HB
Illicium_majus_I093_CQ
Illicium_majus_I091_SC
Illicium_majus_I092_CQ
Illicium_majus_AF163733_GD
Illicium_micranthum_I44_CQ
Illicium_micranthum_I044_YN
Illicium_micranthum_AF163727_HK
Illicium_fargesii_I43_CQ
Illicium_simonsii_I046_SC
Illicium_simonsii_I047_SC
Illicium_fargesii_I071_YN
Illicium_fargesii_I072_CQ
Illicium_fargesii_I021_GZ
Illicium_fargesii_I022_GZ
Illicium_simonsii_I42_SC
Illicium_verum_I068_GX
Illicium_verum_AF163724_GD
Illicium_verum_I038_YN
Illicium_verum_I39_SC
Illicium_ternstroemioides_I036_HN
Illicium_ternstroemioides_I064
Illicium_ternstroemioides_I47_HN
Illicium_ternstroemioides_EF138797
Illicium_arborescens_I015_TW
Illicium_arborescens_I089_TW
Illicium_arborescens_AF163723
Illicium_anisatum_I090_TW
Illicium_philippinense_I62_1_TW
Illicium_anisatum_AF163729_TW
Illicium_philippinense_I62_3_TW
Illicium_philippinense_I095_TW
Illicium_philippinense_I62_2_TW
Illicium_angustisepalum_AF163721_HK
Illicium_angustisepalum_I30_SZ
0.06

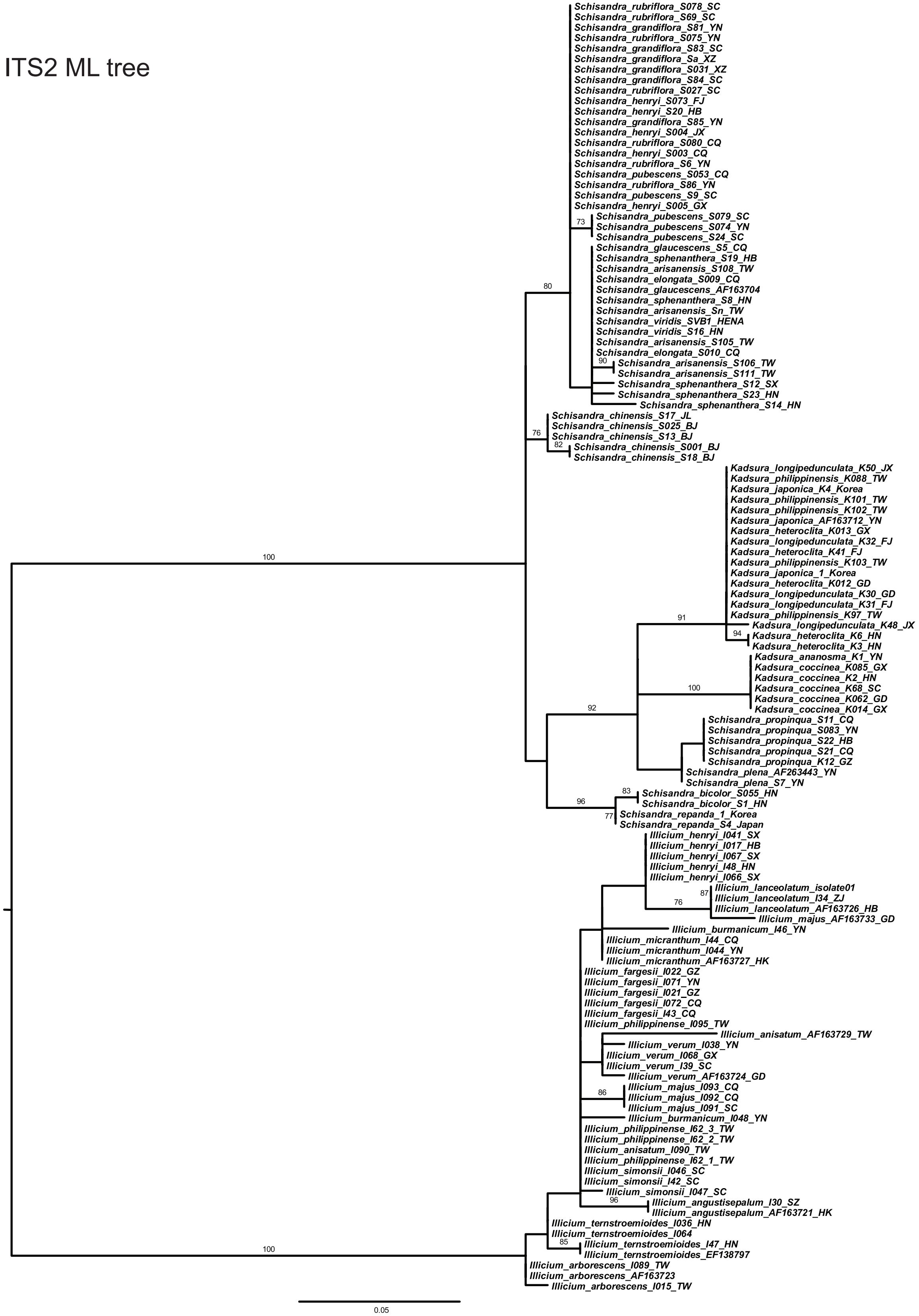

ITS2 ML tree
Schisandra_rubriflora_S078_SC
Schisandra_rubriflora_S69_SC
Schisandra_grandiflora_S81_YN
Schisandra_rubriflora_S075_YN
Schisandra_grandiflora_S83_SC
Schisandra_grandiflora_Sa_XZ
Schisandra_grandiflora_S031_XZ
Schisandra_grandiflora_S84_SC
Schisandra_rubriflora_S027_SC
Schisandra_henryi_S073_FJ
Schisandra_henryi_S20_HB
Schisandra_grandiflora_S85_YN
Schisandra_henryi_S004_JX
Schisandra_rubriflora_S080_CQ
Schisandra_henryi_S003_CQ
Schisandra_rubriflora_S6_YN
Schisandra_pubescens_S053_CQ
Schisandra_rubriflora_S86_YN
Schisandra_pubescens_S9_SC
Schisandra_henryi_S005_GX
73
Schisandra_pubescens_S079_SC
Schisandra_pubescens_S074_YN
Schisandra_pubescens_S24_SC
Schisandra_glaucescens_S5_CQ
Schisandra_sphenanthera_S19_HB
Schisandra_arisanensis_S108_TW
Schisandra_elongata_S009_CQ
80
Schisandra_glaucescens_AF163704
Schisandra_sphenanthera_S8_HN
Schisandra_arisanensis_Sn_TW
Schisandra_viridis_SVB1_HENA
Schisandra_viridis_S16_HN
Schisandra_arisanensis_S105_TW
Schisandra_elongata_S010_CQ
90
Schisandra_arisanensis_S106_TW
Schisandra_arisanensis_S111_TW
Schisandra_sphenanthera_S12_SX
Schisandra_sphenanthera_S23_HN
Schisandra_sphenanthera_S14_HN
Schisandra_chinensis_S17_JL
Schisandra_chinensis_S025_BJ
76
Schisandra_chinensis_S13_BJ
82
Schisandra_chinensis_S001_BJ
Schisandra_chinensis_S18_BJ
Kadsura_longipedunculata_K50_JX
Kadsura_philippinensis_K088_TW
Kadsura_japonica_K4_Korea
Kadsura_philippinensis_K101_TW
Kadsura_philippinensis_K102_TW
Kadsura_japonica_AF163712_YN
Kadsura_heteroclita_K013_GX
Kadsura_longipedunculata_K32_FJ
100
Kadsura_heteroclita_K41_FJ
Kadsura_philippinensis_K103_TW
Kadsura_japonica_1_Korea
Kadsura_heteroclita_K012_GD
Kadsura_longipedunculata_K30_GD
Kadsura_longipedunculata_K31_FJ
Kadsura_philippinensis_K97_TW
91
Kadsura_longipedunculata_K48_JX
94
Kadsura_heteroclita_K6_HN
Kadsura_heteroclita_K3_HN
Kadsura_ananosma_K1_YN
Kadsura_coccinea_K085_GX
Kadsura_coccinea_K2_HN
100
Kadsura_coccinea_K68_SC
Kadsura_coccinea_K062_GD
Kadsura_coccinea_K014_GX
92
Schisandra_propinqua_S11_CQ
Schisandra_propinqua_S083_YN
Schisandra_propinqua_S22_HB
Schisandra_propinqua_S21_CQ
Schisandra_propinqua_K12_GZ
Schisandra_plena_AF263443_YN
Schisandra_plena_S7_YN
83
Schisandra_bicolor_S055_HN
96
Schisandra_bicolor_S1_HN
77
Schisandra_repanda_1_Korea
Schisandra_repanda_S4_Japan
Illicium_henryi_I041_SX
Illicium_henryi_I017_HB
Illicium_henryi_I067_SX
Illicium_henryi_I48_HN
Illicium_henryi_I066_SX
87
Illicium_lanceolatum_isolate01
76
Illicium_lanceolatum_I34_ZJ
Illicium_lanceolatum_AF163726_HB
Illicium_majus_AF163733_GD
Illicium_burmanicum_I46_YN
Illicium_micranthum_I44_CQ
Illicium_micranthum_I044_YN
Illicium_micranthum_AF163727_HK
Illicium_fargesii_I022_GZ
Illicium_fargesii_I071_YN
Illicium_fargesii_I021_GZ
Illicium_fargesii_I072_CQ
Illicium_fargesii_I43_CQ
Illicium_philippinense_I095_TW
Illicium_anisatum_AF163729_TW
Illicium_verum_I038_YN
Illicium_verum_I068_GX
Illicium_verum_I39_SC
Illicium_verum_AF163724_GD
86
Illicium_majus_I093_CQ
Illicium_majus_I092_CQ
Illicium_majus_I091_SC
Illicium_burmanicum_I048_YN
Illicium_philippinense_I62_3_TW
Illicium_philippinense_I62_2_TW
Illicium_anisatum_I090_TW
Illicium_philippinense_I62_1_TW
Illicium_simonsii_I046_SC
Illicium_simonsii_I42_SC
Illicium_simonsii_I047_SC
96
Illicium_angustisepalum_I30_SZ
Illicium_angustisepalum_AF163721_HK
Illicium_ternstroemioides_I036_HN
Illicium_ternstroemioides_I064
85
Illicium_ternstroemioides_I47_HN
100
Illicium_ternstroemioides_EF138797
Illicium_arborescens_I089_TW
Illicium_arborescens_AF163723
Illicium_arborescens_I015_TW
0.05

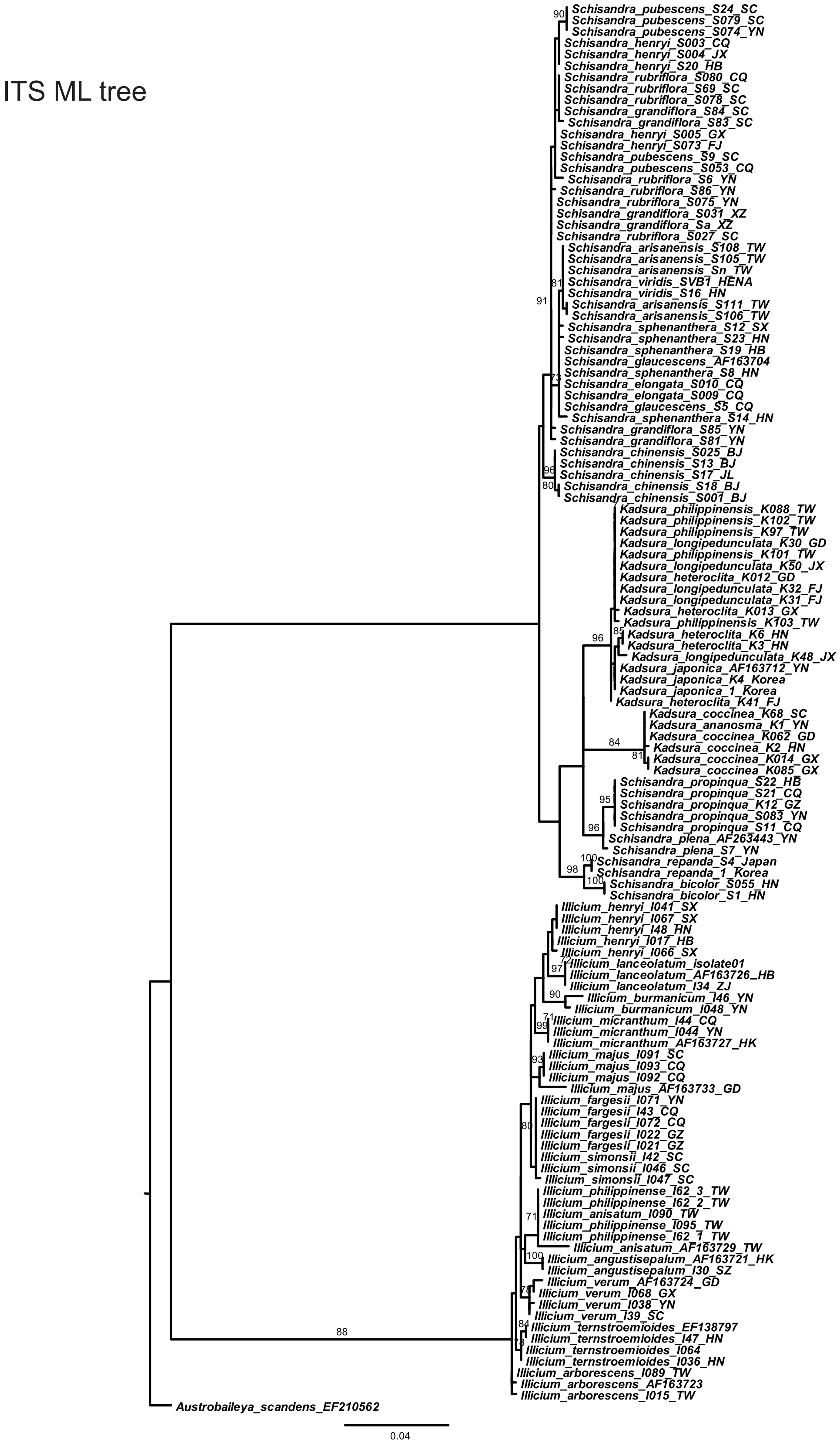
ITS ML tree
Schisandra_pubescens_S24_SC
Schisandra_pubescens_S079_SC
Schisandra_pubescens_S074_YN
Schisandra_henryi_S003_CQ
Schisandra_henryi_S004_JX
Schisandra_henryi_S20_HB
Schisandra_rubriflora_S080_CQ
Schisandra_rubriflora_S69_SC
Schisandra_rubriflora_S078_SC
Schisandra_grandiflora_S84_SC
Schisandra_grandiflora_S83_SC
Schisandra_henryi_S005_GX
Schisandra_henryi_S073_FJ
Schisandra_pubescens_S9_SC
Schisandra_pubescens_S053_CQ
Schisandra_rubriflora_S6_YN
Schisandra_rubriflora_S86_YN
Schisandra_rubriflora_S075_YN
Schisandra_grandiflora_S031_XZ
Schisandra_grandiflora_Sa_XZ
Schisandra_rubriflora_S027_SC
Schisandra_arisanensis_S108_TW
Schisandra_arisanensis_S105_TW
Schisandra_arisanensis_Sn_TW
Schisandra_viridis_SVB1_HENA
Schisandra_viridis_S16_HN
Schisandra_arisanensis_S111_TW
Schisandra_arisanensis_S106_TW
Schisandra_sphenanthera_S12_SX
Schisandra_sphenanthera_S23_HN
Schisandra_sphenanthera_S19_HB
Schisandra_glaucescens_AF163704
Schisandra_sphenanthera_S8_HN
Schisandra_elongata_S010_CQ
Schisandra_elongata_S009_CQ
Schisandra_glaucescens_S5_CQ
Schisandra_sphenanthera_S14_HN
Schisandra_grandiflora_S85_YN
Schisandra_grandiflora_S81_YN
Schisandra_chinensis_S025_BJ
Schisandra_chinensis_S13_BJ
Schisandra_chinensis_S17_JL
Schisandra_chinensis_S18_BJ
Schisandra_chinensis_S001_BJ
Kadsura_philippinensis_K088_TW
Kadsura_philippinensis_K102_TW
Kadsura_philippinensis_K97_TW
Kadsura_longipedunculata_K30_GD
Kadsura_philippinensis_K101_TW
Kadsura_longipedunculata_K50_JX
Kadsura_heteroclita_K012_GD
Kadsura_longipedunculata_K32_FJ
Kadsura_longipedunculata_K31_FJ
Kadsura_heteroclita_K013_GX
Kadsura_philippinensis_K103_TW
Kadsura_heteroclita_K6_HN
Kadsura_heteroclita_K3_HN
Kadsura_longipedunculata_K48_JX
Kadsura_japonica_AF163712_YN
Kadsura_japonica_K4_Korea
Kadsura_japonica_1_Korea
Kadsura_heteroclita_K41_FJ
Kadsura_coccinea_K68_SC
Kadsura_ananosma_K1_YN
Kadsura_coccinea_K062_GD
Kadsura_coccinea_K2_HN
Kadsura_coccinea_K014_GX
Kadsura_coccinea_K085_GX
Schisandra_propinqua_S22_HB
Schisandra_propinqua_S21_CQ
Schisandra_propinqua_K12_GZ
Schisandra_propinqua_S083_YN
Schisandra_propinqua_S11_CQ
Schisandra_plena_AF263443_YN
Schisandra_plena_S7_YN
Schisandra_repanda_S4_Japan
Schisandra_repanda_1_Korea
Schisandra_bicolor_S055_HN
Schisandra_bicolor_S1_HN
Illicium_henryi_I041_SX
Illicium_henryi_I067_SX
Illicium_henryi_I48_HN
Illicium_henryi_I017_HB
Illicium_henryi_I066_SX
Illicium_lanceolatum_isolate01
Illicium_lanceolatum_AF163726_HB
Illicium_lanceolatum_I34_ZJ
Illicium_burmanicum_I46_YN
Illicium_burmanicum_I048_YN
Illicium_micranthum_I44_CQ
Illicium_micranthum_I044_YN
Illicium_micranthum_AF163727_HK
Illicium_majus_I091_SC
Illicium_majus_I093_CQ
Illicium_majus_I092_CQ
Illicium_majus_AF163733_GD
Illicium_fargesii_I071_YN
Illicium_fargesii_I43_CQ
Illicium_fargesii_I072_CQ
Illicium_fargesii_I022_GZ
Illicium_fargesii_I021_GZ
Illicium_simonsii_I42_SC
Illicium_simonsii_I046_SC
Illicium_simonsii_I047_SC
Illicium_philippinense_I62_3_TW
Illicium_philippinense_I62_2_TW
Illicium_anisatum_I090_TW
Illicium_philippinense_I095_TW
Illicium_philippinense_I62_1_TW
Illicium_anisatum_AF163729_TW
Illicium_angustisepalum_AF163721_HK
Illicium_angustisepalum_I30_SZ
Illicium_verum_AF163724_GD
Illicium_verum_I068_GX
Illicium_verum_I038_YN
Illicium_verum_I39_SC
Illicium_ternstroemioides_EF138797
Illicium_ternstroemioides_I47_HN
Illicium_ternstroemioides_I064
Illicium_ternstroemioides_I036_HN
Illicium_arborescens_I089_TW
Illicium_arborescens_AF163723
Illicium_arborescens_I015_TW
Austrobaileya_scandens_EF210562
0.04

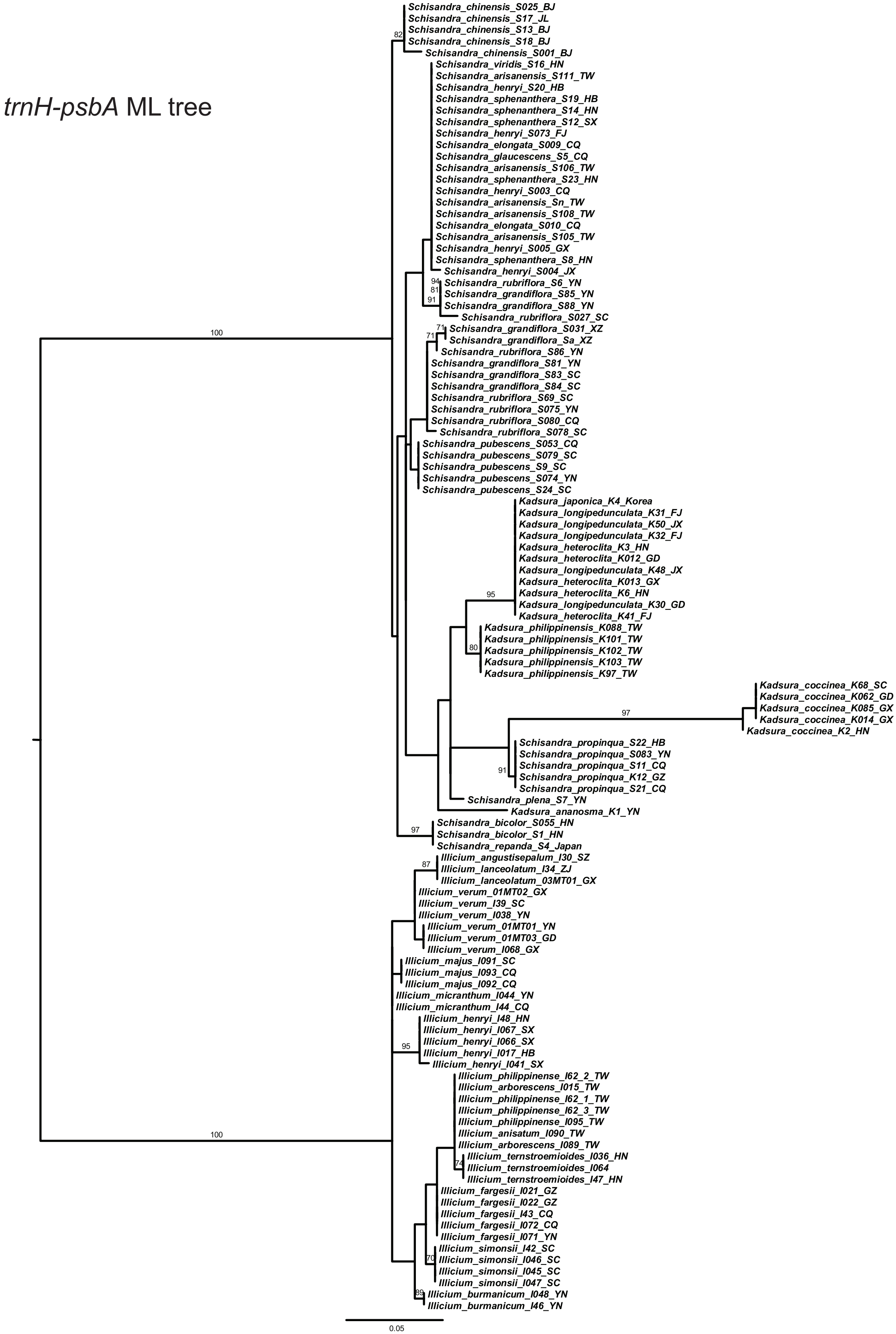
trnH-psbA ML tree
Schisandra_chinensis_S025_BJ
Schisandra_chinensis_S17_JL
Schisandra_chinensis_S13_BJ
Schisandra_chinensis_S18_BJ
Schisandra_chinensis_S001_BJ
Schisandra_viridis_S16_HN
Schisandra_arisanensis_S111_TW
Schisandra_henryi_S20_HB
Schisandra_sphenanthera_S19_HB
Schisandra_sphenanthera_S14_HN
Schisandra_sphenanthera_S12_SX
Schisandra_henryi_S073_FJ
Schisandra_elongata_S009_CQ
Schisandra_glaucescens_S5_CQ
Schisandra_arisanensis_S106_TW
Schisandra_sphenanthera_S23_HN
Schisandra_henryi_S003_CQ
Schisandra_arisanensis_Sn_TW
Schisandra_arisanensis_S108_TW
Schisandra_elongata_S010_CQ
Schisandra_arisanensis_S105_TW
Schisandra_henryi_S005_GX
Schisandra_sphenanthera_S8_HN
Schisandra_henryi_S004_JX
Schisandra_rubriflora_S6_YN
Schisandra_grandiflora_S85_YN
Schisandra_grandiflora_S88_YN
Schisandra_rubriflora_S027_SC
Schisandra_grandiflora_S031_XZ
Schisandra_grandiflora_Sa_XZ
Schisandra_rubriflora_S86_YN
Schisandra_grandiflora_S81_YN
Schisandra_grandiflora_S83_SC
Schisandra_grandiflora_S84_SC
Schisandra_rubriflora_S69_SC
Schisandra_rubriflora_S075_YN
Schisandra_rubriflora_S080_CQ
Schisandra_rubriflora_S078_SC
Schisandra_pubescens_S053_CQ
Schisandra_pubescens_S079_SC
Schisandra_pubescens_S9_SC
Schisandra_pubescens_S074_YN
Schisandra_pubescens_S24_SC
Kadsura_japonica_K4_Korea
Kadsura_longipedunculata_K31_FJ
Kadsura_longipedunculata_K50_JX
Kadsura_longipedunculata_K32_FJ
Kadsura_heteroclita_K3_HN
Kadsura_heteroclita_K012_GD
Kadsura_longipedunculata_K48_JX
Kadsura_heteroclita_K013_GX
Kadsura_heteroclita_K6_HN
Kadsura_longipedunculata_K30_GD
Kadsura_heteroclita_K41_FJ
Kadsura_philippinensis_K088_TW
Kadsura_philippinensis_K101_TW
Kadsura_philippinensis_K102_TW
Kadsura_philippinensis_K103_TW
Kadsura_philippinensis_K97_TW
Kadsura_coccinea_K68_SC
Kadsura_coccinea_K062_GD
Kadsura_coccinea_K085_GX
Kadsura_coccinea_K014_GX
Kadsura_coccinea_K2_HN
Schisandra_propinqua_S22_HB
Schisandra_propinqua_S083_YN
Schisandra_propinqua_S11_CQ
Schisandra_propinqua_K12_GZ
Schisandra_propinqua_S21_CQ
Schisandra_plena_S7_YN
Kadsura_ananosma_K1_YN
Schisandra_bicolor_S055_HN
Schisandra_bicolor_S1_HN
Schisandra_repanda_S4_Japan
Illicium_angustisepalum_I30_SZ
Illicium_lanceolatum_I34_ZJ
Illicium_lanceolatum_03MT01_GX
Illicium_verum_01MT02_GX
Illicium_verum_I39_SC
Illicium_verum_I038_YN
Illicium_verum_01MT01_YN
Illicium_verum_01MT03_GD
Illicium_verum_I068_GX
Illicium_majus_I091_SC
Illicium_majus_I093_CQ
Illicium_majus_I092_CQ
Illicium_micranthum_I044_YN
Illicium_micranthum_I44_CQ
Illicium_henryi_I48_HN
Illicium_henryi_I067_SX
Illicium_henryi_I066_SX
Illicium_henryi_I017_HB
Illicium_henryi_I041_SX
Illicium_philippinense_I62_2_TW
Illicium_arborescens_I015_TW
Illicium_philippinense_I62_1_TW
Illicium_philippinense_I62_3_TW
Illicium_philippinense_I095_TW
Illicium_anisatum_I090_TW
Illicium_arborescens_I089_TW
Illicium_ternstroemioides_I036_HN
Illicium_ternstroemioides_I064
Illicium_ternstroemioides_I47_HN
Illicium_fargesii_I021_GZ
Illicium_fargesii_I022_GZ
Illicium_fargesii_I43_CQ
Illicium_fargesii_I072_CQ
Illicium_fargesii_I071_YN
Illicium_simonsii_I42_SC
Illicium_simonsii_I046_SC
Illicium_simonsii_I045_SC
Illicium_simonsii_I047_SC
Illicium_burmanicum_I048_YN
Illicium_burmanicum_I46_YN
0.05

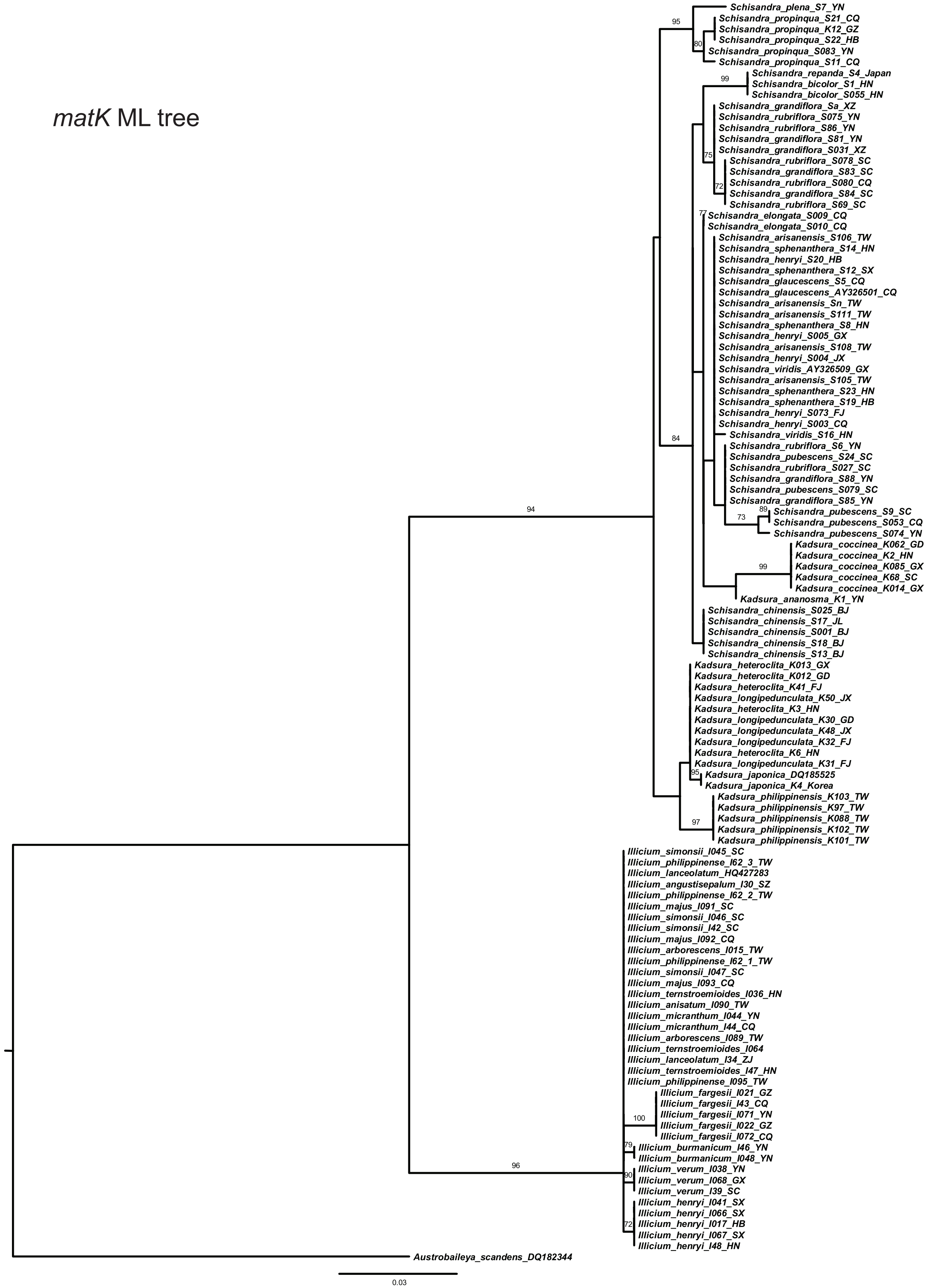

matK ML tree
Schisandra_plena_S7_YN
Schisandra_propinqua_S21_CQ
Schisandra_propinqua_K12_GZ
Schisandra_propinqua_S22_HB
Schisandra_propinqua_S083_YN
Schisandra_propinqua_S11_CQ
Schisandra_repanda_S4_Japan
Schisandra_bicolor_S1_HN
Schisandra_bicolor_S055_HN
Schisandra_grandiflora_Sa_XZ
Schisandra_rubriflora_S075_YN
Schisandra_rubriflora_S86_YN
Schisandra_grandiflora_S81_YN
Schisandra_grandiflora_S031_XZ
Schisandra_rubriflora_S078_SC
Schisandra_grandiflora_S83_SC
Schisandra_rubriflora_S080_CQ
Schisandra_grandiflora_S84_SC
Schisandra_rubriflora_S69_SC
Schisandra_elongata_S009_CQ
Schisandra_elongata_S010_CQ
Schisandra_arisanensis_S106_TW
Schisandra_sphenanthera_S14_HN
Schisandra_henryi_S20_HB
Schisandra_sphenanthera_S12_SX
Schisandra_glaucescens_S5_CQ
Schisandra_glaucescens_AY326501_CQ
Schisandra_arisanensis_Sn_TW
Schisandra_arisanensis_S111_TW
Schisandra_sphenanthera_S8_HN
Schisandra_henryi_S005_GX
Schisandra_arisanensis_S108_TW
Schisandra_henryi_S004_JX
Schisandra_viridis_AY326509_GX
Schisandra_arisanensis_S105_TW
Schisandra_sphenanthera_S23_HN
Schisandra_sphenanthera_S19_HB
Schisandra_henryi_S073_FJ
Schisandra_henryi_S003_CQ
Schisandra_viridis_S16_HN
Schisandra_rubriflora_S6_YN
Schisandra_pubescens_S24_SC
Schisandra_rubriflora_S027_SC
Schisandra_grandiflora_S88_YN
Schisandra_pubescens_S079_SC
Schisandra_grandiflora_S85_YN
Schisandra_pubescens_S9_SC
Schisandra_pubescens_S053_CQ
Schisandra_pubescens_S074_YN
Kadsura_coccinea_K062_GD
Kadsura_coccinea_K2_HN
Kadsura_coccinea_K085_GX
Kadsura_coccinea_K68_SC
Kadsura_coccinea_K014_GX
Kadsura_ananosma_K1_YN
Schisandra_chinensis_S025_BJ
Schisandra_chinensis_S17_JL
Schisandra_chinensis_S001_BJ
Schisandra_chinensis_S18_BJ
Schisandra_chinensis_S13_BJ
Kadsura_heteroclita_K013_GX
Kadsura_heteroclita_K012_GD
Kadsura_heteroclita_K41_FJ
Kadsura_longipedunculata_K50_JX
Kadsura_heteroclita_K3_HN
Kadsura_longipedunculata_K30_GD
Kadsura_longipedunculata_K48_JX
Kadsura_longipedunculata_K32_FJ
Kadsura_heteroclita_K6_HN
Kadsura_longipedunculata_K31_FJ
Kadsura_japonica_DQ185525
Kadsura_japonica_K4_Korea
Kadsura_philippinensis_K103_TW
Kadsura_philippinensis_K97_TW
Kadsura_philippinensis_K088_TW
Kadsura_philippinensis_K102_TW
Kadsura_philippinensis_K101_TW
Illicium_simonsii_I045_SC
Illicium_philippinense_I62_3_TW
Illicium_lanceolatum_HQ427283
Illicium_angustisepalum_I30_SZ
Illicium_philippinense_I62_2_TW
Illicium_majus_I091_SC
Illicium_simonsii_I046_SC
Illicium_simonsii_I42_SC
Illicium_majus_I092_CQ
Illicium_arborescens_I015_TW
Illicium_philippinense_I62_1_TW
Illicium_simonsii_I047_SC
Illicium_majus_I093_CQ
Illicium_ternstroemioides_I036_HN
Illicium_anisatum_I090_TW
Illicium_micranthum_I044_YN
Illicium_micranthum_I44_CQ
Illicium_arborescens_I089_TW
Illicium_ternstroemioides_I064
Illicium_lanceolatum_I34_ZJ
Illicium_ternstroemioides_I47_HN
Illicium_philippinense_I095_TW
Illicium_fargesii_I021_GZ
Illicium_fargesii_I43_CQ
Illicium_fargesii_I071_YN
Illicium_fargesii_I022_GZ
Illicium_fargesii_I072_CQ
Illicium_burmanicum_I46_YN
Illicium_burmanicum_I048_YN
Illicium_verum_I038_YN
Illicium_verum_I068_GX
Illicium_verum_I39_SC
Illicium_henryi_I041_SX
Illicium_henryi_I066_SX
Illicium_henryi_I017_HB
Illicium_henryi_I067_SX
Illicium_henryi_I48_HN
Austrobaileya_scandens_DQ182344
95
80
99
75
72
77
84
94
73
89
99
95
97
100
79
90
72
96
0.03

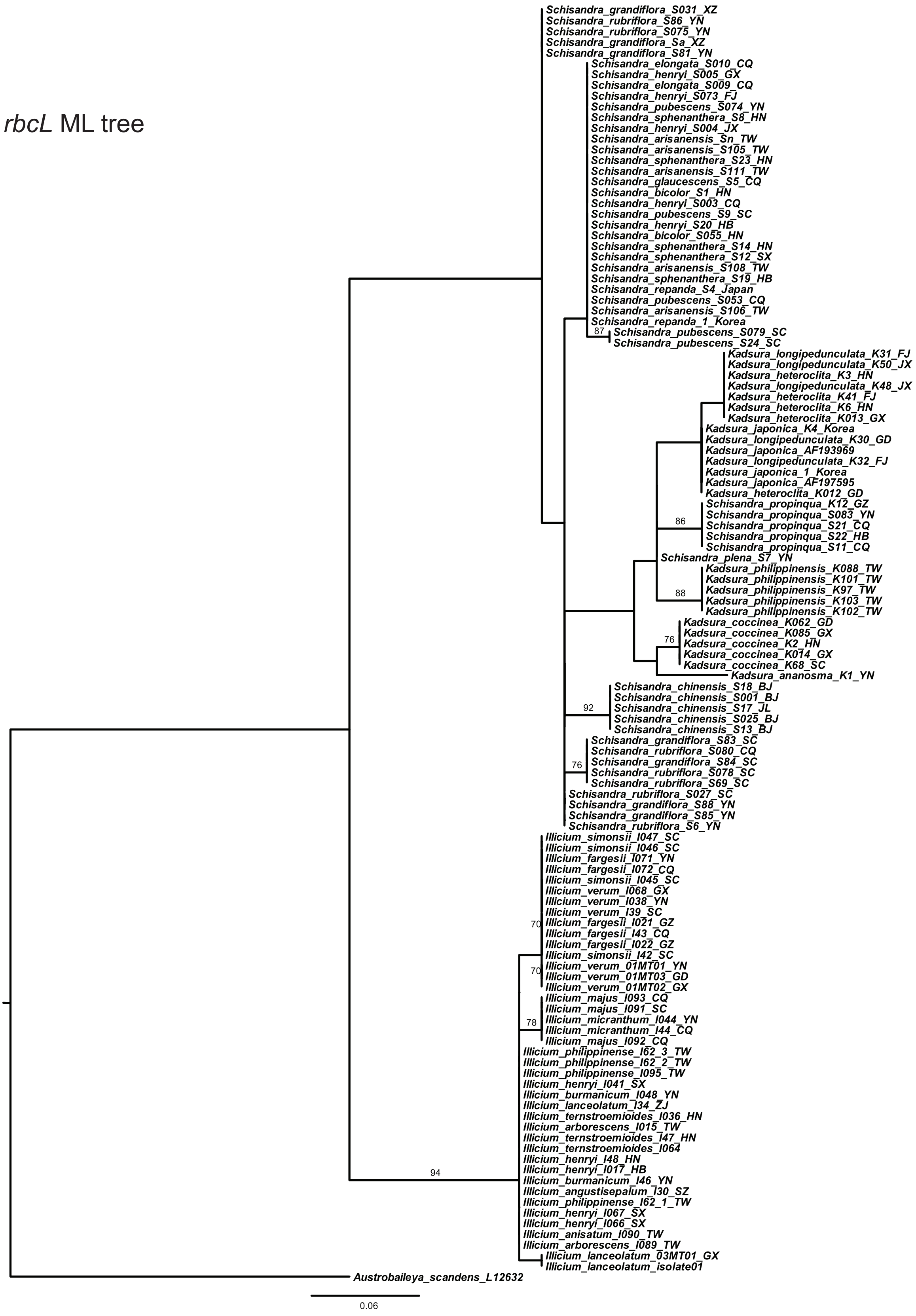
rbcL ML tree
Schisandra_grandiflora_S031_XZ
Schisandra_rubriflora_S86_YN
Schisandra_rubriflora_S075_YN
Schisandra_grandiflora_Sa_XZ
Schisandra_grandiflora_S81_YN
Schisandra_elongata_S010_CQ
Schisandra_henryi_S005_GX
Schisandra_elongata_S009_CQ
Schisandra_henryi_S073_FJ
Schisandra_pubescens_S074_YN
Schisandra_sphenanthera_S8_HN
Schisandra_henryi_S004_JX
Schisandra_arisanensis_Sn_TW
Schisandra_arisanensis_S105_TW
Schisandra_sphenanthera_S23_HN
Schisandra_arisanensis_S111_TW
Schisandra_glaucescens_S5_CQ
Schisandra_bicolor_S1_HN
Schisandra_henryi_S003_CQ
Schisandra_pubescens_S9_SC
Schisandra_henryi_S20_HB
Schisandra_bicolor_S055_HN
Schisandra_sphenanthera_S14_HN
Schisandra_sphenanthera_S12_SX
Schisandra_arisanensis_S108_TW
Schisandra_sphenanthera_S19_HB
Schisandra_repanda_S4_Japan
Schisandra_pubescens_S053_CQ
Schisandra_arisanensis_S106_TW
Schisandra_repanda_1_Korea
87
Schisandra_pubescens_S079_SC
Schisandra_pubescens_S24_SC
Kadsura_longipedunculata_K31_FJ
Kadsura_longipedunculata_K50_JX
Kadsura_heteroclita_K3_HN
Kadsura_longipedunculata_K48_JX
Kadsura_heteroclita_K41_FJ
Kadsura_heteroclita_K6_HN
Kadsura_heteroclita_K013_GX
Kadsura_japonica_K4_Korea
Kadsura_longipedunculata_K30_GD
Kadsura_japonica_AF193969
Kadsura_longipedunculata_K32_FJ
Kadsura_japonica_1_Korea
Kadsura_japonica_AF197595
Kadsura_heteroclita_K012_GD
Schisandra_propinqua_K12_GZ
Schisandra_propinqua_S083_YN
86
Schisandra_propinqua_S21_CQ
Schisandra_propinqua_S22_HB
Schisandra_propinqua_S11_CQ
Schisandra_plena_S7_YN
Kadsura_philippinensis_K088_TW
Kadsura_philippinensis_K101_TW
Kadsura_philippinensis_K97_TW
88
Kadsura_philippinensis_K103_TW
Kadsura_philippinensis_K102_TW
Kadsura_coccinea_K062_GD
Kadsura_coccinea_K085_GX
76
Kadsura_coccinea_K2_HN
Kadsura_coccinea_K014_GX
Kadsura_coccinea_K68_SC
Kadsura_ananosma_K1_YN
Schisandra_chinensis_S18_BJ
Schisandra_chinensis_S001_BJ
92
Schisandra_chinensis_S17_JL
Schisandra_chinensis_S025_BJ
Schisandra_chinensis_S13_BJ
Schisandra_grandiflora_S83_SC
Schisandra_rubriflora_S080_CQ
76
Schisandra_grandiflora_S84_SC
Schisandra_rubriflora_S078_SC
Schisandra_rubriflora_S69_SC
Schisandra_rubriflora_S027_SC
Schisandra_grandiflora_S88_YN
Schisandra_grandiflora_S85_YN
Schisandra_rubriflora_S6_YN
Illicium_simonsii_I047_SC
Illicium_simonsii_I046_SC
Illicium_fargesii_I071_YN
Illicium_fargesii_I072_CQ
Illicium_simonsii_I045_SC
Illicium_verum_I068_GX
Illicium_verum_I038_YN
Illicium_verum_I39_SC
70
Illicium_fargesii_I021_GZ
Illicium_fargesii_I43_CQ
Illicium_fargesii_I022_GZ
Illicium_simonsii_I42_SC
70
Illicium_verum_01MT01_YN
Illicium_verum_01MT03_GD
Illicium_verum_01MT02_GX
Illicium_majus_I093_CQ
Illicium_majus_I091_SC
78
Illicium_micranthum_I044_YN
Illicium_micranthum_I44_CQ
Illicium_majus_I092_CQ
Illicium_philippinense_I62_3_TW
Illicium_philippinense_I62_2_TW
Illicium_philippinense_I095_TW
Illicium_henryi_I041_SX
Illicium_burmanicum_I048_YN
Illicium_lanceolatum_I34_ZJ
Illicium_ternstroemioides_I036_HN
Illicium_arborescens_I015_TW
Illicium_ternstroemioides_I47_HN
Illicium_ternstroemioides_I064
Illicium_henryi_I48_HN
Illicium_henryi_I017_HB
94
Illicium_burmanicum_I46_YN
Illicium_angustisepalum_I30_SZ
Illicium_philippinense_I62_1_TW
Illicium_henryi_I067_SX
Illicium_henryi_I066_SX
Illicium_anisatum_I090_TW
Illicium_arborescens_I089_TW
Illicium_lanceolatum_03MT01_GX
Illicium_lanceolatum_isolate01
Austrobaileya_scandens_L12632
0.06

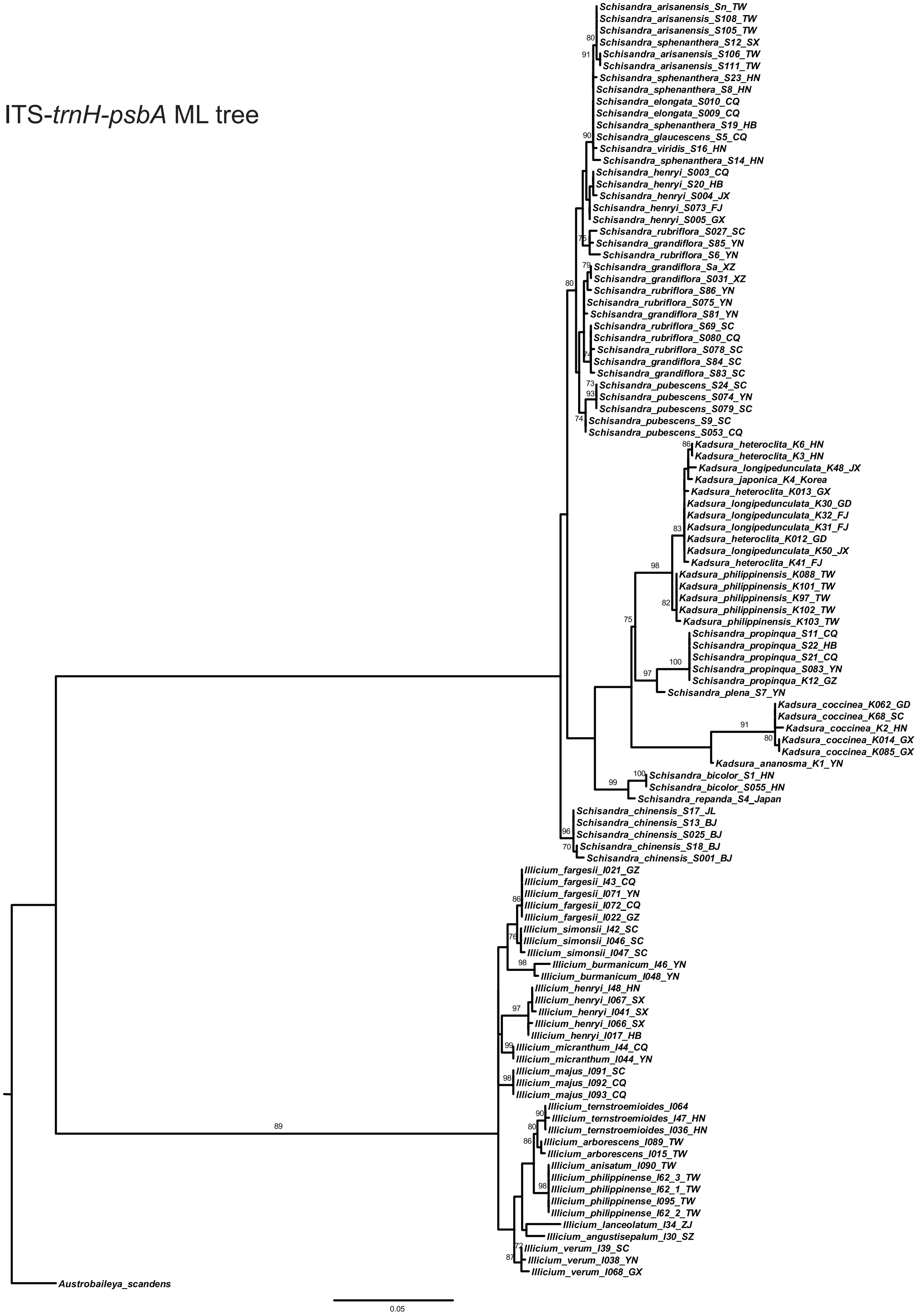
ITS-trnH-psbA ML tree
Schisandra_arisanensis_Sn_TW
Schisandra_arisanensis_S108_TW
Schisandra_arisanensis_S105_TW
Schisandra_sphenanthera_S12_SX
Schisandra_arisanensis_S106_TW
Schisandra_arisanensis_S111_TW
Schisandra_sphenanthera_S23_HN
Schisandra_sphenanthera_S8_HN
Schisandra_elongata_S010_CQ
Schisandra_elongata_S009_CQ
Schisandra_sphenanthera_S19_HB
Schisandra_glaucescens_S5_CQ
Schisandra_viridis_S16_HN
Schisandra_sphenanthera_S14_HN
Schisandra_henryi_S003_CQ
Schisandra_henryi_S20_HB
Schisandra_henryi_S004_JX
Schisandra_henryi_S073_FJ
Schisandra_henryi_S005_GX
Schisandra_rubriflora_S027_SC
Schisandra_grandiflora_S85_YN
Schisandra_rubriflora_S6_YN
Schisandra_grandiflora_Sa_XZ
Schisandra_grandiflora_S031_XZ
Schisandra_rubriflora_S86_YN
Schisandra_rubriflora_S075_YN
Schisandra_grandiflora_S81_YN
Schisandra_rubriflora_S69_SC
Schisandra_rubriflora_S080_CQ
Schisandra_rubriflora_S078_SC
Schisandra_grandiflora_S84_SC
Schisandra_grandiflora_S83_SC
Schisandra_pubescens_S24_SC
Schisandra_pubescens_S074_YN
Schisandra_pubescens_S079_SC
Schisandra_pubescens_S9_SC
Schisandra_pubescens_S053_CQ
Kadsura_heteroclita_K6_HN
Kadsura_heteroclita_K3_HN
Kadsura_longipedunculata_K48_JX
Kadsura_japonica_K4_Korea
Kadsura_heteroclita_K013_GX
Kadsura_longipedunculata_K30_GD
Kadsura_longipedunculata_K32_FJ
Kadsura_longipedunculata_K31_FJ
Kadsura_heteroclita_K012_GD
Kadsura_longipedunculata_K50_JX
Kadsura_heteroclita_K41_FJ
Kadsura_philippinensis_K088_TW
Kadsura_philippinensis_K101_TW
Kadsura_philippinensis_K97_TW
Kadsura_philippinensis_K102_TW
Kadsura_philippinensis_K103_TW
Schisandra_propinqua_S11_CQ
Schisandra_propinqua_S22_HB
Schisandra_propinqua_S21_CQ
Schisandra_propinqua_S083_YN
Schisandra_propinqua_K12_GZ
Schisandra_plena_S7_YN
Kadsura_coccinea_K062_GD
Kadsura_coccinea_K68_SC
Kadsura_coccinea_K2_HN
Kadsura_coccinea_K014_GX
Kadsura_coccinea_K085_GX
Kadsura_ananosma_K1_YN
Schisandra_bicolor_S1_HN
Schisandra_bicolor_S055_HN
Schisandra_repanda_S4_Japan
Schisandra_chinensis_S17_JL
Schisandra_chinensis_S13_BJ
Schisandra_chinensis_S025_BJ
Schisandra_chinensis_S18_BJ
Schisandra_chinensis_S001_BJ
Illicium_fargesii_I021_GZ
Illicium_fargesii_I43_CQ
Illicium_fargesii_I071_YN
Illicium_fargesii_I072_CQ
Illicium_fargesii_I022_GZ
Illicium_simonsii_I42_SC
Illicium_simonsii_I046_SC
Illicium_simonsii_I047_SC
Illicium_burmanicum_I46_YN
Illicium_burmanicum_I048_YN
Illicium_henryi_I48_HN
Illicium_henryi_I067_SX
Illicium_henryi_I041_SX
Illicium_henryi_I066_SX
Illicium_henryi_I017_HB
Illicium_micranthum_I44_CQ
Illicium_micranthum_I044_YN
Illicium_majus_I091_SC
Illicium_majus_I092_CQ
Illicium_majus_I093_CQ
Illicium_ternstroemioides_I064
Illicium_ternstroemioides_I47_HN
Illicium_ternstroemioides_I036_HN
Illicium_arborescens_I089_TW
Illicium_arborescens_I015_TW
Illicium_anisatum_I090_TW
Illicium_philippinense_I62_3_TW
Illicium_philippinense_I62_1_TW
Illicium_philippinense_I095_TW
Illicium_philippinense_I62_2_TW
Illicium_lanceolatum_I34_ZJ
Illicium_angustisepalum_I30_SZ
Illicium_verum_I39_SC
Illicium_verum_I038_YN
Illicium_verum_I068_GX
Austrobaileya_scandens
0.05

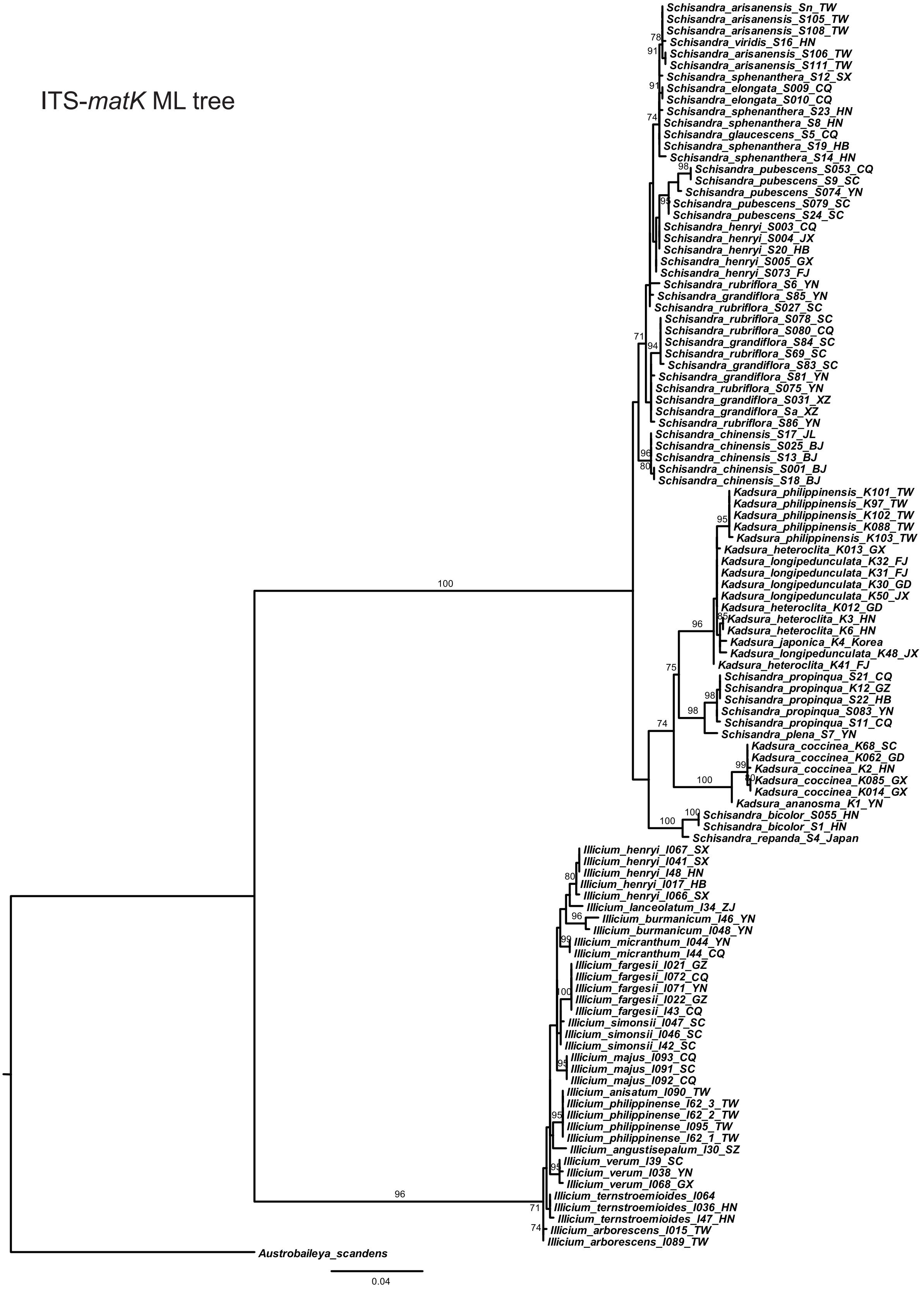
ITS-matK ML tree
Schisandra_arisanensis_Sn_TW
Schisandra_arisanensis_S105_TW
Schisandra_arisanensis_S108_TW
Schisandra_viridis_S16_HN
Schisandra_arisanensis_S106_TW
Schisandra_arisanensis_S111_TW
Schisandra_sphenanthera_S12_SX
Schisandra_elongata_S009_CQ
Schisandra_elongata_S010_CQ
Schisandra_sphenanthera_S23_HN
Schisandra_sphenanthera_S8_HN
Schisandra_glaucescens_S5_CQ
Schisandra_sphenanthera_S19_HB
Schisandra_sphenanthera_S14_HN
Schisandra_pubescens_S053_CQ
Schisandra_pubescens_S9_SC
Schisandra_pubescens_S074_YN
Schisandra_pubescens_S079_SC
Schisandra_pubescens_S24_SC
Schisandra_henryi_S003_CQ
Schisandra_henryi_S004_JX
Schisandra_henryi_S20_HB
Schisandra_henryi_S005_GX
Schisandra_henryi_S073_FJ
Schisandra_rubriflora_S6_YN
Schisandra_grandiflora_S85_YN
Schisandra_rubriflora_S027_SC
Schisandra_rubriflora_S078_SC
Schisandra_rubriflora_S080_CQ
Schisandra_grandiflora_S84_SC
Schisandra_rubriflora_S69_SC
Schisandra_grandiflora_S83_SC
Schisandra_grandiflora_S81_YN
Schisandra_rubriflora_S075_YN
Schisandra_grandiflora_S031_XZ
Schisandra_grandiflora_Sa_XZ
Schisandra_rubriflora_S86_YN
Schisandra_chinensis_S17_JL
Schisandra_chinensis_S025_BJ
Schisandra_chinensis_S13_BJ
Schisandra_chinensis_S001_BJ
Schisandra_chinensis_S18_BJ
Kadsura_philippinensis_K101_TW
Kadsura_philippinensis_K97_TW
Kadsura_philippinensis_K102_TW
Kadsura_philippinensis_K088_TW
Kadsura_philippinensis_K103_TW
Kadsura_heteroclita_K013_GX
Kadsura_longipedunculata_K32_FJ
Kadsura_longipedunculata_K31_FJ
Kadsura_longipedunculata_K30_GD
Kadsura_longipedunculata_K50_JX
Kadsura_heteroclita_K012_GD
Kadsura_heteroclita_K3_HN
Kadsura_heteroclita_K6_HN
Kadsura_japonica_K4_Korea
Kadsura_longipedunculata_K48_JX
Kadsura_heteroclita_K41_FJ
Schisandra_propinqua_S21_CQ
Schisandra_propinqua_K12_GZ
Schisandra_propinqua_S22_HB
Schisandra_propinqua_S083_YN
Schisandra_propinqua_S11_CQ
Schisandra_plena_S7_YN
Kadsura_coccinea_K68_SC
Kadsura_coccinea_K062_GD
Kadsura_coccinea_K2_HN
Kadsura_coccinea_K085_GX
Kadsura_coccinea_K014_GX
Kadsura_ananosma_K1_YN
Schisandra_bicolor_S055_HN
Schisandra_bicolor_S1_HN
Schisandra_repanda_S4_Japan
Illicium_henryi_I067_SX
Illicium_henryi_I041_SX
Illicium_henryi_I48_HN
Illicium_henryi_I017_HB
Illicium_henryi_I066_SX
Illicium_lanceolatum_I34_ZJ
Illicium_burmanicum_I46_YN
Illicium_burmanicum_I048_YN
Illicium_micranthum_I044_YN
Illicium_micranthum_I44_CQ
Illicium_fargesii_I021_GZ
Illicium_fargesii_I072_CQ
Illicium_fargesii_I071_YN
Illicium_fargesii_I022_GZ
Illicium_fargesii_I43_CQ
Illicium_simonsii_I047_SC
Illicium_simonsii_I046_SC
Illicium_simonsii_I42_SC
Illicium_majus_I093_CQ
Illicium_majus_I091_SC
Illicium_majus_I092_CQ
Illicium_anisatum_I090_TW
Illicium_philippinense_I62_3_TW
Illicium_philippinense_I62_2_TW
Illicium_philippinense_I095_TW
Illicium_philippinense_I62_1_TW
Illicium_angustisepalum_I30_SZ
Illicium_verum_I39_SC
Illicium_verum_I038_YN
Illicium_verum_I068_GX
Illicium_ternstroemioides_I064
Illicium_ternstroemioides_I036_HN
Illicium_ternstroemioides_I47_HN
Illicium_arborescens_I015_TW
Illicium_arborescens_I089_TW
Austrobaileya_scandens
0.04

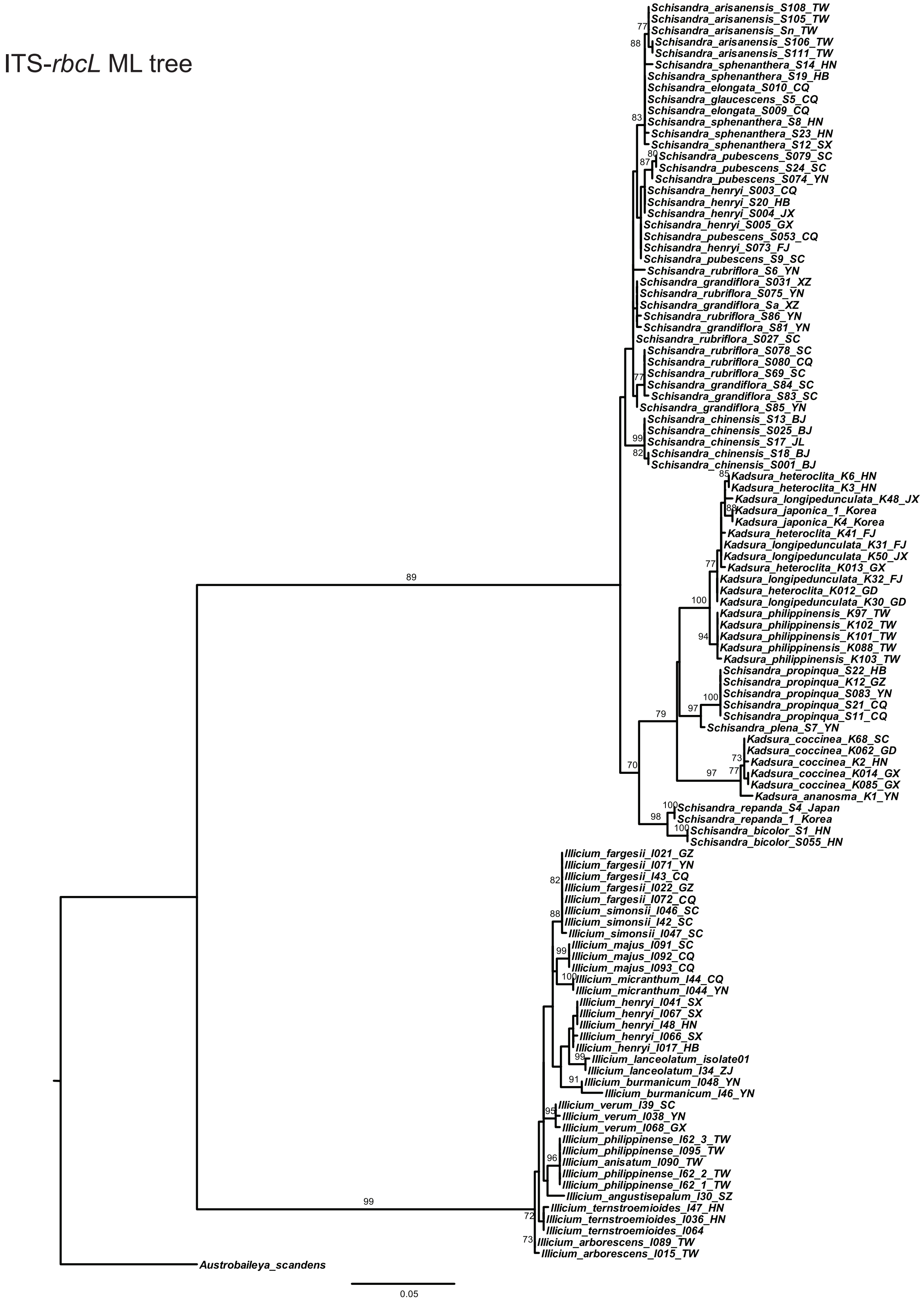
ITS-rbcL ML tree
Schisandra_arisanensis_S108_TW
Schisandra_arisanensis_S105_TW
Schisandra_arisanensis_Sn_TW
Schisandra_arisanensis_S106_TW
Schisandra_arisanensis_S111_TW
Schisandra_sphenanthera_S14_HN
Schisandra_sphenanthera_S19_HB
Schisandra_elongata_S010_CQ
Schisandra_glaucescens_S5_CQ
Schisandra_elongata_S009_CQ
Schisandra_sphenanthera_S8_HN
Schisandra_sphenanthera_S23_HN
Schisandra_sphenanthera_S12_SX
Schisandra_pubescens_S079_SC
Schisandra_pubescens_S24_SC
Schisandra_pubescens_S074_YN
Schisandra_henryi_S003_CQ
Schisandra_henryi_S20_HB
Schisandra_henryi_S004_JX
Schisandra_henryi_S005_GX
Schisandra_pubescens_S053_CQ
Schisandra_henryi_S073_FJ
Schisandra_pubescens_S9_SC
Schisandra_rubriflora_S6_YN
Schisandra_grandiflora_S031_XZ
Schisandra_rubriflora_S075_YN
Schisandra_grandiflora_Sa_XZ
Schisandra_rubriflora_S86_YN
Schisandra_grandiflora_S81_YN
Schisandra_rubriflora_S027_SC
Schisandra_rubriflora_S078_SC
Schisandra_rubriflora_S080_CQ
Schisandra_rubriflora_S69_SC
Schisandra_grandiflora_S84_SC
Schisandra_grandiflora_S83_SC
Schisandra_grandiflora_S85_YN
Schisandra_chinensis_S13_BJ
Schisandra_chinensis_S025_BJ
Schisandra_chinensis_S17_JL
Schisandra_chinensis_S18_BJ
Schisandra_chinensis_S001_BJ
Kadsura_heteroclita_K6_HN
Kadsura_heteroclita_K3_HN
Kadsura_longipedunculata_K48_JX
Kadsura_japonica_1_Korea
Kadsura_japonica_K4_Korea
Kadsura_heteroclita_K41_FJ
Kadsura_longipedunculata_K31_FJ
Kadsura_longipedunculata_K50_JX
Kadsura_heteroclita_K013_GX
Kadsura_longipedunculata_K32_FJ
Kadsura_heteroclita_K012_GD
Kadsura_longipedunculata_K30_GD
Kadsura_philippinensis_K97_TW
Kadsura_philippinensis_K102_TW
Kadsura_philippinensis_K101_TW
Kadsura_philippinensis_K088_TW
Kadsura_philippinensis_K103_TW
Schisandra_propinqua_S22_HB
Schisandra_propinqua_K12_GZ
Schisandra_propinqua_S083_YN
Schisandra_propinqua_S21_CQ
Schisandra_propinqua_S11_CQ
Schisandra_plena_S7_YN
Kadsura_coccinea_K68_SC
Kadsura_coccinea_K062_GD
Kadsura_coccinea_K2_HN
Kadsura_coccinea_K014_GX
Kadsura_coccinea_K085_GX
Kadsura_ananosma_K1_YN
Schisandra_repanda_S4_Japan
Schisandra_repanda_1_Korea
Schisandra_bicolor_S1_HN
Schisandra_bicolor_S055_HN
Illicium_fargesii_I021_GZ
Illicium_fargesii_I071_YN
Illicium_fargesii_I43_CQ
Illicium_fargesii_I022_GZ
Illicium_fargesii_I072_CQ
Illicium_simonsii_I046_SC
Illicium_simonsii_I42_SC
Illicium_simonsii_I047_SC
Illicium_majus_I091_SC
Illicium_majus_I092_CQ
Illicium_majus_I093_CQ
Illicium_micranthum_I44_CQ
Illicium_micranthum_I044_YN
Illicium_henryi_I041_SX
Illicium_henryi_I067_SX
Illicium_henryi_I48_HN
Illicium_henryi_I066_SX
Illicium_henryi_I017_HB
Illicium_lanceolatum_isolate01
Illicium_lanceolatum_I34_ZJ
Illicium_burmanicum_I048_YN
Illicium_burmanicum_I46_YN
Illicium_verum_I39_SC
Illicium_verum_I038_YN
Illicium_verum_I068_GX
Illicium_philippinense_I62_3_TW
Illicium_philippinense_I095_TW
Illicium_anisatum_I090_TW
Illicium_philippinense_I62_2_TW
Illicium_philippinense_I62_1_TW
Illicium_angustisepalum_I30_SZ
Illicium_ternstroemioides_I47_HN
Illicium_ternstroemioides_I036_HN
Illicium_ternstroemioides_I064
Illicium_arborescens_I089_TW
Illicium_arborescens_I015_TW
Austrobaileya_scandens
0.05

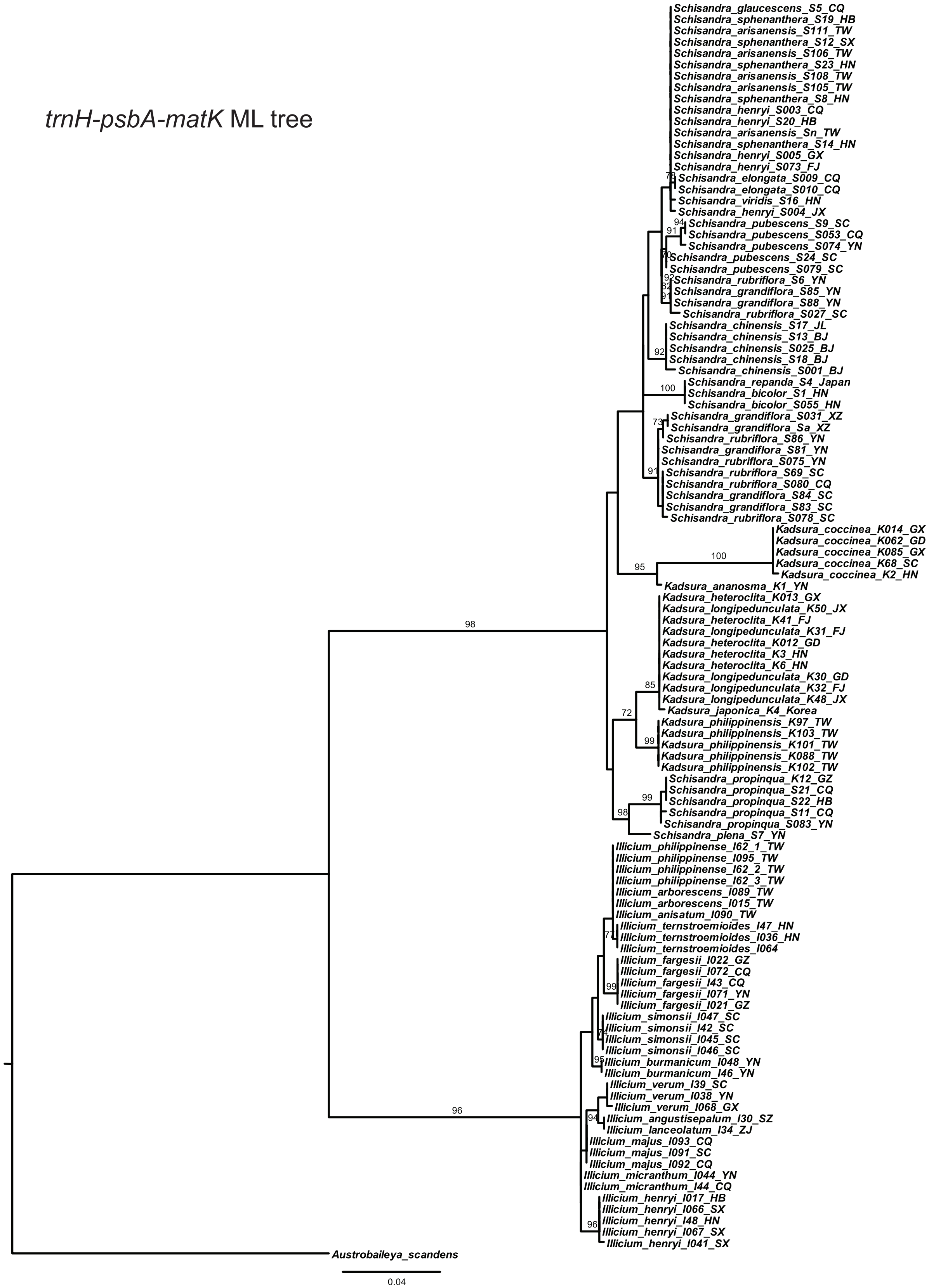

trnH-psbA-matK ML tree
Schisandra_glaucescens_S5_CQ
Schisandra_sphenanthera_S19_HB
Schisandra_arisanensis_S111_TW
Schisandra_sphenanthera_S12_SX
Schisandra_arisanensis_S106_TW
Schisandra_sphenanthera_S23_HN
Schisandra_arisanensis_S108_TW
Schisandra_arisanensis_S105_TW
Schisandra_sphenanthera_S8_HN
Schisandra_henryi_S003_CQ
Schisandra_henryi_S20_HB
Schisandra_arisanensis_Sn_TW
Schisandra_sphenanthera_S14_HN
Schisandra_henryi_S005_GX
Schisandra_henryi_S073_FJ
Schisandra_elongata_S009_CQ
Schisandra_elongata_S010_CQ
Schisandra_viridis_S16_HN
Schisandra_henryi_S004_JX
Schisandra_pubescens_S9_SC
Schisandra_pubescens_S053_CQ
Schisandra_pubescens_S074_YN
Schisandra_pubescens_S24_SC
Schisandra_pubescens_S079_SC
Schisandra_rubriflora_S6_YN
Schisandra_grandiflora_S85_YN
Schisandra_grandiflora_S88_YN
Schisandra_rubriflora_S027_SC
Schisandra_chinensis_S17_JL
Schisandra_chinensis_S13_BJ
Schisandra_chinensis_S025_BJ
Schisandra_chinensis_S18_BJ
Schisandra_chinensis_S001_BJ
Schisandra_repanda_S4_Japan
Schisandra_bicolor_S1_HN
Schisandra_bicolor_S055_HN
Schisandra_grandiflora_S031_XZ
Schisandra_grandiflora_Sa_XZ
Schisandra_rubriflora_S86_YN
Schisandra_grandiflora_S81_YN
Schisandra_rubriflora_S075_YN
Schisandra_rubriflora_S69_SC
Schisandra_rubriflora_S080_CQ
Schisandra_grandiflora_S84_SC
Schisandra_grandiflora_S83_SC
Schisandra_rubriflora_S078_SC
Kadsura_coccinea_K014_GX
Kadsura_coccinea_K062_GD
Kadsura_coccinea_K085_GX
Kadsura_coccinea_K68_SC
Kadsura_coccinea_K2_HN
Kadsura_ananosma_K1_YN
Kadsura_heteroclita_K013_GX
Kadsura_longipedunculata_K50_JX
Kadsura_heteroclita_K41_FJ
Kadsura_longipedunculata_K31_FJ
Kadsura_heteroclita_K012_GD
Kadsura_heteroclita_K3_HN
Kadsura_heteroclita_K6_HN
Kadsura_longipedunculata_K30_GD
Kadsura_longipedunculata_K32_FJ
Kadsura_longipedunculata_K48_JX
Kadsura_japonica_K4_Korea
Kadsura_philippinensis_K97_TW
Kadsura_philippinensis_K103_TW
Kadsura_philippinensis_K101_TW
Kadsura_philippinensis_K088_TW
Kadsura_philippinensis_K102_TW
Schisandra_propinqua_K12_GZ
Schisandra_propinqua_S21_CQ
Schisandra_propinqua_S22_HB
Schisandra_propinqua_S11_CQ
Schisandra_propinqua_S083_YN
Schisandra_plena_S7_YN
Illicium_philippinense_I62_1_TW
Illicium_philippinense_I095_TW
Illicium_philippinense_I62_2_TW
Illicium_philippinense_I62_3_TW
Illicium_arborescens_I089_TW
Illicium_arborescens_I015_TW
Illicium_anisatum_I090_TW
Illicium_ternstroemioides_I47_HN
Illicium_ternstroemioides_I036_HN
Illicium_ternstroemioides_I064
Illicium_fargesii_I022_GZ
Illicium_fargesii_I072_CQ
Illicium_fargesii_I43_CQ
Illicium_fargesii_I071_YN
Illicium_fargesii_I021_GZ
Illicium_simonsii_I047_SC
Illicium_simonsii_I42_SC
Illicium_simonsii_I045_SC
Illicium_simonsii_I046_SC
Illicium_burmanicum_I048_YN
Illicium_burmanicum_I46_YN
Illicium_verum_I39_SC
Illicium_verum_I038_YN
Illicium_verum_I068_GX
Illicium_angustisepalum_I30_SZ
Illicium_lanceolatum_I34_ZJ
Illicium_majus_I093_CQ
Illicium_majus_I091_SC
Illicium_majus_I092_CQ
Illicium_micranthum_I044_YN
Illicium_micranthum_I44_CQ
Illicium_henryi_I017_HB
Illicium_henryi_I066_SX
Illicium_henryi_I48_HN
Illicium_henryi_I067_SX
Illicium_henryi_I041_SX
Austrobaileya_scandens
98
96
100
95
100
92
91
94
72
85
99
99
98
77
99
74
95
94
96
0.04

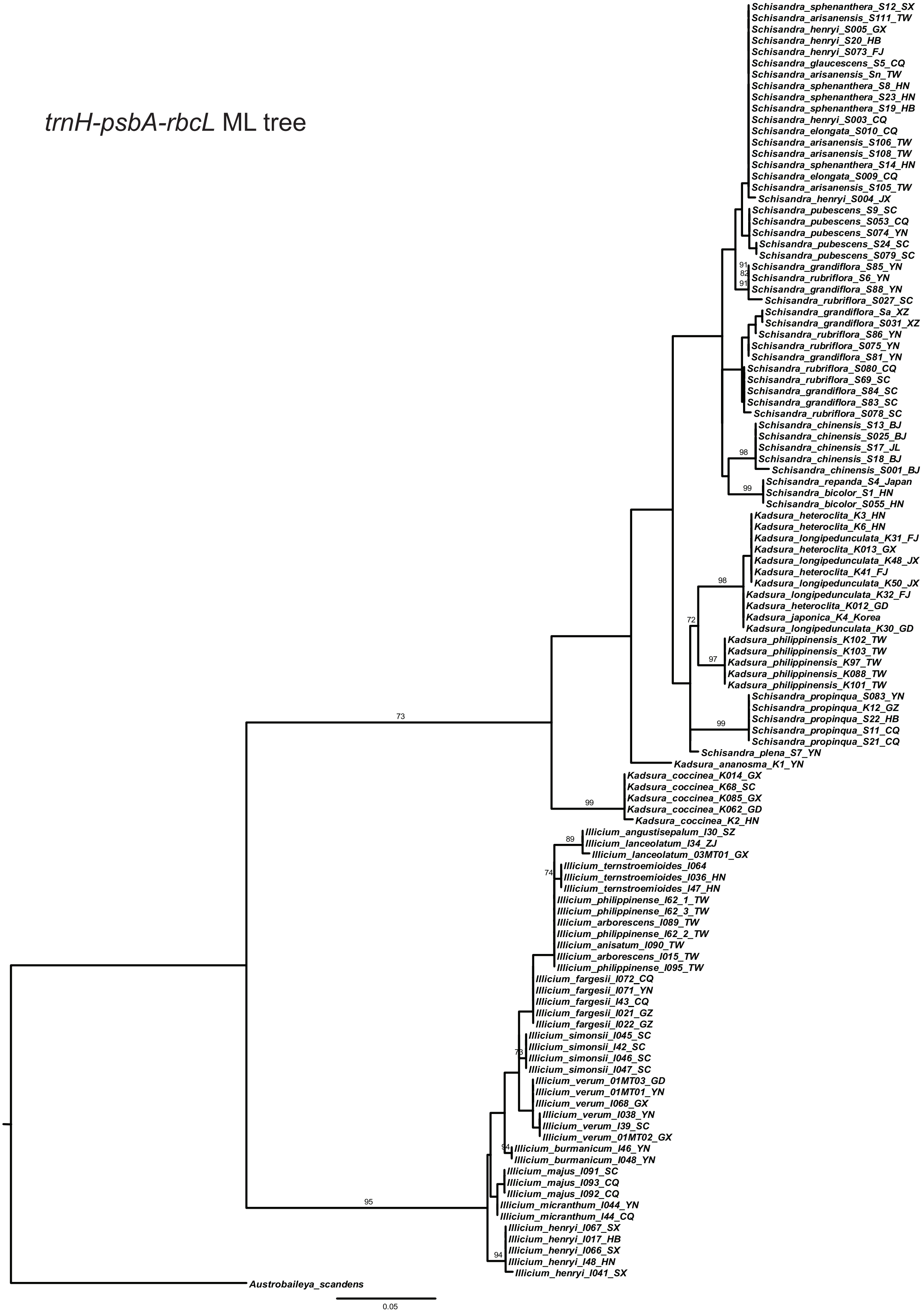
trnH-psbA-rbcL ML tree
Schisandra_sphenanthera_S12_SX
Schisandra_arisanensis_S111_TW
Schisandra_henryi_S005_GX
Schisandra_henryi_S20_HB
Schisandra_henryi_S073_FJ
Schisandra_glaucescens_S5_CQ
Schisandra_arisanensis_Sn_TW
Schisandra_sphenanthera_S8_HN
Schisandra_sphenanthera_S23_HN
Schisandra_sphenanthera_S19_HB
Schisandra_henryi_S003_CQ
Schisandra_elongata_S010_CQ
Schisandra_arisanensis_S106_TW
Schisandra_arisanensis_S108_TW
Schisandra_sphenanthera_S14_HN
Schisandra_elongata_S009_CQ
Schisandra_arisanensis_S105_TW
Schisandra_henryi_S004_JX
Schisandra_pubescens_S9_SC
Schisandra_pubescens_S053_CQ
Schisandra_pubescens_S074_YN
Schisandra_pubescens_S24_SC
Schisandra_pubescens_S079_SC
Schisandra_grandiflora_S85_YN
Schisandra_rubriflora_S6_YN
Schisandra_grandiflora_S88_YN
Schisandra_rubriflora_S027_SC
Schisandra_grandiflora_Sa_XZ
Schisandra_grandiflora_S031_XZ
Schisandra_rubriflora_S86_YN
Schisandra_rubriflora_S075_YN
Schisandra_grandiflora_S81_YN
Schisandra_rubriflora_S080_CQ
Schisandra_rubriflora_S69_SC
Schisandra_grandiflora_S84_SC
Schisandra_grandiflora_S83_SC
Schisandra_rubriflora_S078_SC
Schisandra_chinensis_S13_BJ
Schisandra_chinensis_S025_BJ
Schisandra_chinensis_S17_JL
Schisandra_chinensis_S18_BJ
Schisandra_chinensis_S001_BJ
Schisandra_repanda_S4_Japan
Schisandra_bicolor_S1_HN
Schisandra_bicolor_S055_HN
Kadsura_heteroclita_K3_HN
Kadsura_heteroclita_K6_HN
Kadsura_longipedunculata_K31_FJ
Kadsura_heteroclita_K013_GX
Kadsura_longipedunculata_K48_JX
Kadsura_heteroclita_K41_FJ
Kadsura_longipedunculata_K50_JX
Kadsura_longipedunculata_K32_FJ
Kadsura_heteroclita_K012_GD
Kadsura_japonica_K4_Korea
Kadsura_longipedunculata_K30_GD
Kadsura_philippinensis_K102_TW
Kadsura_philippinensis_K103_TW
Kadsura_philippinensis_K97_TW
Kadsura_philippinensis_K088_TW
Kadsura_philippinensis_K101_TW
Schisandra_propinqua_S083_YN
Schisandra_propinqua_K12_GZ
Schisandra_propinqua_S22_HB
Schisandra_propinqua_S11_CQ
Schisandra_propinqua_S21_CQ
Schisandra_plena_S7_YN
Kadsura_ananosma_K1_YN
Kadsura_coccinea_K014_GX
Kadsura_coccinea_K68_SC
Kadsura_coccinea_K085_GX
Kadsura_coccinea_K062_GD
Kadsura_coccinea_K2_HN
Illicium_angustisepalum_I30_SZ
Illicium_lanceolatum_I34_ZJ
Illicium_lanceolatum_03MT01_GX
Illicium_ternstroemioides_I064
Illicium_ternstroemioides_I036_HN
Illicium_ternstroemioides_I47_HN
Illicium_philippinense_I62_1_TW
Illicium_philippinense_I62_3_TW
Illicium_arborescens_I089_TW
Illicium_philippinense_I62_2_TW
Illicium_anisatum_I090_TW
Illicium_arborescens_I015_TW
Illicium_philippinense_I095_TW
Illicium_fargesii_I072_CQ
Illicium_fargesii_I071_YN
Illicium_fargesii_I43_CQ
Illicium_fargesii_I021_GZ
Illicium_fargesii_I022_GZ
Illicium_simonsii_I045_SC
Illicium_simonsii_I42_SC
Illicium_simonsii_I046_SC
Illicium_simonsii_I047_SC
Illicium_verum_01MT03_GD
Illicium_verum_01MT01_YN
Illicium_verum_I068_GX
Illicium_verum_I038_YN
Illicium_verum_I39_SC
Illicium_verum_01MT02_GX
Illicium_burmanicum_I46_YN
Illicium_burmanicum_I048_YN
Illicium_majus_I091_SC
Illicium_majus_I093_CQ
Illicium_majus_I092_CQ
Illicium_micranthum_I044_YN
Illicium_micranthum_I44_CQ
Illicium_henryi_I067_SX
Illicium_henryi_I017_HB
Illicium_henryi_I066_SX
Illicium_henryi_I48_HN
Illicium_henryi_I041_SX
Austrobaileya_scandens
0.05

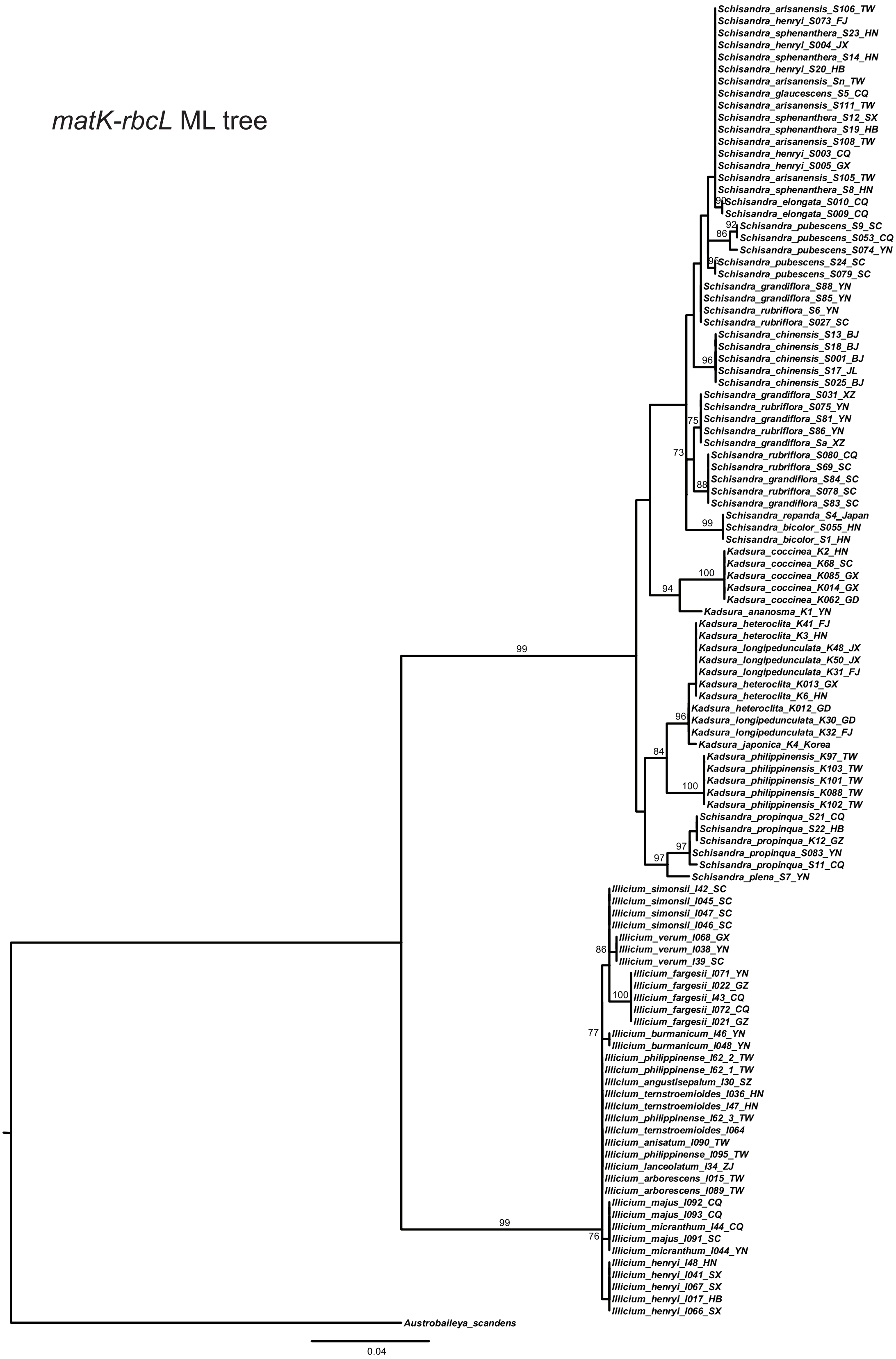
matK-rbcL ML tree
Schisandra_arisanensis_S106_TW
Schisandra_henryi_S073_FJ
Schisandra_sphenanthera_S23_HN
Schisandra_henryi_S004_JX
Schisandra_sphenanthera_S14_HN
Schisandra_henryi_S20_HB
Schisandra_arisanensis_Sn_TW
Schisandra_glaucescens_S5_CQ
Schisandra_arisanensis_S111_TW
Schisandra_sphenanthera_S12_SX
Schisandra_sphenanthera_S19_HB
Schisandra_arisanensis_S108_TW
Schisandra_henryi_S003_CQ
Schisandra_henryi_S005_GX
Schisandra_arisanensis_S105_TW
Schisandra_sphenanthera_S8_HN
Schisandra_elongata_S010_CQ
Schisandra_elongata_S009_CQ
Schisandra_pubescens_S9_SC
Schisandra_pubescens_S053_CQ
Schisandra_pubescens_S074_YN
Schisandra_pubescens_S24_SC
Schisandra_pubescens_S079_SC
Schisandra_grandiflora_S88_YN
Schisandra_grandiflora_S85_YN
Schisandra_rubriflora_S6_YN
Schisandra_rubriflora_S027_SC
Schisandra_chinensis_S13_BJ
Schisandra_chinensis_S18_BJ
Schisandra_chinensis_S001_BJ
Schisandra_chinensis_S17_JL
Schisandra_chinensis_S025_BJ
Schisandra_grandiflora_S031_XZ
Schisandra_rubriflora_S075_YN
Schisandra_grandiflora_S81_YN
Schisandra_rubriflora_S86_YN
Schisandra_grandiflora_Sa_XZ
Schisandra_rubriflora_S080_CQ
Schisandra_rubriflora_S69_SC
Schisandra_grandiflora_S84_SC
Schisandra_rubriflora_S078_SC
Schisandra_grandiflora_S83_SC
Schisandra_repanda_S4_Japan
Schisandra_bicolor_S055_HN
Schisandra_bicolor_S1_HN
Kadsura_coccinea_K2_HN
Kadsura_coccinea_K68_SC
Kadsura_coccinea_K085_GX
Kadsura_coccinea_K014_GX
Kadsura_coccinea_K062_GD
Kadsura_ananosma_K1_YN
Kadsura_heteroclita_K41_FJ
Kadsura_heteroclita_K3_HN
Kadsura_longipedunculata_K48_JX
Kadsura_longipedunculata_K50_JX
Kadsura_longipedunculata_K31_FJ
Kadsura_heteroclita_K013_GX
Kadsura_heteroclita_K6_HN
Kadsura_heteroclita_K012_GD
Kadsura_longipedunculata_K30_GD
Kadsura_longipedunculata_K32_FJ
Kadsura_japonica_K4_Korea
Kadsura_philippinensis_K97_TW
Kadsura_philippinensis_K103_TW
Kadsura_philippinensis_K101_TW
Kadsura_philippinensis_K088_TW
Kadsura_philippinensis_K102_TW
Schisandra_propinqua_S21_CQ
Schisandra_propinqua_S22_HB
Schisandra_propinqua_K12_GZ
Schisandra_propinqua_S083_YN
Schisandra_propinqua_S11_CQ
Schisandra_plena_S7_YN
Illicium_simonsii_I42_SC
Illicium_simonsii_I045_SC
Illicium_simonsii_I047_SC
Illicium_simonsii_I046_SC
Illicium_verum_I068_GX
Illicium_verum_I038_YN
Illicium_verum_I39_SC
Illicium_fargesii_I071_YN
Illicium_fargesii_I022_GZ
Illicium_fargesii_I43_CQ
Illicium_fargesii_I072_CQ
Illicium_fargesii_I021_GZ
Illicium_burmanicum_I46_YN
Illicium_burmanicum_I048_YN
Illicium_philippinense_I62_2_TW
Illicium_philippinense_I62_1_TW
Illicium_angustisepalum_I30_SZ
Illicium_ternstroemioides_I036_HN
Illicium_ternstroemioides_I47_HN
Illicium_philippinense_I62_3_TW
Illicium_ternstroemioides_I064
Illicium_anisatum_I090_TW
Illicium_philippinense_I095_TW
Illicium_lanceolatum_I34_ZJ
Illicium_arborescens_I015_TW
Illicium_arborescens_I089_TW
Illicium_majus_I092_CQ
Illicium_majus_I093_CQ
Illicium_micranthum_I44_CQ
Illicium_majus_I091_SC
Illicium_micranthum_I044_YN
Illicium_henryi_I48_HN
Illicium_henryi_I041_SX
Illicium_henryi_I067_SX
Illicium_henryi_I017_HB
Illicium_henryi_I066_SX
Austrobaileya_scandens
0.04

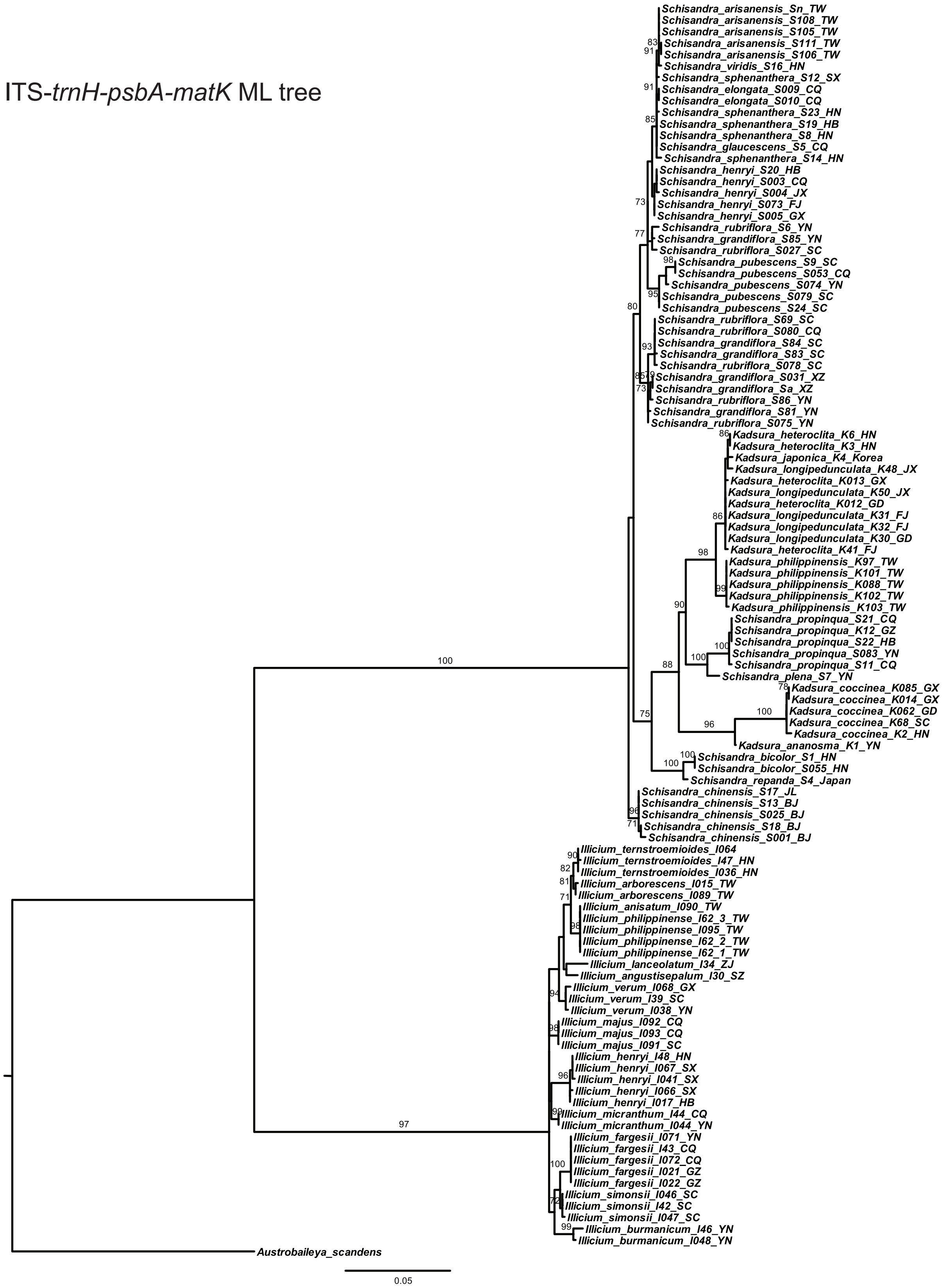
ITS-trnH-psbA-matK ML tree
Schisandra_arisanensis_Sn_TW
Schisandra_arisanensis_S108_TW
Schisandra_arisanensis_S105_TW
Schisandra_arisanensis_S111_TW
Schisandra_arisanensis_S106_TW
Schisandra_viridis_S16_HN
Schisandra_sphenanthera_S12_SX
Schisandra_elongata_S009_CQ
Schisandra_elongata_S010_CQ
Schisandra_sphenanthera_S23_HN
Schisandra_sphenanthera_S19_HB
Schisandra_sphenanthera_S8_HN
Schisandra_glaucescens_S5_CQ
Schisandra_sphenanthera_S14_HN
Schisandra_henryi_S20_HB
Schisandra_henryi_S003_CQ
Schisandra_henryi_S004_JX
Schisandra_henryi_S073_FJ
Schisandra_henryi_S005_GX
Schisandra_rubriflora_S6_YN
Schisandra_grandiflora_S85_YN
Schisandra_rubriflora_S027_SC
Schisandra_pubescens_S9_SC
Schisandra_pubescens_S053_CQ
Schisandra_pubescens_S074_YN
Schisandra_pubescens_S079_SC
Schisandra_pubescens_S24_SC
Schisandra_rubriflora_S69_SC
Schisandra_rubriflora_S080_CQ
Schisandra_grandiflora_S84_SC
Schisandra_grandiflora_S83_SC
Schisandra_rubriflora_S078_SC
Schisandra_grandiflora_S031_XZ
Schisandra_grandiflora_Sa_XZ
Schisandra_rubriflora_S86_YN
Schisandra_grandiflora_S81_YN
Schisandra_rubriflora_S075_YN
Kadsura_heteroclita_K6_HN
Kadsura_heteroclita_K3_HN
Kadsura_japonica_K4_Korea
Kadsura_longipedunculata_K48_JX
Kadsura_heteroclita_K013_GX
Kadsura_longipedunculata_K50_JX
Kadsura_heteroclita_K012_GD
Kadsura_longipedunculata_K31_FJ
Kadsura_longipedunculata_K32_FJ
Kadsura_longipedunculata_K30_GD
Kadsura_heteroclita_K41_FJ
Kadsura_philippinensis_K97_TW
Kadsura_philippinensis_K101_TW
Kadsura_philippinensis_K088_TW
Kadsura_philippinensis_K102_TW
Kadsura_philippinensis_K103_TW
Schisandra_propinqua_S21_CQ
Schisandra_propinqua_K12_GZ
Schisandra_propinqua_S22_HB
Schisandra_propinqua_S083_YN
Schisandra_propinqua_S11_CQ
Schisandra_plena_S7_YN
Kadsura_coccinea_K085_GX
Kadsura_coccinea_K014_GX
Kadsura_coccinea_K062_GD
Kadsura_coccinea_K68_SC
Kadsura_coccinea_K2_HN
Kadsura_ananosma_K1_YN
Schisandra_bicolor_S1_HN
Schisandra_bicolor_S055_HN
Schisandra_repanda_S4_Japan
Schisandra_chinensis_S17_JL
Schisandra_chinensis_S13_BJ
Schisandra_chinensis_S025_BJ
Schisandra_chinensis_S18_BJ
Schisandra_chinensis_S001_BJ
Illicium_ternstroemioides_I064
Illicium_ternstroemioides_I47_HN
Illicium_ternstroemioides_I036_HN
Illicium_arborescens_I015_TW
Illicium_arborescens_I089_TW
Illicium_anisatum_I090_TW
Illicium_philippinense_I62_3_TW
Illicium_philippinense_I095_TW
Illicium_philippinense_I62_2_TW
Illicium_philippinense_I62_1_TW
Illicium_lanceolatum_I34_ZJ
Illicium_angustisepalum_I30_SZ
Illicium_verum_I068_GX
Illicium_verum_I39_SC
Illicium_verum_I038_YN
Illicium_majus_I092_CQ
Illicium_majus_I093_CQ
Illicium_majus_I091_SC
Illicium_henryi_I48_HN
Illicium_henryi_I067_SX
Illicium_henryi_I041_SX
Illicium_henryi_I066_SX
Illicium_henryi_I017_HB
Illicium_micranthum_I44_CQ
Illicium_micranthum_I044_YN
Illicium_fargesii_I071_YN
Illicium_fargesii_I43_CQ
Illicium_fargesii_I072_CQ
Illicium_fargesii_I021_GZ
Illicium_fargesii_I022_GZ
Illicium_simonsii_I046_SC
Illicium_simonsii_I42_SC
Illicium_simonsii_I047_SC
Illicium_burmanicum_I46_YN
Illicium_burmanicum_I048_YN
Austrobaileya_scandens
0.05

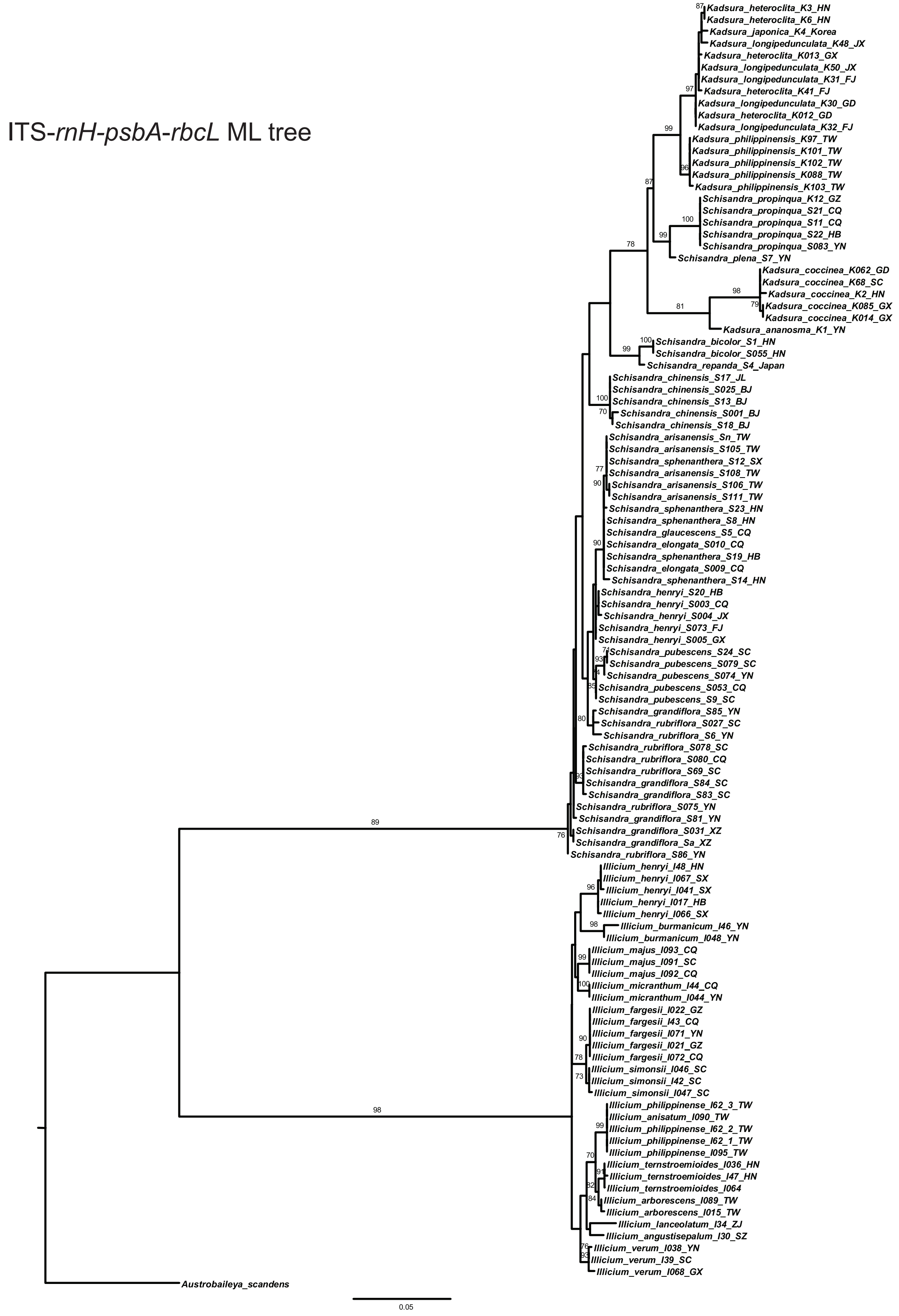
ITS-rnH-psbA-rbcL ML tree
Kadsura_heteroclita_K3_HN
Kadsura_heteroclita_K6_HN
Kadsura_japonica_K4_Korea
Kadsura_longipedunculata_K48_JX
Kadsura_heteroclita_K013_GX
Kadsura_longipedunculata_K50_JX
Kadsura_longipedunculata_K31_FJ
Kadsura_heteroclita_K41_FJ
Kadsura_longipedunculata_K30_GD
Kadsura_heteroclita_K012_GD
Kadsura_longipedunculata_K32_FJ
Kadsura_philippinensis_K97_TW
Kadsura_philippinensis_K101_TW
Kadsura_philippinensis_K102_TW
Kadsura_philippinensis_K088_TW
Kadsura_philippinensis_K103_TW
Schisandra_propinqua_K12_GZ
Schisandra_propinqua_S21_CQ
Schisandra_propinqua_S11_CQ
Schisandra_propinqua_S22_HB
Schisandra_propinqua_S083_YN
Schisandra_plena_S7_YN
Kadsura_coccinea_K062_GD
Kadsura_coccinea_K68_SC
Kadsura_coccinea_K2_HN
Kadsura_coccinea_K085_GX
Kadsura_coccinea_K014_GX
Kadsura_ananosma_K1_YN
Schisandra_bicolor_S1_HN
Schisandra_bicolor_S055_HN
Schisandra_repanda_S4_Japan
Schisandra_chinensis_S17_JL
Schisandra_chinensis_S025_BJ
Schisandra_chinensis_S13_BJ
Schisandra_chinensis_S001_BJ
Schisandra_chinensis_S18_BJ
Schisandra_arisanensis_Sn_TW
Schisandra_arisanensis_S105_TW
Schisandra_sphenanthera_S12_SX
Schisandra_arisanensis_S108_TW
Schisandra_arisanensis_S106_TW
Schisandra_arisanensis_S111_TW
Schisandra_sphenanthera_S23_HN
Schisandra_sphenanthera_S8_HN
Schisandra_glaucescens_S5_CQ
Schisandra_elongata_S010_CQ
Schisandra_sphenanthera_S19_HB
Schisandra_elongata_S009_CQ
Schisandra_sphenanthera_S14_HN
Schisandra_henryi_S20_HB
Schisandra_henryi_S003_CQ
Schisandra_henryi_S004_JX
Schisandra_henryi_S073_FJ
Schisandra_henryi_S005_GX
Schisandra_pubescens_S24_SC
Schisandra_pubescens_S079_SC
Schisandra_pubescens_S074_YN
Schisandra_pubescens_S053_CQ
Schisandra_pubescens_S9_SC
Schisandra_grandiflora_S85_YN
Schisandra_rubriflora_S027_SC
Schisandra_rubriflora_S6_YN
Schisandra_rubriflora_S078_SC
Schisandra_rubriflora_S080_CQ
Schisandra_rubriflora_S69_SC
Schisandra_grandiflora_S84_SC
Schisandra_grandiflora_S83_SC
Schisandra_rubriflora_S075_YN
Schisandra_grandiflora_S81_YN
Schisandra_grandiflora_S031_XZ
Schisandra_grandiflora_Sa_XZ
Schisandra_rubriflora_S86_YN
Illicium_henryi_I48_HN
Illicium_henryi_I067_SX
Illicium_henryi_I041_SX
Illicium_henryi_I017_HB
Illicium_henryi_I066_SX
Illicium_burmanicum_I46_YN
Illicium_burmanicum_I048_YN
Illicium_majus_I093_CQ
Illicium_majus_I091_SC
Illicium_majus_I092_CQ
Illicium_micranthum_I44_CQ
Illicium_micranthum_I044_YN
Illicium_fargesii_I022_GZ
Illicium_fargesii_I43_CQ
Illicium_fargesii_I071_YN
Illicium_fargesii_I021_GZ
Illicium_fargesii_I072_CQ
Illicium_simonsii_I046_SC
Illicium_simonsii_I42_SC
Illicium_simonsii_I047_SC
Illicium_philippinense_I62_3_TW
Illicium_anisatum_I090_TW
Illicium_philippinense_I62_2_TW
Illicium_philippinense_I62_1_TW
Illicium_philippinense_I095_TW
Illicium_ternstroemioides_I036_HN
Illicium_ternstroemioides_I47_HN
Illicium_ternstroemioides_I064
Illicium_arborescens_I089_TW
Illicium_arborescens_I015_TW
Illicium_lanceolatum_I34_ZJ
Illicium_angustisepalum_I30_SZ
Illicium_verum_I038_YN
Illicium_verum_I39_SC
Illicium_verum_I068_GX
Austrobaileya_scandens
0.05

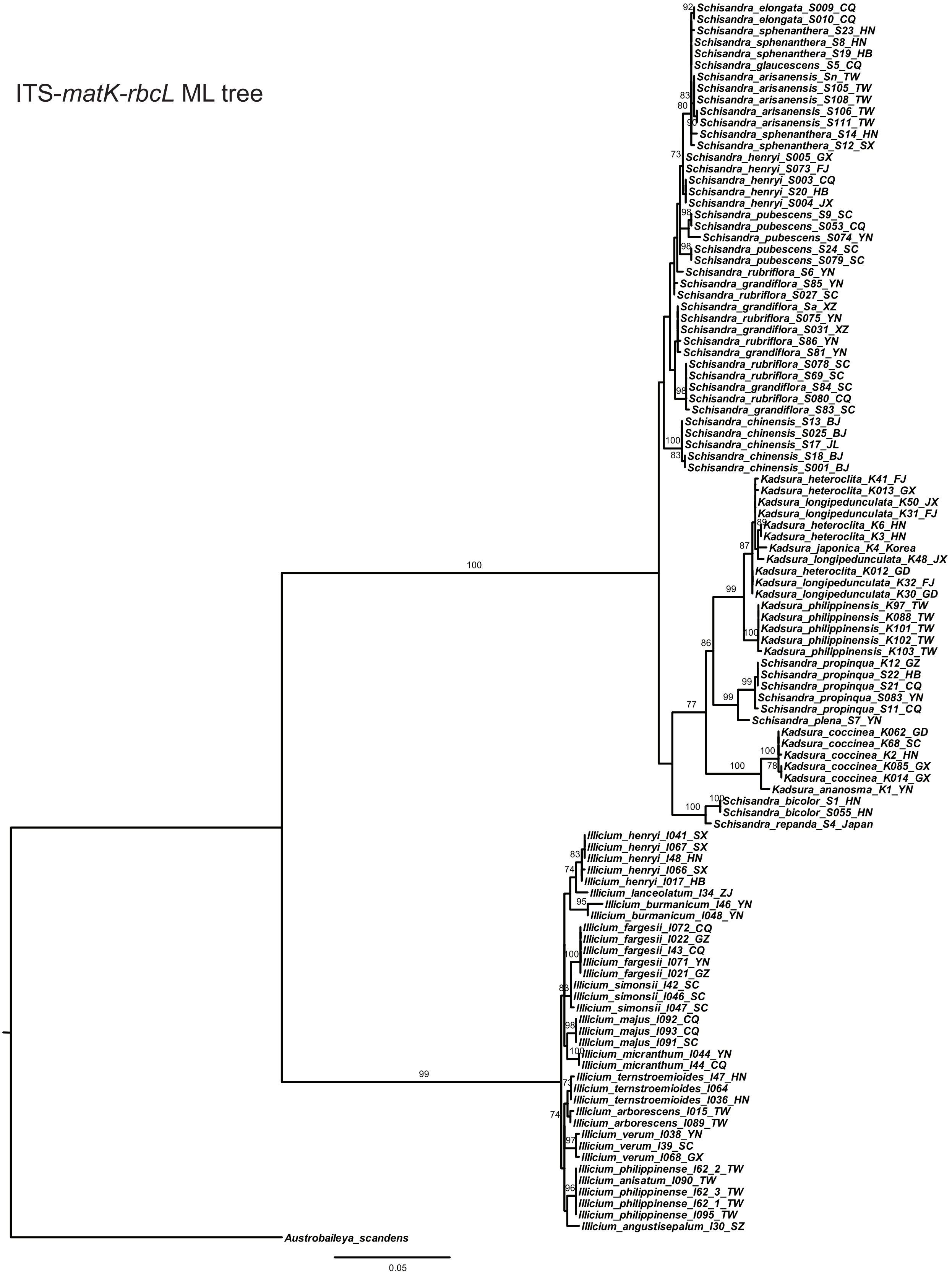
ITS-matK-rbcL ML tree
Schisandra_elongata_S009_CQ
Schisandra_elongata_S010_CQ
Schisandra_sphenanthera_S23_HN
Schisandra_sphenanthera_S8_HN
Schisandra_sphenanthera_S19_HB
Schisandra_glaucescens_S5_CQ
Schisandra_arisanensis_Sn_TW
Schisandra_arisanensis_S105_TW
Schisandra_arisanensis_S108_TW
Schisandra_arisanensis_S106_TW
Schisandra_arisanensis_S111_TW
Schisandra_sphenanthera_S14_HN
Schisandra_sphenanthera_S12_SX
Schisandra_henryi_S005_GX
Schisandra_henryi_S073_FJ
Schisandra_henryi_S003_CQ
Schisandra_henryi_S20_HB
Schisandra_henryi_S004_JX
Schisandra_pubescens_S9_SC
Schisandra_pubescens_S053_CQ
Schisandra_pubescens_S074_YN
Schisandra_pubescens_S24_SC
Schisandra_pubescens_S079_SC
Schisandra_rubriflora_S6_YN
Schisandra_grandiflora_S85_YN
Schisandra_rubriflora_S027_SC
Schisandra_grandiflora_Sa_XZ
Schisandra_rubriflora_S075_YN
Schisandra_grandiflora_S031_XZ
Schisandra_rubriflora_S86_YN
Schisandra_grandiflora_S81_YN
Schisandra_rubriflora_S078_SC
Schisandra_rubriflora_S69_SC
Schisandra_grandiflora_S84_SC
Schisandra_rubriflora_S080_CQ
Schisandra_grandiflora_S83_SC
Schisandra_chinensis_S13_BJ
Schisandra_chinensis_S025_BJ
Schisandra_chinensis_S17_JL
Schisandra_chinensis_S18_BJ
Schisandra_chinensis_S001_BJ
Kadsura_heteroclita_K41_FJ
Kadsura_heteroclita_K013_GX
Kadsura_longipedunculata_K50_JX
Kadsura_longipedunculata_K31_FJ
Kadsura_heteroclita_K6_HN
Kadsura_heteroclita_K3_HN
Kadsura_japonica_K4_Korea
Kadsura_longipedunculata_K48_JX
Kadsura_heteroclita_K012_GD
Kadsura_longipedunculata_K32_FJ
Kadsura_longipedunculata_K30_GD
Kadsura_philippinensis_K97_TW
Kadsura_philippinensis_K088_TW
Kadsura_philippinensis_K101_TW
Kadsura_philippinensis_K102_TW
Kadsura_philippinensis_K103_TW
Schisandra_propinqua_K12_GZ
Schisandra_propinqua_S22_HB
Schisandra_propinqua_S21_CQ
Schisandra_propinqua_S083_YN
Schisandra_propinqua_S11_CQ
Schisandra_plena_S7_YN
Kadsura_coccinea_K062_GD
Kadsura_coccinea_K68_SC
Kadsura_coccinea_K2_HN
Kadsura_coccinea_K085_GX
Kadsura_coccinea_K014_GX
Kadsura_ananosma_K1_YN
Schisandra_bicolor_S1_HN
Schisandra_bicolor_S055_HN
Schisandra_repanda_S4_Japan
Illicium_henryi_I041_SX
Illicium_henryi_I067_SX
Illicium_henryi_I48_HN
Illicium_henryi_I066_SX
Illicium_henryi_I017_HB
Illicium_lanceolatum_I34_ZJ
Illicium_burmanicum_I46_YN
Illicium_burmanicum_I048_YN
Illicium_fargesii_I072_CQ
Illicium_fargesii_I022_GZ
Illicium_fargesii_I43_CQ
Illicium_fargesii_I071_YN
Illicium_fargesii_I021_GZ
Illicium_simonsii_I42_SC
Illicium_simonsii_I046_SC
Illicium_simonsii_I047_SC
Illicium_majus_I092_CQ
Illicium_majus_I093_CQ
Illicium_majus_I091_SC
Illicium_micranthum_I044_YN
Illicium_micranthum_I44_CQ
Illicium_ternstroemioides_I47_HN
Illicium_ternstroemioides_I064
Illicium_ternstroemioides_I036_HN
Illicium_arborescens_I015_TW
Illicium_arborescens_I089_TW
Illicium_verum_I038_YN
Illicium_verum_I39_SC
Illicium_verum_I068_GX
Illicium_philippinense_I62_2_TW
Illicium_anisatum_I090_TW
Illicium_philippinense_I62_3_TW
Illicium_philippinense_I62_1_TW
Illicium_philippinense_I095_TW
Illicium_angustisepalum_I30_SZ
Austrobaileya_scandens
0.05

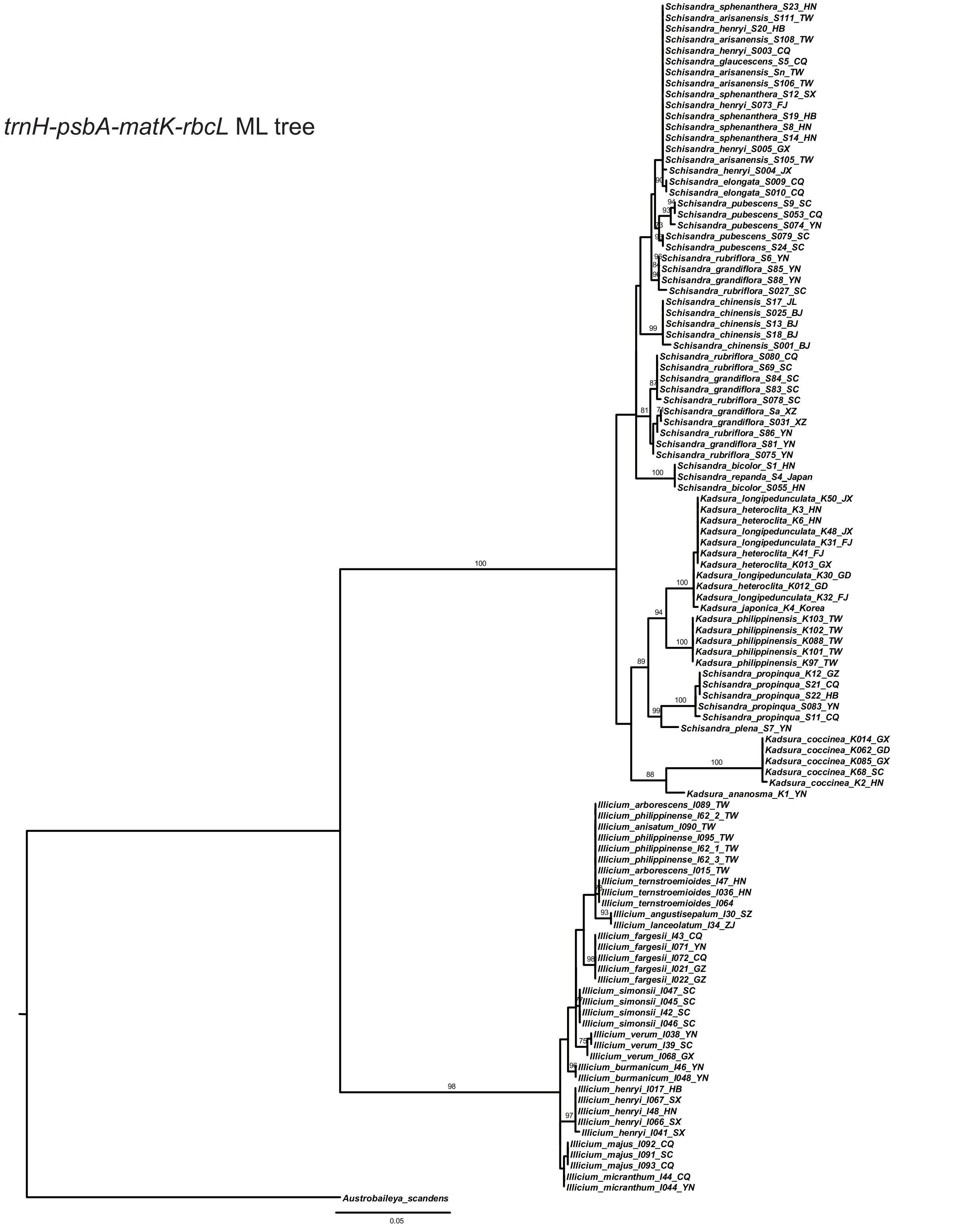
trnH-psbA-matK-rbcL ML tree
Schisandra_sphenanthera_S23_HN
Schisandra_arisanensis_S111_TW
Schisandra_henryi_S20_HB
Schisandra_arisanensis_S108_TW
Schisandra_henryi_S003_CQ
Schisandra_glaucescens_S5_CQ
Schisandra_arisanensis_Sn_TW
Schisandra_arisanensis_S106_TW
Schisandra_sphenanthera_S12_SX
Schisandra_henryi_S073_FJ
Schisandra_sphenanthera_S19_HB
Schisandra_sphenanthera_S8_HN
Schisandra_sphenanthera_S14_HN
Schisandra_henryi_S005_GX
Schisandra_arisanensis_S105_TW
Schisandra_henryi_S004_JX
90
Schisandra_elongata_S009_CQ
Schisandra_elongata_S010_CQ
Schisandra_pubescens_S9_SC
93
Schisandra_pubescens_S053_CQ
Schisandra_pubescens_S074_YN
Schisandra_pubescens_S079_SC
Schisandra_pubescens_S24_SC
Schisandra_rubriflora_S6_YN
Schisandra_grandiflora_S85_YN
Schisandra_grandiflora_S88_YN
Schisandra_rubriflora_S027_SC
Schisandra_chinensis_S17_JL
Schisandra_chinensis_S025_BJ
99
Schisandra_chinensis_S13_BJ
Schisandra_chinensis_S18_BJ
Schisandra_chinensis_S001_BJ
Schisandra_rubriflora_S080_CQ
Schisandra_rubriflora_S69_SC
Schisandra_grandiflora_S84_SC
87
Schisandra_grandiflora_S83_SC
Schisandra_rubriflora_S078_SC
81
Schisandra_grandiflora_Sa_XZ
Schisandra_grandiflora_S031_XZ
Schisandra_rubriflora_S86_YN
Schisandra_grandiflora_S81_YN
Schisandra_rubriflora_S075_YN
Schisandra_bicolor_S1_HN
100
Schisandra_repanda_S4_Japan
Schisandra_bicolor_S055_HN
Kadsura_longipedunculata_K50_JX
Kadsura_heteroclita_K3_HN
Kadsura_heteroclita_K6_HN
Kadsura_longipedunculata_K48_JX
Kadsura_longipedunculata_K31_FJ
Kadsura_heteroclita_K41_FJ
Kadsura_heteroclita_K013_GX
100
Kadsura_longipedunculata_K30_GD
Kadsura_heteroclita_K012_GD
100
Kadsura_longipedunculata_K32_FJ
Kadsura_japonica_K4_Korea
94
Kadsura_philippinensis_K103_TW
Kadsura_philippinensis_K102_TW
100
Kadsura_philippinensis_K088_TW
Kadsura_philippinensis_K101_TW
89
Kadsura_philippinensis_K97_TW
Schisandra_propinqua_K12_GZ
Schisandra_propinqua_S21_CQ
Schisandra_propinqua_S22_HB
100
Schisandra_propinqua_S083_YN
99
Schisandra_propinqua_S11_CQ
Schisandra_plena_S7_YN
Kadsura_coccinea_K014_GX
Kadsura_coccinea_K062_GD
100
Kadsura_coccinea_K085_GX
Kadsura_coccinea_K68_SC
88
Kadsura_coccinea_K2_HN
Kadsura_ananosma_K1_YN
Illicium_arborescens_I089_TW
Illicium_philippinense_I62_2_TW
Illicium_anisatum_I090_TW
Illicium_philippinense_I095_TW
Illicium_philippinense_I62_1_TW
Illicium_philippinense_I62_3_TW
Illicium_arborescens_I015_TW
Illicium_ternstroemioides_I47_HN
Illicium_ternstroemioides_I036_HN
Illicium_ternstroemioides_I064
93
Illicium_angustisepalum_I30_SZ
Illicium_lanceolatum_I34_ZJ
Illicium_fargesii_I43_CQ
Illicium_fargesii_I071_YN
98
Illicium_fargesii_I072_CQ
Illicium_fargesii_I021_GZ
Illicium_fargesii_I022_GZ
Illicium_simonsii_I047_SC
Illicium_simonsii_I045_SC
Illicium_simonsii_I42_SC
Illicium_simonsii_I046_SC
Illicium_verum_I038_YN
75
Illicium_verum_I39_SC
Illicium_verum_I068_GX
96
Illicium_burmanicum_I46_YN
Illicium_burmanicum_I048_YN
98
Illicium_henryi_I017_HB
Illicium_henryi_I067_SX
97
Illicium_henryi_I48_HN
Illicium_henryi_I066_SX
Illicium_henryi_I041_SX
Illicium_majus_I092_CQ
Illicium_majus_I091_SC
Illicium_majus_I093_CQ
Illicium_micranthum_I44_CQ
Illicium_micranthum_I044_YN
Austrobaileya_scandens
0.05

ITS-*trnH-psbA-matK-rbcL* ML tree

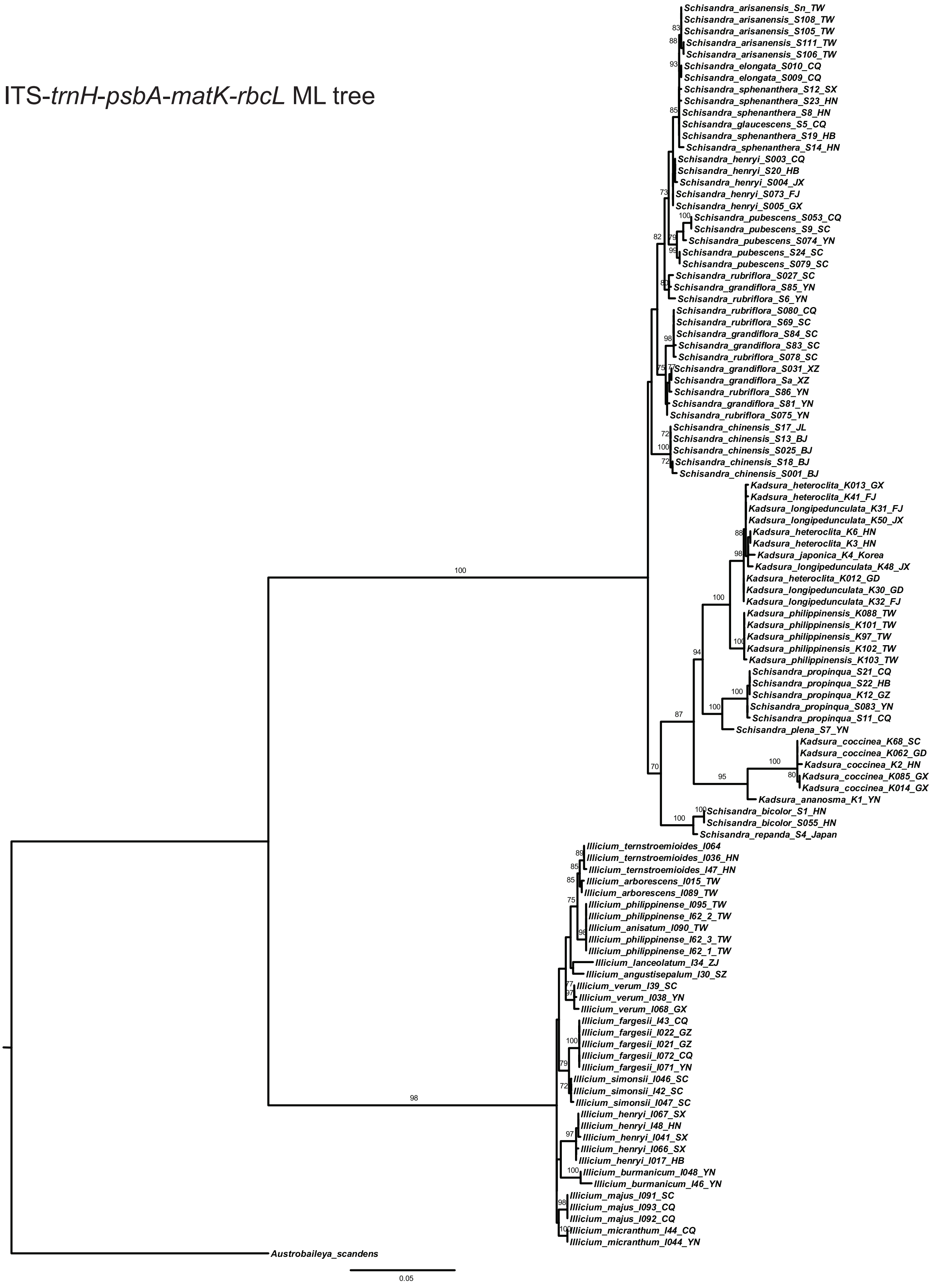

Supplement: S1 Fig — Numbers above the branches represent bootstrap values (≥70%) for monophyletic species. The asterisk indicates the bootstrap value or posterior probability lower than the threshold. ML, maximum-likelihood method. (PDF) [file pone.0125574.s001.pdf]
