# Supplementary material for: Evaluation of Four Commonly Used DNA Barcoding Loci for Chinese Medicinal Plants of the Family Schisandraceae
Source: PLoS One. 2015 May 4;10(5):e0125574. doi: 10.1371/journal.pone.0125574 (PMC4418597; doi:10.1371/journal.pone.0125574)
Supplement: S2 Table — (DOC) [file pone.0125574.s004.doc]

**S2 Table. The primer information and** **optimal PCR conditions used in this study.**

| Barcoding locus | Primer | Sequence (5’-3’) | PCR condition | References |
| --- | --- | --- | --- | --- |
| ITS | ITS5 | CCT TAT CAT TTA GAG GAA GGA G | 95℃ 5min;  {95℃ 30s, 48℃ 1min, 72℃ 90s }*37;  72℃ 10min | [15] |
|  | ITS4 | TCC TCC GCT TAT TGA TAT GC |  | [69] |
| *trnH-psbA* | psbA3_f | GTT ATG CAT GAA CGT AAT GCT C | 94℃ 5min;  {94℃ 45s, 50℃ 45s, 72℃ 50s }*32;  72℃ 5min | [70] |
|  | trnHf_05 | CGC GCA TGG TGG ATT CAC AAT CC |  | [71] |
| *matK* | matK_390f | CGA TCT ATT CAT TCA ATA TTT C | 95℃ 5min;  {95℃ 30s, 50℃ 1min, 72℃ 40s }*37;  72℃ 10min | [72] |
|  | matK_1326r | TCT AGC ACA CGA AAG TCG AAG T |  | [72] |
| *rbcL* | rbcL-1F | ATG TCA CCA CAA ACA GAA ACT | 95℃ 5min;  { 95℃ 30s, 50℃ 1min, 72℃ 40s }*37;  72℃ 10min | [73] |
|  | rbcL-724R | TCG CAT GTA CCT GCA GTA GC |  | [74] |
