# Supplementary material for: Evaluation of Four Commonly Used DNA Barcoding Loci for Chinese Medicinal Plants of the Family Schisandraceae
Source: PLoS One. 2015 May 4;10(5):e0125574. doi: 10.1371/journal.pone.0125574 (PMC4418597; doi:10.1371/journal.pone.0125574)
Supplement: S3 Table — (DOC) [file pone.0125574.s005.doc]

**S3 Table.** **Discriminatory power of single regions and their combinations based on the genera data (*Schisandra*/*Kadsura* and *Illicium*).**

| DNA barcodes | N^*^ | Ability to discriminate | Ability to discriminate (ML)^$^ (%) | | Ability to discriminate  (BI) ^$^ (%) | | Ability to discriminate  (character)^&^ (%) |
| --- | --- | --- | --- | --- | --- | --- | --- |
|  |  | (distance) ^#^ (%) | I | II | I | II |  |
| *Schisandra*+*Kadsura* |  |  |  |  |  |  |  |
| ITS1 | 19 | 36.84 | 31.58 | 21.05 | 26.32 | 15.79 | 15.79 |
| ITS2 | 19 | 21.05 | 21.05 | 15.79 | 10.53 | 5.26 | 10.53 |
| ITS | 19 | 36.84 | 31.58 | 21.05 | 26.32 | 10.53 | 15.79 |
| *trnH-psbA* | 14 | 42.86 | 42.86 | 28.57 | 28.57 | 7.14 | 35.71 |
| *matK* | 17 | 41.18 | 35.29 | 29.41 | 23.53 | 17.65 | 17.65 |
| *rbcL* | 16 | 25.00 | 25.00 | 25.00 | 25.00 | 25.00 | 25.00 |
| ITS+*trnH-psbA* | 14 | 42.86 | 57.14 | 35.71 | 50.00 | 28.57 | - |
| ITS*+matK* | 14 | 57.14 | 50.00 | 42.86 | 50.00 | 42.86 | - |
| ITS*+rbcL* | 16 | 50.00 | 50.00 | 50.00 | 50.00 | 43.75 | - |
| *trnH-psbA+matK* | 14 | 42.86 | 42.86 | 35.71 | 42.86 | 35.71 | - |
| *trnH-psbA+rbcL* | 14 | 42.86 | 35.71 | 28.57 | 35.71 | 35.71 | - |
| *matK+rbcL* | 14 | 42.86 | 42.86 | 35.71 | 35.71 | 28.57 | - |
| ITS*+trnH-psbA+matK* | 14 | 57.14 | 57.14 | 42.86 | 50.00 | 50.00 | - |
| ITS*+trnH-psbA+rbcL* | 14 | 42.86 | 50.00 | 42.86 | 42.86 | 42.86 | - |
| ITS*+matK+rbcL* | 14 | 57.14 | 57.14 | 50.00 | 57.14 | 50.00 | - |
| *trnH-psbA+matK+rbcL* | 14 | 42.86 | 42.86 | 42.86 | 42.86 | 42.86 | - |
| ITS*+trnH-psbA+matK+rbcL* | 14 | 57.14 | 64.29 | 57.14 | 57.14 | 57.14 | - |
| *Illicium* |  |  |  |  |  |  |  |
| ITS1 | 13 | 38.46 | 61.54 | 30.77 | 61.54 | 30.77 | 61.54 |
| ITS2 | 13 | 30.77 | 46.15 | 15.38 | 15.38 | 7.69 | 30.77 |
| ITS | 13 | 41.15 | 76.92 | 53.85 | 69.23 | 38.46 | 76.92 |
| *trnH-psbA* | 11 | 81.82 | 54.55 | 36.36 | 45.45 | 36.36 | 45.45 |
| *matK* | 11 | 36.36 | 36.36 | 36.36 | 36.36 | 9.09 | 36.36 |
| *rbcL* | 11 | 0.00 | 0.00 | 0.00 | 0.00 | 0.00 | 0.00 |
| ITS+*trnH-psbA* | 10 | 100.00 | 90.00 | 90.00 | 90.00 | 90.00 | - |
| ITS*+matK* | 10 | 100.00 | 80.00 | 80.00 | 80.00 | 70.00 | - |
| ITS*+rbcL* | 11 | 81.82 | 81.82 | 72.73 | 72.73 | 54.55 | - |
| *trnH-psbA+matK* | 10 | 80.00 | 70.00 | 50.00 | 70.00 | 60.00 | - |
| *trnH-psbA+rbcL* | 11 | 81.82 | 63.64 | 36.36 | 54.55 | 45.45 | - |
| *matK+rbcL* | 10 | 50.00 | 50.00 | 30.00 | 40.00 | 30.00 | - |
| ITS*+trnH-psbA+matK* | 10 | 100.00 | 90.00 | 90.00 | 90.00 | 90.00 | - |
| ITS*+trnH-psbA+rbcL* | 10 | 100.00 | 90.00 | 90.00 | 90.00 | 90.00 | - |
| ITS*+matK+rbcL* | 10 | 100.00 | 80.00 | 80.00 | 80.00 | 70.00 | - |
| *trnH-psbA+matK+rbcL* | 10 | 80.00 | 80.00 | 60.00 | 70.00 | 70.00 | - |
| ITS*+trnH-psbA+matK+rbcL* | 10 | 100.00 | 90.00 | 90.00 | 90.00 | 90.00 | - |

^*^ Species represented by multiple individuals.

^#^ The percentage of the species with higher minimum interspecific distance than maximum intraspecific distance (species barcoding gap) among studied species.

^$^ Column I: the percentage of the monophyletic clusters of individuals belonging to the same morphological species among studied species. Column II: the corresponding number for the monophyletic clusters with ≥70% bootstrap values in ML and ≥0.95 posterior probabilities in BI. ML, maximum-likelihood method; BI, Bayesian-inference method.

^&^ The percentage of the species that could be identified by diagnostic characters among studied species. The analyses did not include multi-locus combinations.
