# Supplementary material for: Evaluation of Four Commonly Used DNA Barcoding Loci for Chinese Medicinal Plants of the Family Schisandraceae
Source: PLoS One. 2015 May 4;10(5):e0125574. doi: 10.1371/journal.pone.0125574 (PMC4418597; doi:10.1371/journal.pone.0125574)
Supplement: S4 Table — (DOC) [file pone.0125574.s006.doc]

**S4 Table. Identification success rates of single regions and their combinations using TAXONDNA program under ‘best match’ and ‘best close match’ methods based on the genera data (*Schisandra*/*Kadsura* and *Illicium*).**

| DNA barcodes | N^*^ species | N seqeunces | Best match (%) | | | Best close match (%) | | | | Threshold (%) |
| --- | --- | --- | --- | --- | --- | --- | --- | --- | --- | --- |
|  |  |  | Correct | Ambiguous | Incorrect | Correct | Ambiguous | Incorrect | No match |  |
| *Schisandra+Kadsura* | |  |  |  |  |  |  |  |  |  |
| ITS1 | 19 | 78 | 41.02 | 57.69 | 1.28 | 41.02 | 57.69 | 1.28 | 0.00 | 0.66 |
| ITS2 | 19 | 78 | 28.20 | 71.79 | 0.00 | 28.20 | 66.66 | 0.00 | 5.12 | 0.43 |
| ITS | 19 | 78 | 43.58 | 55.12 | 1.28 | 43.58 | 53.84 | 1.28 | 1.28 | 0.43 |
| *trnH-psbA* | 14 | 68 | 50.00 | 41.17 | 8.82 | 50.00 | 41.17 | 8.82 | 0.00 | 5.00 |
| *matK* | 17 | 74 | 39.18 | 60.80 | 0.00 | 39.18 | 60.80 | 0.00 | 0.00 | 0.47 |
| *rbcL* | 16 | 74 | 29.72 | 70.27 | 0.00 | 29.72 | 70.27 | 0.00 | 0.00 | 0.13 |
| ITS+*trnH-psbA* | 14 | 67 | 64.17 | 25.37 | 10.44 | 64.17 | 25.37 | 10.44 | 0.00 | 2.59 |
| ITS+*matK* | 14 | 67 | 68.65 | 23.88 | 7.46 | 68.65 | 23.88 | 7.46 | 0.00 | 0.44 |
| ITS+*rbcL* | 16 | 71 | 57.74 | 33.80 | 8.45 | 57.74 | 33.80 | 8.45 | 0.00 | 0.28 |
| *trnH-psbA+matK* | 14 | 68 | 52.94 | 38.23 | 8.82 | 52.94 | 38.23 | 8.82 | 0.00 | 2.33 |
| *trnH-psbA+rbcL* | 14 | 68 | 50.00 | 39.70 | 10.29 | 50.00 | 39.70 | 10.29 | 0.00 | 2.39 |
| *matK+rbcL* | 14 | 68 | 42.64 | 55.88 | 1.47 | 42.64 | 55.88 | 1.47 | 0.00 | 0.33 |
| ITS+*trnH-psbA*+*matK* | 14 | 67 | 79.10 | 11.94 | 8.95 | 79.10 | 11.94 | 8.95 | 0.00 | 1.71 |
| ITS+*trnH-psbA*+*rbcL* | 14 | 68 | 67.16 | 19.40 | 13.43 | 67.16 | 19.40 | 13.43 | 0.00 | 1.75 |
| ITS+*matK*+*rbcL* | 14 | 67 | 73.13 | 16.41 | 10.44 | 73.13 | 16.41 | 10.44 | 0.00 | 0.36 |
| *trnH-psbA*+*matK*+*rbcL* | 14 | 68 | 52.94 | 36.76 | 10.29 | 52.94 | 36.76 | 10.29 | 0.00 | 1.62 |
| ITS*+trnH-psbA*+*matK*+*rbcL* | 14 | 67 | 80.59 | 7.46 | 11.94 | 80.59 | 7.46 | 11.94 | 0.00 | 1.32 |
| *Illicium* |  |  |  |  |  |  |  |  |  |  |
| ITS1 | 13 | 44 | 65.90 | 34.08 | 0.00 | 63.63 | 34.08 | 0.00 | 2.27 | 1.32 |
| ITS2 | 13 | 44 | 79.54 | 11.36 | 9.09 | 79.54 | 11.36 | 9.09 | 0.00 | 3.04 |
| ITS | 13 | 44 | 84.09 | 11.36 | 4.54 | 84.09 | 11.36 | 4.54 | 0.00 | 1.58 |
| *trnH-psbA* | 11 | 38 | 84.21 | 15.78 | 0.00 | 81.57 | 15.78 | 0.00 | 2.63 | 0.17 |
| *matK* | 11 | 35 | 42.85 | 57.14 | 0.00 | 42.85 | 57.14 | 0.00 | 0.00 | 0.00 |
| *rbcL* | 11 | 39 | 5.12 | 94.87 | 0.00 | 5.12 | 94.87 | 0.00 | 0.00 | 0.13 |
| ITS+*trnH-psbA* | 10 | 32 | 100.00 | 0.00 | 0.00 | 90.62 | 0.00 | 0.00 | 9.37 | 0.31 |
| ITS+*matK* | 10 | 32 | 96.87 | 3.12 | 0.00 | 90.62 | 0.00 | 0.00 | 9.37 | 0.25 |
| ITS+*rbcL* | 11 | 34 | 97.05 | 2.94 | 0.00 | 91.17 | 0.00 | 0.00 | 8.82 | 0.28 |
| *trnH-psbA+matK* | 10 | 33 | 81.81 | 18.18 | 0.00 | 75.75 | 18.18 | 0.00 | 6.06 | 0.05 |
| *trnH-psbA+rbcL* | 11 | 38 | 84.21 | 15.78 | 0.00 | 76.31 | 15.78 | 0.00 | 7.89 | 0.06 |
| *matK+rbcL* | 10 | 33 | 57.57 | 42.42 | 0.00 | 57.57 | 42.42 | 0.00 | 0.00 | 0.00 |
| ITS+*trnH-psbA*+*matK* | 10 | 32 | 100.00 | 0.00 | 0.00 | 90.62 | 0.00 | 0.00 | 9.37 | 0.18 |
| ITS+*trnH-psbA*+*rbcL* | 11 | 38 | 100.00 | 0.00 | 0.00 | 90.62 | 0.00 | 0.00 | 9.37 | 0.20 |
| ITS+*matK*+*rbcL* | 10 | 32 | 96.87 | 3.12 | 0.00 | 90.62 | 0.00 | 0.00 | 9.37 | 0.18 |
| *trnH-psbA*+*matK*+*rbcL* | 10 | 33 | 81.81 | 18.18 | 0.00 | 75.75 | 18.18 | 0.00 | 6.06 | 0.03 |
| ITS*+trnH-psbA*+*matK*+*rbcL* | 10 | 32 | 100.00 | 0.00 | 0.00 | 90.62 | 0.00 | 0.00 | 9.37 | 0.13 |

^*^ Species represented by multiple individuals.
