# Supplementary material for: Evaluation of Four Commonly Used DNA Barcoding Loci for Chinese Medicinal Plants of the Family Schisandraceae
Source: PLoS One. 2015 May 4;10(5):e0125574. doi: 10.1371/journal.pone.0125574 (PMC4418597; doi:10.1371/journal.pone.0125574)
Supplement: S10 Table — (DOC) [file pone.0125574.s012.doc]

**S10 Table. The comparison of within and between group mean distances for *Schisandra rubriflora* and *S. grandiflora*.**

| Group | Within group mean distance (%) | | | | Between group mean distance (%) | | | |
| --- | --- | --- | --- | --- | --- | --- | --- | --- |
|  | ITS | *trnH-psbA* | *matK* | *rbcL* | ITS | *trnH-psbA* | *matK* | *rbcL* |
| *S. rubriflora* | 0. 30 | 0. 84 | 0. 25 | 0.11 | 0.27 | 0.74 | 0.21 | 0.10 |
| *S. grandiflora* | 0. 27 | 0. 80 | 0. 23 | 0.11 |  |  |  |  |
| *S. rubriflora* I | 0. 45 | 0. 41 | 0. 00 | 0.00 | 0.37 | 0.21 | 0.00 | 0.00 |
| *S. grandiflora* I | n/c | 0. 00 | 0. 00 | 0.00 |  |  |  |  |
| *S. rubriflora* II | 0. 24 | 0. 17 | 0. 07 | 0.09 | 0.24 | 0.22 | 0.06 | 0.08 |
| *S. grandiflora* II | 0. 30 | 0. 25 | 0.07 | 0.09 |  |  |  |  |
| Cluster I | 0. 40 | 0. 21 | 0.00 | 0.00 | 0.29 | 1.50^*^ | 0.43^*^ | 0.15^*^ |
| Cluster II | 0. 26 | 0.22 | 0.07 | 0.08 |  |  |  |  |

‘n/c’ indicates that only one individual was included in the group and the within group mean distance could not be calculated.

*The between group mean distance was higher than the within group mean distance for each group.
