# Supplementary material for: Evaluation of Four Commonly Used DNA Barcoding Loci for Chinese Medicinal Plants of the Family Schisandraceae
Source: PLoS One. 2015 May 4;10(5):e0125574. doi: 10.1371/journal.pone.0125574 (PMC4418597; doi:10.1371/journal.pone.0125574)
Supplement: S11 Table — (DOC) [file pone.0125574.s013.doc]

**S11 Table. The partition of distinct clusters for *Schisandra rubriflora* and *S. grandiflora*** indicated in this study.

| Individual | Province/region code | Locality |
| --- | --- | --- |
| Cluster I |  |  |
| *S. rubriflora* S027 | SC | Muli, Sichuan |
| *S. rubriflora* S6 | YN | Xianggelila, Yunnan |
| *S. grandiflora* S85 | YN | Deqin, Yunnan |
| *S. grandiflora* S88 | YN | Weixi, Yunnan |
| Cluster II |  |  |
| II-1 |  |  |
| *S. rubriflora* S075 | YN | Kunming, Yunnan |
| *S. rubriflora* S86 | YN | Fugong, Yunnan |
| *S. grandiflora* S81 | YN | Yunlong, Yunnan |
| *S. grandiflora* S031 | XZ | Motuo, Xizang |
| *S. grandiflora* Sa | XZ | Bomi, Xizang |
| II-2 |  |  |
| *S. rubriflora* S69 | SC | Emei, Sichuan |
| *S. rubriflora* S078 | SC | Leibo, Sichuan |
| *S. rubriflora* S080 | CQ | Nanchuan, Chongqing |
| *S. grandiflora* S83 | SC | Ebian, Sichuan |
| *S. grandiflora* S84 | SC | Mianning, Sichuan |
